# Broad Spectrum Enantioselective Amide Bond Synthetase from *Streptoalloteichus hindustanus*

**DOI:** 10.1021/acscatal.3c05656

**Published:** 2024-01-06

**Authors:** Qingyun Tang, Mark Petchey, Benjamin Rowlinson, Thomas J. Burden, Ian J. S. Fairlamb, Gideon Grogan

**Affiliations:** Department of Chemistry, University of York, Heslington, York YO10 5DD, U.K.

**Keywords:** biocatalysis, amide, ATP, amide bond synthetase, ligase

## Abstract

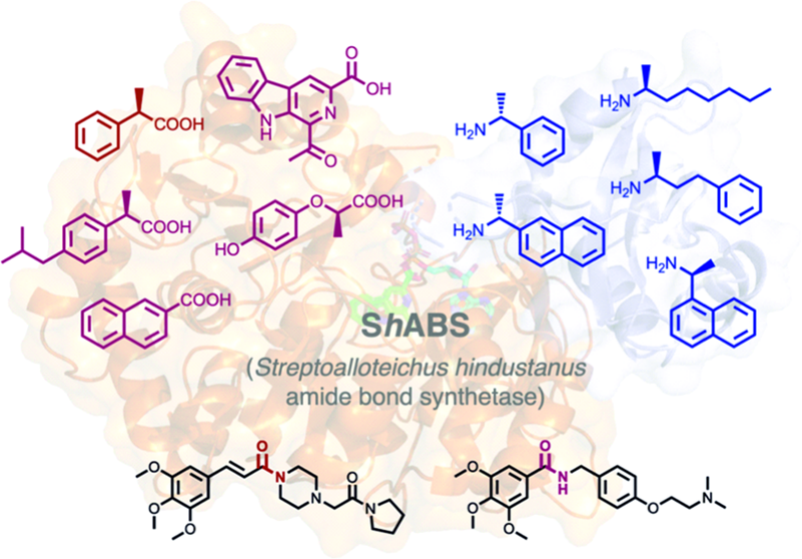

The synthesis of amide bonds is one of the most frequently performed reactions in pharmaceutical synthesis, but the requirement for stoichiometric quantities of coupling agents and activated substrates in established methods has prompted interest in biocatalytic alternatives. Amide Bond Synthetases (ABSs) actively catalyze both the ATP-dependent adenylation of carboxylic acid substrates and their subsequent amidation using an amine nucleophile, both within the active site of the enzyme, enabling the use of only a small excess of the amine partner. We have assessed the ability of an ABS from *Streptoalloteichus hindustanus* (ShABS) to couple a range of carboxylic acid substrates and amines to form amine products. ShABS displayed superior activity to a previously studied ABS, McbA, and a remarkable complementary substrate specificity that included the enantioselective formation of a library of amides from racemic acid and amine coupling partners. The X-ray crystallographic structure of ShABS has permitted mutational mapping of the carboxylic acid and amine binding sites, revealing key roles for L207 and F246 in determining the enantioselectivity of the enzyme with respect to chiral acid and amine substrates. ShABS was applied to the synthesis of pharmaceutical amides, including ilepcimide, lazabemide, trimethobenzamide, and cinepazide, the last with 99% conversion and 95% isolated yield. These findings provide a blueprint for enabling a contemporary pharmaceutical synthesis of one of the most significant classes of small molecule drugs using biocatalysis.

## Introduction

The synthesis of amide bonds is one of the most important reactions in pharmaceutical synthetic chemistry^1^ and has been suggested to account for up to 16% of all reactions performed in relevant laboratories.^2^ Although methods of amide bond syntheses are straightforward, they often require the activation of the carboxylic acid prior to amide bond formation, using a coupling reagent that is required in stoichiometric amounts.^3,4^ In addition to poor atom economy, many of the reagents and methods used can be toxic or otherwise hazardous. In recognition of these practical limitations, there has been ongoing interest in the applications of enzymes in the synthesis of amide bonds,^5−10^ as these operate in the absence of hazardous chemical coupling agents and often in an aqueous environment. Of the enzymes that have been investigated for preparative amide bond formation, lipases often require the esterification of substrate carboxylic acids prior to amide coupling.^11,12^ However, in a recent example, a highly efficient lipase SpL from *Sphingomonas* sp. HXN-200, which amidates both esters and free carboxylic acids, has been reported by Li and co-workers.^13^ In addition, acylases such as MsACT from *Mycobacterium smegmatis* have been shown to synthesize amides from esters and amines in aqueous media if the amine is provided in a large excess.^14,15^

In addition to hydrolases, various ATP-dependent enzymes have been studied for application in the synthesis of amides.^8,9^ Philpott and co-workers constructed a whole-cell system in *Escherichia coli* in which ATP-dependent acyl-CoA ligases and *N-*acyl transferases were coexpressed for the transformation of acids to amides via adenylate and CoA intermediates in vivo.^16^ In other cases, the activation of carboxylic acids using ATP to form intermediate adenylates in vitro, by the adenylation domains of nonribosomal peptide synthase (NRPS) enzymes,^17^ carboxylic acid reductases (CARs),^18^ and others^19−21^ have been applied, in which the adenylate can be intercepted by an amine nucleophile, provided in large excess, to form amide products. In one example, the adenylation domain of a CAR was applied to the synthesis of the antiepileptic drug ilepcimide from 3,4-(methylenedioxy)cinnamic acid and piperidine, although it was necessary to employ a large 100-fold excess of amine.^22^ The CAR adenylation system was also applied to the monoamidation of diamines^23^ and also recently coupled to *N*-acyl transferases in a whole-cell system for the synthesis of a range of amide products.^24^ The large excess of amine required in examples that exploit adenylation enzymes in vitro as the first step of amide bond formation is thought to be required to drive the amination part of the reaction, which is not enzyme-catalyzed but rather occurs in solution between the adenylate and the amine partner.

**Scheme 1:**
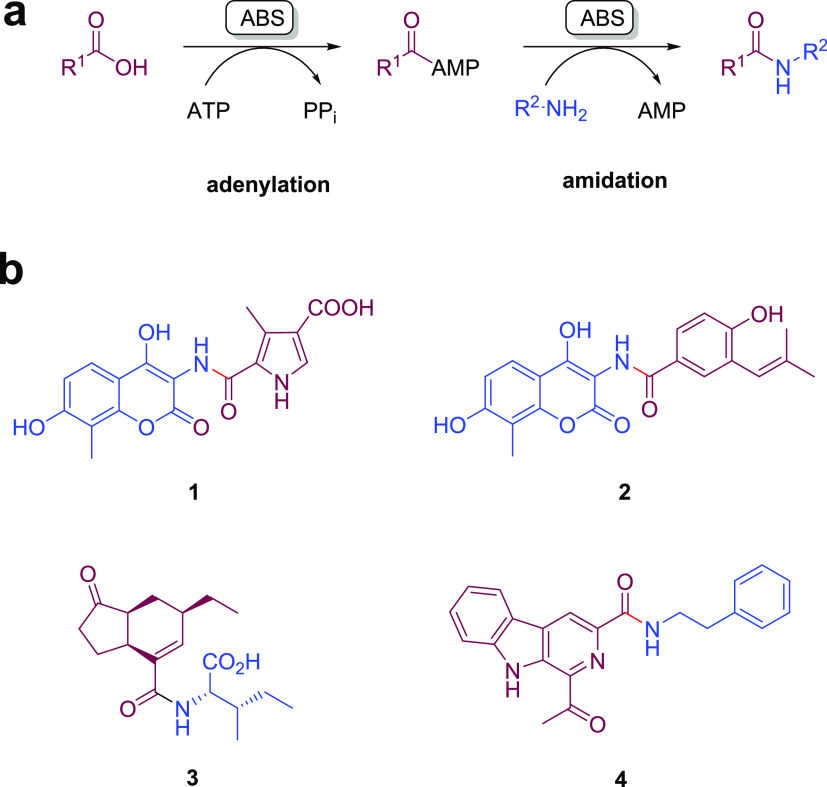
(a) Activity of Amide Bond Synthetases (ABSs); (b) Products of ABS-Catalyzed Reactions in the Formation of Amides in Biosynthetic Pathways toward Coumermycin (**1**), Novobiocin (**2**), Coronatine (**3**), and Marinacarboline (**4**)

The requirement for large excesses of amine in these reactions has prompted investigations into the ATP-dependent amide bond synthetase (ABS) class of enzymes, also members of the larger ANL (**a**cyl-CoA synthetase–non-ribosomal peptide synthase–**l**uciferase) family of adenylase enzymes,^25^ as these catalyze both the formation of the adenylate and the amidation reaction within one active site of the enzyme (Scheme 1) and require only one or low equivalents of the amine as a coupling partner.

**Scheme 2:**
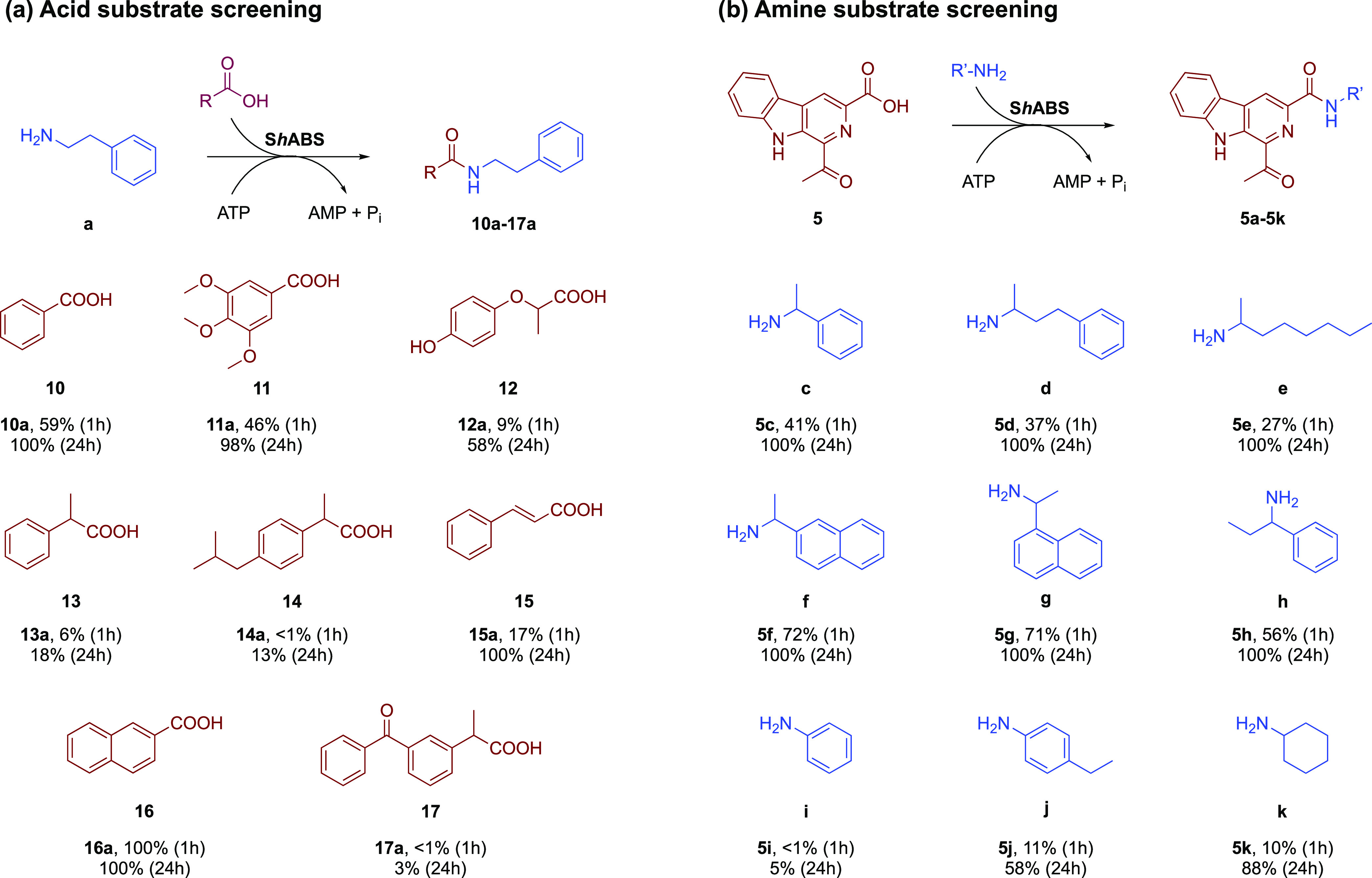
Substrate Screening of ShABS (a): Amine **a** was used for carboxylic acid substrate screening; (b) Acid **5** was used for amine substrate screening. For chiral substrates, racemic compounds were applied in reactions. Reactions were carried out with 0.4 mM β-carboline acids, 0.6 mM amine (1.5 equiv), 0.8 mM ATP (2 equiv), 2 U mL^–1^ inorganic phosphatase (IPase), and 1 mg mL^–1^ ABS in 50 mM NaPi buffer, pH 7.5 at 37 °C with orbital shaking at 800 r.p.m.

ABSs catalyze the formation of intermediates in the biosynthesis of the secondary metabolites coumermycin (CouL, intermediate **1**, Scheme 1) novobiocin (NovL, intermediate **2**, Scheme 1),^26^ coumermycin (CouL),^27^ clorobiocin (CloL),^28^ and simocyclinone D_8_ (SimL).^29,30^ Further ABSs from coronatine biosynthesis in *Pseudomonas syringae*,^31^ such as PsCfaL, have recently been applied to the synthesis of amino acid-coupled products (Scheme 1).^32^ McbA, first described by Ji and co-workers,^33^ is an ABS from the marine actinomycete *Marinactinospora thermotolerans* that catalyzes the coupling of a β-carboline acid and 2-phenylethylamine to form amide **4** (Scheme 1) as part of the biosynthetic pathway toward the marinacarboline antibiotics. In previous work, we showed that McbA could be applied to the coupling of a wide range of amine and carboxylic acid partners.^34^ In terms of carboxylic acid specificity, we determined that this property extended to derivatives of the native carboxylic acid substrate with substitutions proximal to the pyridyl nitrogen, ranging from -H to benzoyl, but also to simpler bicyclic and monocyclic acids, including benzoic acid, with 2-phenylethylamine supplied in only 1.5 mol equiv amounts.^34^ The structure of McbA in complex with the native carboxylic acid **5** (Table 1 and Scheme 2) and AMP was determined.^34^ In common with other adenylases,^25^ McbA features a larger N-terminal domain coupled to a C-terminal “cap” domain. The structure revealed two conformations: the first, McbA_Ad_, superimposed well with adenylases that were determined in the “adenylation” conformation, such as 4-chlorobenzoyl-CoA ligase (4CBCL),^35,36^ organized for the adenylation reaction; the second McbA_Am_, superimposed well with 4CBCL in its “thiolation” conformation, the second half-reaction in 4CBCL,^36^ in which a rotation of approximately 140° of the cap domain permits access of the nucleophile—the amine in McbA—for the amidation half of the reaction.

**Table 1:**
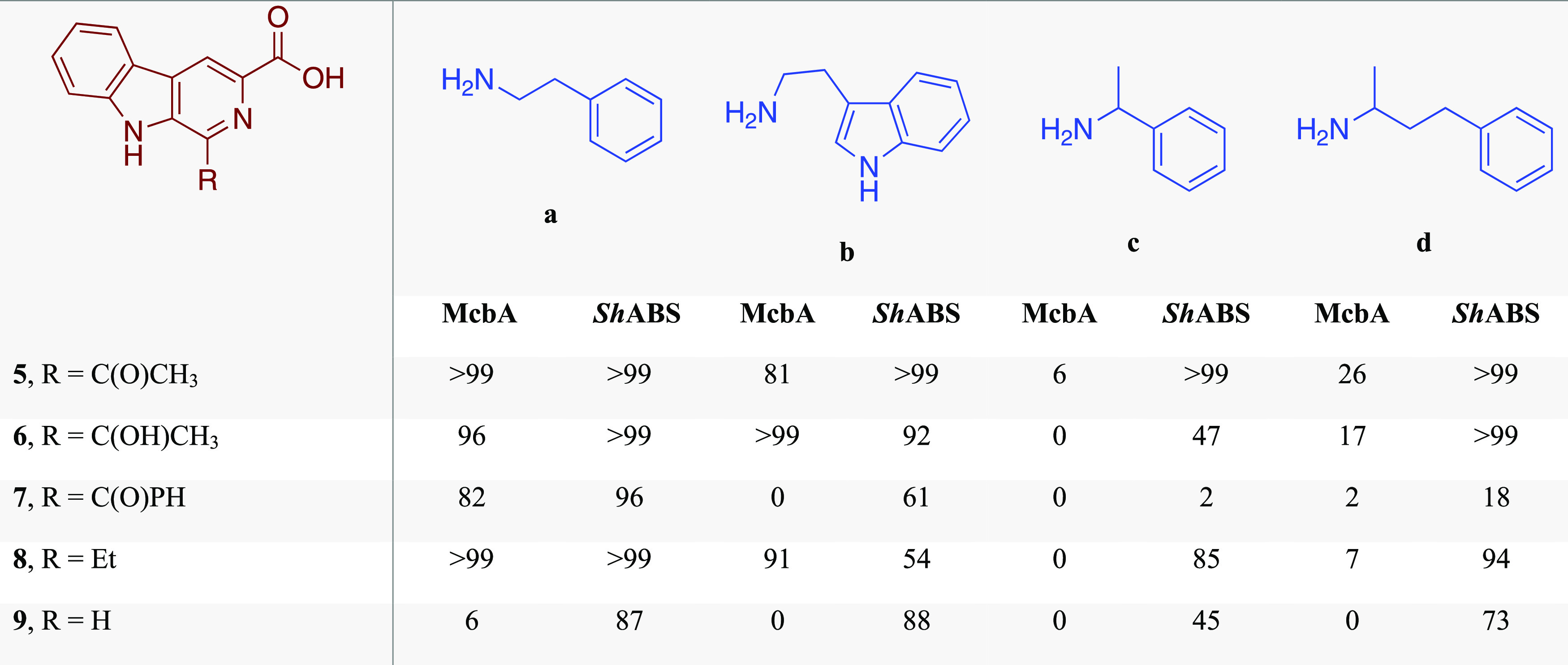
Amide Coupling of a Series of β-Carboline Acids **5**–**9** with Amine Partners **a**–**d** Catalyzed by Either McbA or ShABS^a^

a Numbers refer to conversions in % measured by HPLC after 24 h incubation. Reactions were carried out with 0.4 mM β-carboline acids, 0.6 mM amine (1.5 equiv), 0.8 mM ATP (2 equiv), 2 U mL^–1^ inorganic phosphatase (IPase), and 1 mg mL^–1^ ABS in 50 mM NaPi buffer, pH 7.5 at 37 °C with orbital shaking at 800 r.p.m.

The broad carboxylic acid specificity of McbA appeared to be attributable to a large hydrophobic active site, in which few specific interactions with functional groups on the acid substrate were in evidence. It was also noted that H207 in 4CBCL, thought to have a role in catalysis in that enzyme,^35,36^ was replaced by an aspartate residue D201 in McbA. Mutation of D201 to alanine resulted in a variant of low activity, and this result, with modeling studies, suggested that this residue may have a role in the activation of the incoming amine nucleophile for the amidation reaction.^37^ This study also determined that the amine specificity of McbA extended beyond substrates related to 2-phenylethylamine, to include amines ranging from methylamine to benzylamine, and that, consequently, McbA could be applied to the multimilligram synthesis of pharmaceutically relevant amides including the monoamine oxidase A inhibitor moclobemide.^37^ The synthetic potential of wild-type McbA prompted us to investigate the activity of related ABS enzymes in order to unearth improved and wider-ranging activities for biocatalytic amidation. In this report, we describe the activity and specificity of an ABS from *Streptoalloteichus hindustanus* (ShABS), with a broader, complementary substrate spectrum to that of McbA that includes enantioselective behavior toward an expanded range of acid and amine partners in amidation reactions. Determination of the X-ray crystal structure of ShABS has also permitted an investigation of the molecular determinants of enantioselectivity in the active site for each partner, shedding new light on the specificity within this potentially valuable class of enzymes.

## Results and Discussion

A search of available databases using the McbA sequence as the model determined that there were only a handful of homologous sequences that displayed above 50% sequence identity with McbA. These included hypothetical “AMP-binding proteins” from *Actinoalloteichus cyanogriseus* (AcABS; GenBank accession number: WP_051713392.1; 54% sequence identity with McbA), which has recently been studied by Zhang and co-workers,^38^ and *S. hindustanus* (ShABS; WP_073481158.1; 51%). Compared to other families of ABS enzymes, *Sh*ABS (Figure S1) displayed 34% sequence identity with NovL and 24% with PsCfaL (Figure S2). A phylogenetic tree clearly revealed that ShABS-family ABSs were of a distinct subfamily compared to NovL-type and CfaL-type enzymes (Figure S3).

The gene encoding ShABS (Figure S4) was cloned and expressed in *E. coli* BL21 (DE3) in LB medium using a pET-28a(+) vector. Purification using nickel affinity and size exclusion chromatography yielded ShABS with a molecular weight of approximately 54 kDa (Figure S5a,b) that appeared to be monomeric in solution, according to gel filtration results. The activity was initially tested against the McbA native β-carboline acid substrate **5** and 1.5 equiv of phenylethylamine **a**, with the addition of 2 equiv of ATP (Table 1). HPLC analysis confirmed >99% conversion in 24 h to the amide product **4**, the same product observed with both McbA and also AcABS reported by Zhang and co-workers.^38^ The confirmed activity of ShABS prompted us to challenge the enzyme with a small library of modified β-carboline acid substrates **5**–**9** and amines **a**–**d** that had been tested previously with McbA (Table 1).^34^ The most notable differences between McbA and ShABS activity were observed for aminations of substrate **5, 6**, and **8** with 1-phenylethylamine **c** and 4-phenylbutan-2-amine **d**, for which ShABS gave high conversions, whereas McbA gave negligible or low values. In addition, ShABS gave higher conversions for the acid substrate **9**, with an H in the 1-position of the β-carboline ring, with all four amines, where again McbA gave no conversion.

The broader substrate specificity of ShABS for β-carboline acids prompted a survey of a wider spectrum of carboxylic acid substrates, ranging from simple benzoic acid **10** to chiral Profen derivatives such as **14** and larger substrates such as 2-naphthoic acid **16** (Scheme 2a). Reactions were monitored using HPLC with products identified by comparison with synthesized amide products as standards (SI Section 3). Simple acids such as benzoic acid **10** were coupled rapidly, with >99% conversion to amide products within 1 h, while McbA gave only 24 and 43% conversions, respectively, after 24 h. 3,4,5-Trimethoxybenzoic acid **11** gave 46% conversion after 1 h. Chiral acids with a methyl group in the benzylic position such as α-methylbenzoic acid **13** and Profen **14** were consumed more slowly, although incubation times of 24 h resulted in conversions of 18 and 13%, respectively. In addition, *trans*-cinnamic acid **15** was efficiently converted to amide **15a** with >99% conversion after 24 h.

Following the acid screen, ShABS was then challenged with a series of amines using β-carboline **5** as the acid partner. (Scheme 2b). As with McbA, ShABS exhibited substantial activity with amines other than phenylethylamine **a**; chiral amines 1-phenylethylamine **c**, 4-phenylbutan-2-amine **d**, and 2-amino octane **e** were coupled to **5** with 41, 37, and 27% conversion, respectively, after 1 h. Even larger chiral amines such as 1-(2-naphthyl)ethylamine **f** and 1-(1-naphthyl)ethylamine **g** gave 72 and 71% conversion to amide products after 1 h. The less nucleophilic anilines were less well transformed, with aniline **i** and 4-ethyl aniline **j** only giving conversions of 5 and 58% after 24 h.

**Figure 1:**
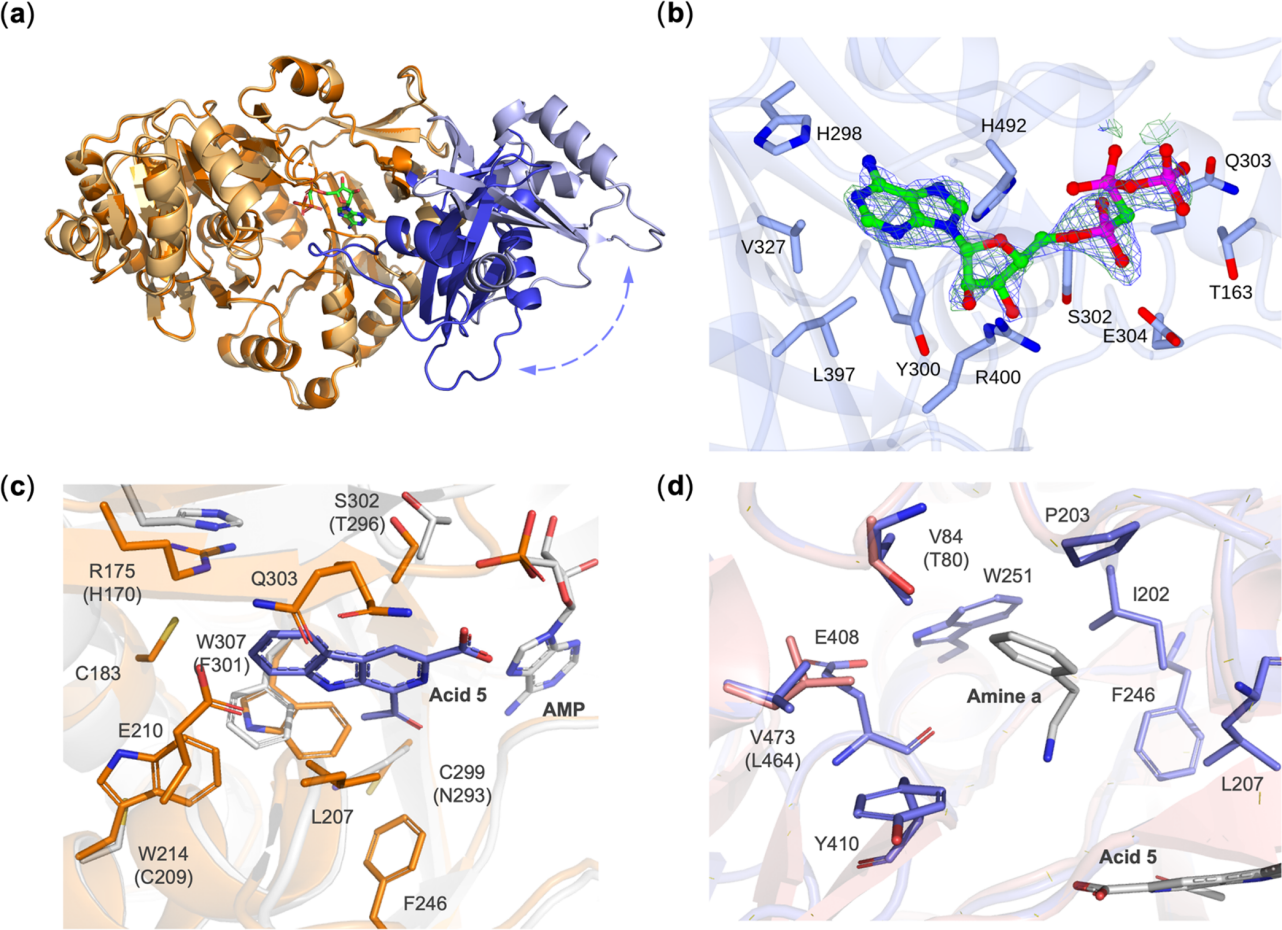
(a) Superimposition of ShABS structures in the adenylation conformation (ShABS_Ad_; adenylation domain in orange and cap domain in blue) in complex with AMP-CPP (carbon atoms in green) and the *apo*- open conformation (ShABS_open_; adenylation domain in light orange and cap domain in light blue). The rotation of the cap domain is indicated by the arrow; (b) electron density for AMP-CPP in the ShABS_Ad_ active site. Electron density corresponds to the 2*F*_o_–*F*_c_ (blue) and *F*_o_–*F*_c_ (green) omit maps at levels of 1σ and 3σ, respectively, obtained prior to inclusion of the ligand in refinement; (c) superimposition of active sites of McbA_Ad_ (carbon atoms in white) in complex with **5** and AMP-CPP and ShABS_Ad_ (carbon atoms in orange, AMP-CPP removed); only amino acid residues that differ between the enzymes are shown. (d) Amine binding tunnel of ShABS from a model of ShABS_Am_ (carbon atoms in purple) created using McbA_Am_ (pink) as a model.

In order to explore the basis for the broader substrate specificity of ShABS, the three-dimensional structure was determined by using X-ray crystallography (SI Section 4). As crystals of wild-type ShABS were initially not forthcoming, two mutants, K492A and K492H, of a lysine residue K492 implicated in interacting with ATP were prepared and subjected to crystallization trials, in an attempt to obtain a structure. Although no crystals were obtained in the presence of ATP or AMP and acid substrate, the inclusion of the noncleavable ATP analogue α,β-methylene adenosine 5′-triphosphate (AMP-CPP) was successful in giving diffracting crystals of ShABS K492H in the *P*2_1_ space group, from which were obtained data sets that were refined to 2.01 (Data set #1) and 2.57 Ångstrom (Data set #2) resolution, the second of which contained density for AMP-CPP in the active site. This structure of ShABS (ShABS_Ad_Figure 1a) featured two molecules in the asymmetric unit cell, each of which corresponded to the “adenylation” conformation of McbA, with an rmsd between the two enzymes of 1.50 Å over 469 C-α atoms. Later, an additional structure of ShABS was also obtained from crystallization of the wild-type enzyme in space group *P*6_3_22 (Data set #3), which had been cocrystallized with the amide product **10a**. This ShABS structure (ShABS_open_) displayed a rotation, as determined using the Dyndom server,^39^ of the cap domain of 81° in the opposite direction to the rotation observed between McbA_Ad_ and McbA_Am_, to expose the active site completely. Although no electron density for a ligand was observed, as the enzyme in these experiments had been complexed with the amide product, we speculate that this conformation may be representative of an exit protein-product complex assumed by ShABS following product release.

In the structure of ShABS_Ad_ obtained from Data set #1, following the building of the protein and water atoms, residual density in the omit maps was clearly visible, and this could be successfully modeled and refined as α,β-methylene adenosine 5′-triphosphate (AMP-CPP, Figure 1b).

Superimposition of ShABS_Ad_ and McbA_Ad_ permitted the positioning of the acid substrate **5** within the active site and a consequent analysis of probable active site interactions (Figure 1c).

The major differences in active site residues between the ShABS_Ad_ and McbA_Ad_ acid binding sites were W307 (ShABS) for F301 (McbA), W214 for C209, R175 for H170, C299 for N293, and S302 for T296. The presence of the two tryptophan residues is most likely to account for the differences in acid specificity between McbA and ShABS. The substitution of F301 for W307 changes the active site environment at C1 of the β-carboline acid and perhaps accounts for the improved activity of ShABS for substrates modified at C1, especially **9**, where the acyl group is replaced by a proton. W214 also increases the hydrophobicity of the binding pocket, replacing Cys and also displacing E210 (E205) from the active site. It is also conceivable that, while the two Trp residues decrease the pocket size, they may also push the acid substrate toward ATP and the amine pocket, with potential effects on each half-reaction.

Although a structure of ShABS in the amination conformation (‘ShABS_Am_’) was not determined, a homology model could be constructed using the SWISS-MODEL server^40^ with the structure of McbA_Am_ as a template (SI Section 4.2). In the amine binding tunnel identified in McbA_Am,_^37^ residues were much more conserved in ShABS than in the acid binding pocket. These residues included ShABS V84 (T80 McbA); I202 (I197); P203 (P198); L207 (L202); F246 (F241); W251 (W246); E408 (E400); Y410 (Y402); and V473 (L464). This suggests that there must be little discrimination of amine binding between the enzymes in the amine tunnel. The observed experimental differences in amine specificity therefore probably arise as a result of constraints on interactions of the amine with the adenylate intermediate. This is supported by several results in the acid and amine screens in Table 1. For example, the ShABS-catalyzed coupling of acid **5** and chiral amines **c** and **d**, with conversions of >99% in each case, suggests that these amines are bound efficiently by the enzyme. Equally, the coupling of substrate **7**, with the larger benzoyl group in the 1-position, proceeds to 96% conversion with amine **a**, suggesting that **7** is a good acid substrate. However, the attempts to combine acid **7** with amines **c** or **d** give only low conversions of 2 and 18%, respectively, suggesting that it is the constraints imposed by the binding of the adenylate of **7** that result in this case, specifically the inability to efficiently bind and transform those amines.

Following our observation that McbA would discriminate enantiomers of amine **d** in the coupling reaction with **5**,^34^ we investigated the enantioselective properties of ShABS with both acid and amine substrates. When challenged with racemic acid substrates **12**, **13**, and **14** and 1-phenylethylamine **a**, ShABS exhibited enantioselectivity, giving (*R*)- amide products with enantiomeric excess (ee) values of 40, 32, and 83% at reaction conversions of 18, 13, and 43% respectively (Table 2**;** chiral HPLC chromatograms are shown in SI Section 8). Moreover, when using acid **5** and a range of chiral amines **c**–**h**, we determined that ShABS indeed exhibited enantiodiscrimination of these amines with ee values ranging from 21% at 59% conversion for amide **5e** using an aliphatic chiral amine to 74 and 67% ee for amides **5c** and **5h** at reaction conversions of 43 and 44%, respectively (Table 2).

**Table 2:** Enantioselective Amide Couplings Catalyzed by *Sh*ABS^a^

acid	amine	conversion (%)	amide product	amide ee (%)	E*
**12**	**a**	43	(*R*)-**12a**	82	19
**13**	**a**	18	(*R*)-**13a**	40	n.d.**
**14**	**a**	13	(*R*)-**14a**	34	n.d.**
**5**	**g**	56	(*S*)-**5g**	43	4
**5**	**d**	22	(*S*)-**5d**	24	n.d.**
**5**	**e**	59	(*S*)-**5e**	21	2
**5**	**c**	43	(*R*)-**5c**	74	12
**5**	**h**	44	(*R*)-**5h**	67	8
**5**	**f**	37	(*R*)-**5f**	28	2

a Reactions were carried out in the same conditions as Table 1, except that 0.1 mg mL^–1^ ShABS was used for the production of **5g–5h**. *E = ln[(1 – *c*)(1 + ee(*P*))]/ ln[(1 – c)(1 – ee(*P*))];^41^ ** not determined, as it recommended that *E* values only be determined when conversion is >30%.

**Figure 2:**
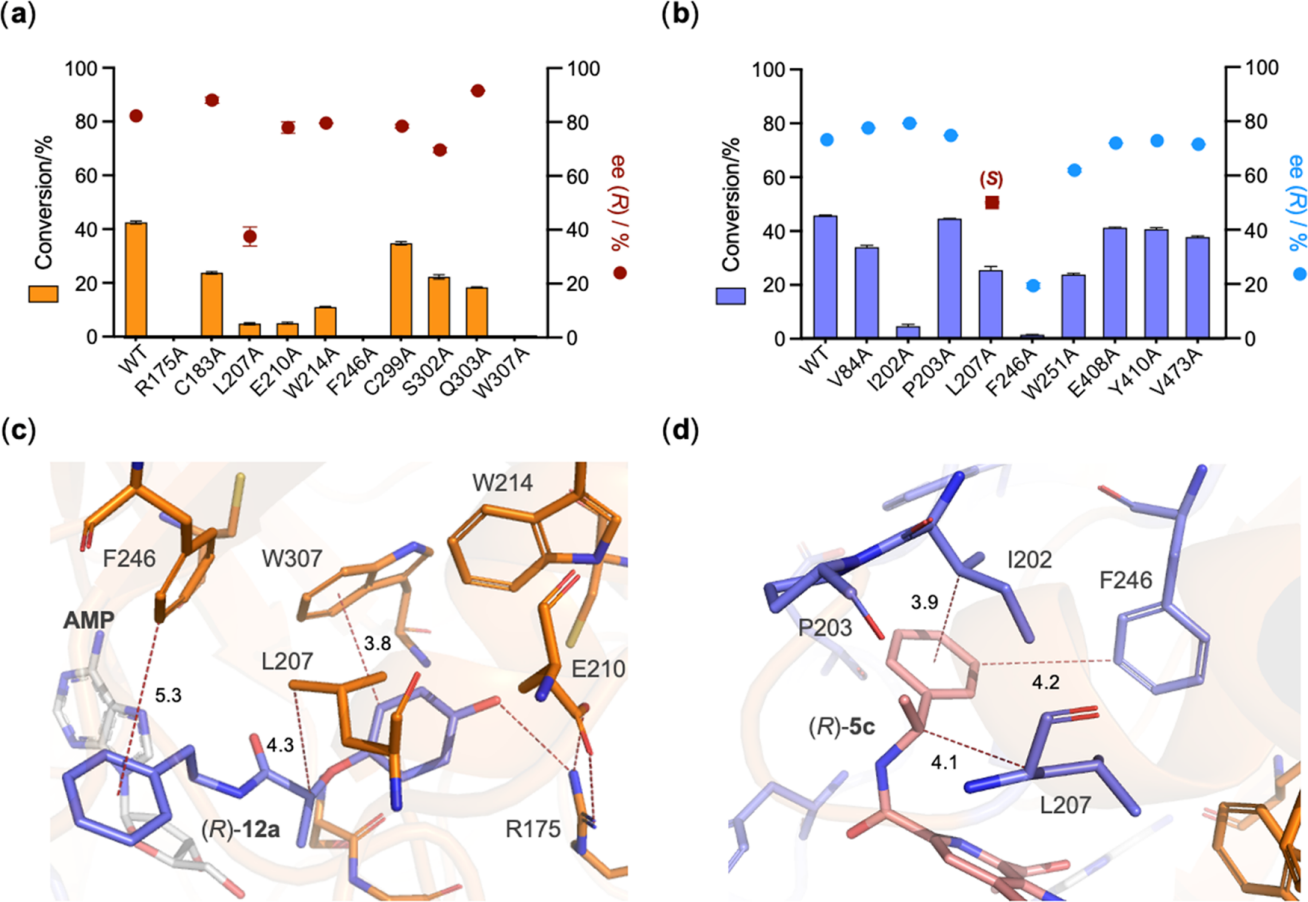
(a) Activities and product ees of wild-type ShABS and mutants in the carboxylic acid substrate binding pocket for the coupling of *rac*-**12** and amine **a**; (b) activities and product *ees* of wild-type ShABS and mutants in the amine substrate binding pocket for the coupling of acid **5** and *rac*-**c**. (c) Docking of the favored product enantiomer (*R*)-**12a** (carbon atoms in purple) in the active site of ShABS; (d) docking of the favored product enantiomer (*R*)-**5c** (carbon atoms in pink) in the active site of ShABS.

Using the structure of ShABS as a guide, a mutational analysis was then performed, with a view to mapping the active site of ShABS with respect to the enantioselectivity of the enzyme, the first such study performed for an ABS. First, mutations were made within the carboxylic acid binding pocket. Residues R175, C183, L207, E210, W214, F246, C299, S302, Q303, and W307 were all mutated to alanine individually. ShABS muteins were expressed and purified as for the wild-type enzyme and assayed with 0.4 mM racemic acid **12** and 0.6 mM 2-phenylethylamine **a** as the amine partner. Reactions were arrested midway through conversion so as to maximize the opportunity to measure the enantioselectivity (Figure 2a). Mutation of R175, F246, and W307 to alanine resulted in inactive mutants. R175 and F246 do not appear to have a significant role in substrate binding, although W307 is one of the crucial active site residues interacting with the acid substrate, as described above.

Conversions for other muteins were largely between 10 and 35%, and the *ee*s were mostly within ±10% of the 82% (43% conversion) observed with the wild-type. Of these, C183A and Q303A gave 88 and 92% ee at 23 and 18% conversion, respectively. However, for L207A, ee was markedly reduced, with only 37% ee observed for the amide product at 5% conversion.

A model of the favored product (*R*)-**12a** in the active site of ShABS (Figure 2c), created using Autodock Vina,^42^ suggests that L207 would indeed be close to the chiral methyl group of **12**, disfavoring the binding of the nonpreferred (*S*)-enantiomer of the acid.

Mutations in the amine binding tunnel of ShABS were performed to give variants with additional residues V84, I202, 203, W251, E408, Y410, and V473 mutated to alanine individually. Each was expressed, purified, and assayed with the native β–carboline acid **5** and racemic 1-phenylethylamine **c** in addition to muteins L207A and F246A already prepared (Figure 2b). Conversion was again poor for F246A, but also I202A. Other mutants gave broadly similar conversions to the WT (43%), and ee values were again within ±10% of the WT (74% ee), with the exception of F246A, with a low ee of 19% even at low conversion (2%), and also L207A, for which enantioselectivity was inverted, giving in this case the (*S*)-amide with 50% ee. Docking of the preferred product enantiomer (*R*)-**5c** (Figure 2d) again suggested that L207 would be situated near the chiral methyl group, again potentially disfavoring the binding of the (*S*)-amine for amide coupling in the wild-type.

**Scheme 3:**
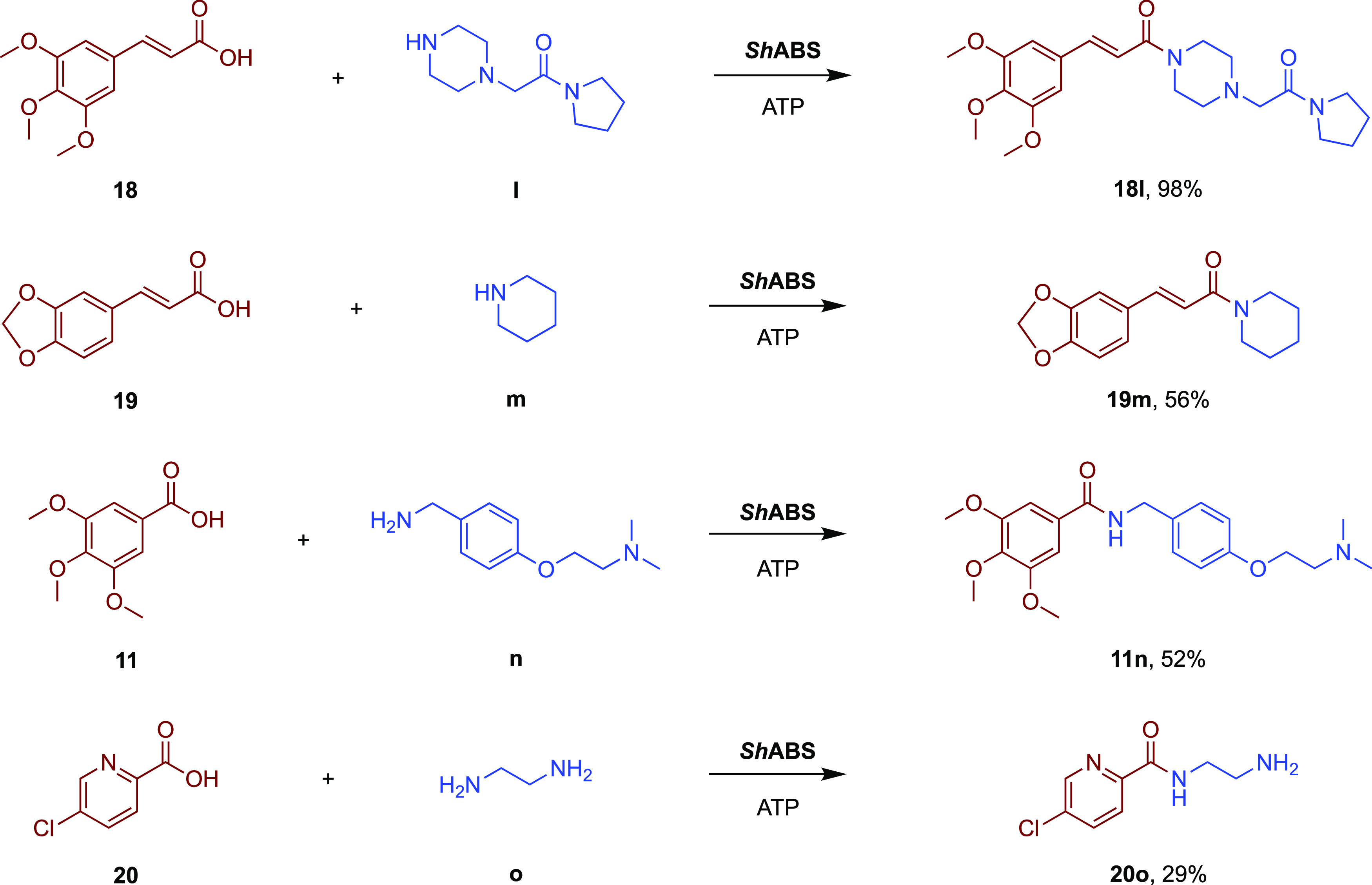
Pharmaceutical Amides Synthesized by ShABS Reactions were carried out with 5 mM carboxylic acids, 10 mM amine (2 equiv), 10 mM ATP (2 equiv), 2 U mL^–1^ IPase, and 2 mg mL^–1^ ABS in 50 mM NaPi buffer, pH 7.5 at 37 °C with orbital shaking at 800 r.p.m for 48 h.

The broader substrate specificity of ShABS was exploited in the synthesis of selected key pharmaceutical targets from commercially available carboxylic acid and amine precursors. Several pharmaceutical amides with scaffolds close to the substrate scope of ShABS were selected as targets (Table S5), with the most successful reactions being performed with acids **18** to **20** and **11** and amines **l**–**o** that yield the pharmaceutical products cinepazide **18l**, ilepcimide **19m**, trimethobenzamide **11n**, and lazabemide **20o** (Scheme 3). Using 5 mM acid substrate, 2 equiv of amine substrate, and ShABS at 2 mg mL^–1^, conversions of 98, 56, 52, and 29% were observed after 48 h (Figure S7). The synthesis of cinepazide was performed on a scale using 50 mg (0.21 mmol) of carboxylic acid **18**, 10 mM amine **I**, and 10 mM ATP, giving 99% conversion and an isolated yield of 95%. The scale-up results demonstrate the feasibility and promise of the biocatalytic synthetic methodology for a challenging tertiary amide product.

## Conclusions

The synthesis of amides presents sustainability issues that may, in part, be addressed by the use of enzymes. The catalytic activity of amide bond synthetases has great potential as part of the toolbox for enzymatic synthesis, especially as they are active in aqueous medium, only require stoichiometric amounts of coupling partners, and can also couple carboxylic acids and amines directly, complementing the activity of hydrolytic enzymes such as lipases. Mutational studies of the kind presented here illustrate that the potential exists to alter both substrate specificity and enantioselectivity of the enzymes with respect to both carboxylic acid and amine coupling partners. Future engineering experiments will focus on further expansion of catalytic scope but also stability with respect to process considerations, including the intensification of substrate loading and also cofactor recycling.

## Experimental Section

For full details of experimental procedures, see the Supporting Information.

## Supporting Information Available

Gene and amino acid sequences; cloning, expression, and purification of enzymes; site-directed mutagenesis; synthesis of product standards; crystallization of enzymes, data collection, and refinement tables; molecular docking; details of biotransformations with chromatographic analysis, including chiral analysis of relevant products; and NMR spectra (PDF)

## References

[ref1] Pattabiraman V. R.; Bode J. W. Rethinking Amide Bond Synthesis. Nature 2011, 480, 471–479. 10.1038/nature10702. 22193101

[ref2] Dunetz J. R.; Magano J.; Weisenburger G. A. Large-scale Applications of Amide Coupling Reagents for the Synthesis of Pharmaceuticals. Org. Process Res. Dev. 2016, 20, 140–177. 10.1021/op500305s.

[ref3] El-Faham A.; Albericio F. Peptide Coupling Reagents, More Than a Letter Soup. Chem. Rev. 2011, 111, 6557–6602. 10.1021/cr100048w. 21866984

[ref4] Valeur E.; Bradley M. Amide Bond Formation: Beyond the Myth of Coupling Reagents. Chem. Soc. Rev. 2009, 38, 606–631. 10.1039/B701677H. 19169468

[ref5] Goswami A.; Van Lanen S. G. Enzymatic Strategies and Biocatalysts for Amide Bond Formation: Tricks of the Trade Outside of the Ribosome. Mol. Biosyst. 2015, 11, 338–353. 10.1039/C4MB00627E. 25418915 PMC4304603

[ref6] Pitzer J.; Steiner K. Amides in Nature and Biocatalysis. J. Biotechnol. 2016, 235, 32–46. 10.1016/j.jbiotec.2016.03.023. 26995609

[ref7] Dorr B. M.; Fuerst D. E. Enzymatic Amidation for Industrial Applications. Curr. Opin. Chem. Biol. 2018, 43, 127–133. 10.1016/j.cbpa.2018.01.008. 29414531

[ref8] Petchey M. R.; Grogan G. Enzyme-catalysed Synthesis of Secondary and Tertiary Amides. Adv. Synth. Catal. 2019, 361, 3895–3914. 10.1002/adsc.201900694.

[ref9] Winn M.; Richardson S. M.; Campopiano D. J.; Micklefield J. Harnessing and Engineering Amide Bond Forming Ligases for the Synthesis of Amides. Curr. Opin. Chem. Biol. 2020, 55, 77–85. 10.1016/j.cbpa.2019.12.004. 32058241

[ref10] Lubberink M.; Finnigan W.; Flitsch S. L. Biocatalytic Amide Bond Formation. Green Chem. 2023, 25, 2958–2970. 10.1039/D3GC00456B.

[ref11] Gotor V. Non-conventional Hydrolase Chemistry: Amide and Carbamate Bond Formation Catalyzed by Lipases. Bioorg. Med. Chem. 1999, 7, 2189–2197. 10.1016/S0968-0896(99)00150-9. 10579525

[ref12] Lima R. N.; dos Anjos C. S.; Orozco E. V. M.; Porto A. L. M. Versatility of Candida antarctica Lipase in the Amide Bond Formation Applied in Organic Synthesis and Biotechnological Processes. Mol. Catal. 2019, 466, 75–105. 10.1016/j.mcat.2019.01.007.

[ref13] Zeng S.; Liu J.; Anankanbil S.; Chen M.; Guo Z.; Adams J. P.; Snajdrova R.; Li Z. Amide Synthesis via Aminolysis of Ester or Acid with an Intracellular Lipase. ACS Catal. 2018, 8, 8856–8865. 10.1021/acscatal.8b02713.

[ref14] Contente M. L.; Pinto A.; Molinari F.; Paradisi F. Biocatalytic N-acylation of Amines in Water Using an Acyltransferase from Mycobacterium smegmatis. Adv. Synth. Catal. 2018, 360, 4814–4819. 10.1002/adsc.201801061.

[ref15] Müller H.; Godehard S. P.; Palm G. J.; Berndt L.; Badenhorst C. P. S.; Becker A.-K.; Lammers M.; Bornscheuer U. T. Discovery and Design of Family VIII Carboxylesterases as Highly Efficient Acyltransferases. Angew. Chem., Int. Ed. 2021, 60, 2013–2017. 10.1002/anie.202014169. PMC7894173 33140887

[ref16] Philpott H. K.; Thomas P. J.; Tew D.; Fuerst D. E.; Lovelock S. L. A Versatile Biosynthetic Approach to Amide Bond Formation. Green Chem. 2018, 20, 3426–3431. 10.1039/C8GC01697F.

[ref17] Winn M.; Fyans J. K.; Zhuo Y.; Micklefield J. Recent Advances in Engineering Nonribosomal Peptide Assembly Lines. Nat. Prod. Rep. 2016, 33, 317–347. 10.1039/C5NP00099H. 26699732

[ref18] Gahloth D.; Dunstan M. S.; Quaglia D.; Klumbys E.; Lockhart-Cairns M. P.; Hill A. M.; Derrington S. R.; Scrutton N. S.; Turner N. J.; Leys D. Structures of Carboxylic Acid Reductase Reveal Domain Dynamics Underlying Catalysis. Nat. Chem. Biol. 2017, 13, 975–981. 10.1038/nchembio.2434. 28719588 PMC5563451

[ref19] Abe T.; Hashimoto Y.; Sugimoto S.; Kobayashi K.; Kumano T.; Kobayashi M. Amide Compound Synthesis by Adenylation Domain of Bacillibactin Synthetase. J. Antibiot. 2017, 70, 435–442. 10.1038/ja.2016.117. 27731335

[ref20] Hara R.; Hirai K.; Suzuki S.; Kino K. A Chemoenzymatic Process for Amide Bond Formation by an Adenylating Enzyme-mediated Mechanism. Sci. Rep. 2018, 8, 2950 10.1038/s41598-018-21408-8. 29440726 PMC5811625

[ref21] Marchetti P. M.; Richardson S. M.; Kariem N. M.; Campopiano D. J. Synthesis of N-acyl Amide Natural Products Using a Versatile Adenylating Biocatalyst. MedChemComm 2019, 10, 1192–1196. 10.1039/C9MD00063A. 31741729 PMC6677021

[ref22] Wood A. J. L.; Weise N. J.; Frampton J. D.; Dunstan M. S.; Hollas M. A.; Derrington S. R.; Lloyd R. C.; Quaglia D.; Parmeggiani F.; Leys D.; Turner N. J.; Flitshc S. L. Adenylation Activity of Carboxylic Acid Reductases Enables the Synthesis of Amides. Angew. Chem., Int. Ed. 2017, 56, 14498–14501. 10.1002/anie.201707918. 28940631

[ref23] Lubberink M.; Schnepel C.; Citoler J.; Derrington S. R.; Finnigan W.; Hayes M. A.; Turner N. J.; Flitsch S. L. Biocatalytic Monoacylation of Symmetrical Diamines and Its Application to the Synthesis of Pharmaceutically Relevant Amides. ACS Catal. 2020, 10, 10005–10009. 10.1021/acscatal.0c02228.

[ref24] Schnepel C.; Pérez L. R.; Yu Y.; Angelastro A.; Heath R. S.; Lubberink M.; Falcioni F.; Mulholland K.; Hayes M. A.; Turner N. J.; Flitsch S. L. Thioester-mediated biocatalytic amide bond synthesis with in situ thiol recycling. Nat. Catal. 2023, 6, 89–99. 10.1038/s41929-022-00889-x.

[ref25] Gulick A. M. Conformational Dynamics in the Acyl-CoA Synthetases, Adenylation Domains of Non-ribosomal Peptide Synthetases, and Firefly Luciferase. ACS Chem. Biol. 2009, 4, 811–827. 10.1021/cb900156h. 19610673 PMC2769252

[ref26] Steffensky M.; Li S. M.; Heide L. Cloning, Overexpression, and Purification of Novobiocic Acid Synthetase from Streptomyces spheroides NCIMB 11891. J. Biol. Chem. 2000, 275, 21754–21760. 10.1074/jbc.M003066200. 10801869

[ref27] Schmutz E.; Steffensky M.; Schmidt J.; Porzel A.; Li S. M.; Heide L. An Unusual Amide Synthetase (CouL) from the Coumermycin A1 Biosynthetic Gene cluster from Streptomyces rishiriensis DSM 40489. Eur. J. Biochem. 2003, 270, 4413–4419. 10.1046/j.1432-1033.2003.03830.x. 14622269

[ref28] Galm U.; Dessoy M. A.; Schmidt J.; Wessjohann L. A.; Heide L. In Vitro and in Vivo Production of New Aminocoumarins by a Combined Biochemical, Genetic, and Synthetic Approach. Chem. Biol. 2004, 11, 173–183. 10.1016/j.chembiol.2004.01.012. 15123279

[ref29] Luft T.; Li S. M.; Scheible H.; Kammerer B.; Heide L. Overexpression, Purification and Characterization of SimL, an Amide Synthetase Involved in Simocyclinone Biosynthesis. Arch. Microbiol. 2005, 183, 277–285. 10.1007/s00203-005-0770-0. 15812631

[ref30] Pacholec M.; Meyers C. L. F.; Oberthür M.; Kahne D.; Walsh C. T. Characterization of the Aminocoumarin Ligase SimL from the Simocyclinone Pathway and Tandem Incubation with NovM,P,N from the Novobiocin Pathway. Biochemistry 2005, 44, 4949–4956. 10.1021/bi047303g. 15779922

[ref31] Rangaswamy V.; Ullrich M.; Jones W.; Mitchell R.; Parry R.; Reynolds P.; Bender C. L. Expression and Analysis of Coronafacate Ligase, a Thermoregulated Gene Required for Production of the Phytotoxin Coronatine in Pseudomonas syringae. FEMS Microbiol. Lett. 2006, 154, 65–72. 10.1111/j.1574-6968.1997.tb12625.x. 9297822

[ref32] Winn M.; Rowlinson M.; Wang F.; Bering L.; Francis D.; Levy C.; Micklefield J. Discovery, Characterization and Engineering of Ligases for Amide Synthesis. Nature 2021, 593, 391–398. 10.1038/s41586-021-03447-w. 34012085

[ref33] Ji C.; Chen Q.; Li Q.; Huang H.; Song Y.; Ma J.; Ju J. Chemoenzymatic Synthesis of β-Carboline Derivatives Using McbA, a New ATP-dependent Amide Synthetase. Tetrahedron Lett. 2014, 55, 4901–4904. 10.1016/j.tetlet.2014.07.004.

[ref34] Petchey M.; Cuetos A.; Rowlinson B.; Dannevald S.; Frese A.; Sutton P. W.; Lovelock S.; Lloyd R. C.; Fairlamb I. J. S.; Grogan G. The Broad Aryl Acid Specificity of the Amide Bond Synthetase McbA Suggests Potential for the Biocatalytic Synthesis of Amides. Angew. Chem., Int. Ed. 2018, 57, 11584–11588. 10.1002/anie.201804592. PMC6282839 30035356

[ref35] Wu R.; Cao J.; Lu X.; Reger A. S.; Gulick A. M.; Dunaway-Mariano D. Mechanism of 4-Chlorobenzoate:Coenzyme A Ligase Catalysis. Biochemistry 2008, 47, 8026–8039. 10.1021/bi800698m. 18620421 PMC3694354

[ref36] Reger A. S.; Wu R.; Dunaway-Mariano D.; Gulick A. M. Structural Characterization of a 140° Domain Movement in the Two-Step Reaction Catalyzed by 4-Chlorobenzoate:CoA Ligase. Biochemistry 2008, 47, 8016–8025. 10.1021/bi800696y. 18620418 PMC2666193

[ref37] Petchey M. R.; Rowlinson B.; Lloyd R. C.; Fairlamb I. J. S.; Grogan G. Biocatalytic Synthesis of Moclobemide Using the Amide Bond Synthetase McbA Coupled with an ATP Recycling System. ACS Catal. 2020, 10, 4659–4663. 10.1021/acscatal.0c00929. 32337091 PMC7171872

[ref38] Zhu Y.; Zhang Q.; Fang C.; Zhang Y.; Ma L.; Liu Z.; Zhai S.; Peng J.; Zhang L.; Zhu W.; Zhang C. Refactoring the Concise Biosynthetic Pathway of Cyanogramide Unveils Spirooxindole Formation Catalyzed by a P450 Enzyme. Angew. Chem., Int. Ed. 2020, 59, 14065–14069. 10.1002/anie.202004978. 32329169

[ref39] Lee R. A.; Razaz M.; Hayward S. The DynDom Database of Protein Domain Motions. Bioinformatics 2003, 19, 1290–1291. 10.1093/bioinformatics/btg137. 12835274

[ref40] Waterhouse A.; Bertoni M.; Bienert S.; Studer G.; Tauriello G.; Gumienny R.; Heer F. T.; de Beer T. A. P.; Rempfer C.; Bordoli L.; et al. SWISS-MODEL: Homology Modelling of Protein Structures and Complexes. Nucleic Acids Res. 2018, 46, W296–W303. 10.1093/nar/gky427. 29788355 PMC6030848

[ref41] Chen C.-S.; Fujimoto Y.; Girdaukas G.; Sih C. J. Quantitative Analyses of Biochemical Kinetic Resolutions of Enantiomers. J. Am. Chem. Soc. 1982, 104, 7294–7299. 10.1021/ja00389a064.

[ref42] Trott O.; Olson A. J. AutoDock Vina: Improving the Speed and Accuracy of Docking with a New Scoring Function, Efficient Optimization, and Multithreading. J. Comput. Chem. 2010, 31, 455–461. 10.1002/jcc.21334. 19499576 PMC3041641

