# Supplementary material for: Broad Spectrum Enantioselective Amide Bond Synthetase from Streptoalloteichus hindustanus
Source: ACS Catal. 2024 Jan 6;14(2):1021–9. doi: 10.1021/acscatal.3c05656 (PMC10804368; doi:10.1021/acscatal.3c05656)
Supplement: Supplementary file 1 — cs3c05656_si_001.pdf [file cs3c05656_si_001.pdf]

A Broad Spectrum Enantioselective Amide Bond Synthetase from *Streptoalloteichus hindustanus*

Qingyun Tang,[a] Mark Petchey,[q] Benjamin Rowlinson,[a] Thomas J. Burden,[a] Ian J.S. Fairlamb,[a] and Gideon Grogan[a*]*

[a]Department of Chemistry, University of York, Heslington, York YO10 5DD U.K.

*Corresponding author e-mails: ian.fairlamb@york.ac.uk; gideon.grogan@york.ac.uk

Supporting Information

Table of Contents

Section 1. Amino Acid Sequences and Phylogenetic Analysis………..………………3

Section 2. Cloning, Expression and Purification of *Sh*ABS and mutants………..……9

Section 3. Synthesis of Amide Standards………………………………………………13

Section 4. X-Ray Crystallography and Molecular Docking……………………………15

Section 5. Small-scale Biotransformations……………………….…………………..19

Section 6. Scaled-up Biotransformations…………………… …………………………21

Section 7. RP-HPLC and chiral HPLC analysis…………………………………………22

Section 8. Chiral LC and LC-MS Chromatograms……………………………………...26

Section 9. Compound characterisation and NMR spectra……………………………..29

Section 10. References………………………………………………………………...66

Section 1. Amino Acid Sequences and Phylogenetic Analysis

1.1 Amino acid and DNA Sequence of ShABS and other ABSs

Streptalloteichus hindustanus amide bond synthetase (S*h*ABS)

- GenBank accession number: WP_073481158.1
- Extinction coefficient (280 nm): 65235 $M^{-1}cm^{-1}$
- Molecular weight: 54.382 kDa

```
MGSSHHHHHHSSGLVPRGSHMGYLHRVVEGLKANAGGEALVSADRRLTGAETLEEIHRTARA
LAAQGLRPGDGVVTLHGNGVEAVVLRIAVQLLGCRYAGLRPVFATREKANFLAEAEAAAFVY
QPDMADEAAELLREVPTPRVLSLGPAPLGEDLVALAGAQSAEPVEFTADERAATAVGFTGGT
TGRAKGVCRAPFDLEACLDASLTIFGEGPWRFLVCIPIADLGGEMAEWTLAAGGTVVLREDF
EPADILATIGAERTTHVFCAPGWVYQLAEHPALADADLSSLTQIPYGGAPSTPARIADALEK
LGRPLLVHCYGSQEGGWMTWLSAEDHVRADRYLLNSVGKALPGTEIAIRDQDGADLPVGTVG
EVCVRSTMLMRGYWRLPELTAKTVRDGWLHTGDLGRLDTEGYLYLVDRAKDVIIVEAYNVYS
QEVEHVLTGHPDVRYAAVVGVPDHDTTEAVYAAVVPAEGVGEIDVDELRALVRTTLGPVHEP
KHLDVVDTIPTTPRGKPDKSALRTRWRAAQRA
```

Figure S1. Amino acid sequence of *Sh*ABS with hexahistidine tag (grey) in pET28a(+).

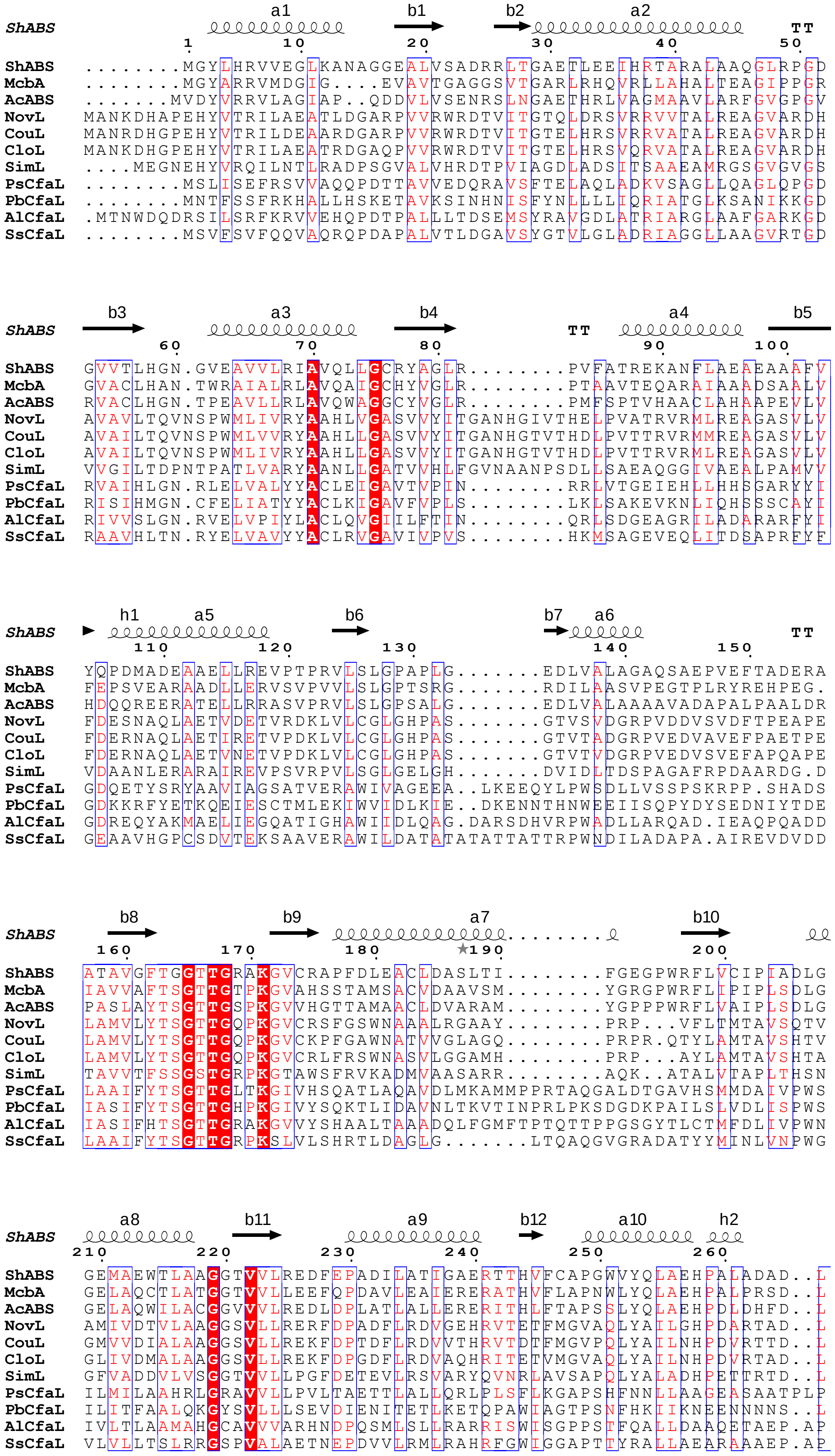
ShABS a1 b1 b2 a2 TT
1 10 20 30 40 50
ShABS ........MGYLHRVVEGLKANAGGEALVSADRRLTGAETLEEIHRTARALAAQGLRPGD
McbA ........MGYARRVMDGIG....EVAVTGAGGSVTGARLRHQVRLLAHALTEAGIPPGR
AcABS .......MVDYVRRVLAGIAP..QDDVLVSENRSLNGAETHRLVAGMAAVLARFGVGPGV
NovL MANKDHAPEHYVTRILAEATLDGARPVVRWRDTVITGTQLDRSVRRVVTALREAGVARDH
CouL MANRDHGPEHYVTRILDEAARDGARPVVRWRDTVITGTELHRSVRRVATALREAGVARDH
CloL MANKDHGPEHYVTRILAEATRDGAQPVVRWRDTVITGTELHRSVQRVATALREAGVARDH
SimL ....MEGNEHYVRQILNTLRADPSGVALVHRDTPVIAGDLADSITSAAEAMRGSGVGVGS
PsCfaL ........MSLISEFRSVVAQQPDTTAVVEDQRAVSFTELAQLADKVSAGLLQAGLQPGD
PbCfaL ........MNTFSSFRKHALLHSKETAVKSINHNISFYNLLLLIQRIATGLKSANIKKGD
AlCfaL .MTNWDQDRSILSRFKRVVEHQPDTPALLLTDSEMSYRAVGDLATRIARGLAAFGARKGD
SsCfaL ........MSVFSVFQQVAQRQPDAPALVTLDGAVSYGTVLGLADRIAGGLLAAGVRTGD
ShABS b3 a3 b4 TT a4 b5
60 70 80 90 100
ShABS GVVTLHGN.GVEAVVLRIAVQLLGCRYAGLR........PVFATREKANFLAEAEAAAFV
McbA GVACLHAN.TWRAIALRLAVQAIGCHYVGLR........PTAAVTEQARAIAAADSAALV
AcABS RVACLHGN.TPEAVLLRLAVQWAGGCYVGLR........PMFSPTVHAACLAHAAPEVLV
NovL AVAVLTQVNSPWMLIVRYAAHLVGASVVYITGANHGIVTHELPVATRVRMLREAGASVLV
CouL AVAILTQVNSPWMLVVRYAAHLLGASVVYITGANHGTVTHDLPVTTRVRMMREAGASVLV
CloL AVAILTQVNSPWMLIVRYAAHLLGASVVYITGANHGTVTHDLPVTTRVRMLREAGASVLV
SimL VVGILTDPNTPATLVARYAANLLGATVVHLFGVNAANPSDLLSAEAQGGIVAEALPAMVV
PsCfaL RVAIHLGN.RLELVALYYACLEIGAVTVPIN........RRLVTGEIEHLLHHSGARYYI
PbCfaL RISIHMGN.CFELIATYYACLKIGAVFVPLS........LKLSAKEVKNLIQHSSSCAYI
AlCfaL RIVVSLGN.RVELVPIYLACLQVGIILFTIN........QRLSDGEAGRILADARARFYI
SsCfaL RAAVHLTN.RYELVAVYYACLRVGAVIVPVS........HKMSAGEVEQLITDSAPRFYF
ShABS h1 a5 b6 b7 a6 TT
110 120 130 140 150
ShABS YQPDMADEAAELLREVPTPRVLSLGPAPLG.......EDLVALAGAQSAEPVEFTADERA
McbA FEPSVEARAADLLERVSVPVVLSLGPTSRG.......RDILAASVPEGTPLRYREHPEG.
AcABS HDQQREERATELLRRASVPRVLSLGPSALG.......EDLVALAAAAVADAPALPAALDR
NovL FDESNAQLAETVDETVRDKLVLCGLGHPAS.......GTVSVDGRPVDDVSVDFTPEAPE
CouL FDERNAQLAETIRETVPDKLVLCGLGHPAS.......GTVTADGRPVEDVAVEFPAETPE
CloL FDERNAQLAETVNETVPDKLVLCGLGHPAS.......GTVTVDGRPVEDVSVEFAPQAPE
SimL VDAANLERARAIREVPSVRPVLSGLGELGH.......DVIDLTDSPAGAFRPDAARDG.D
PsCfaL GDQETYSRYAAVIAGSATVERAWIVAGEEA..LKEEQYLPWSDLLVSSPSKRPP.SHADS
PbCfaL GDKKRFYETKQEIESCTMLEKIWVIDLKIE..DKENNTHNWEEIISQPYDYSEDNIYTDE
AlCfaL GDREQYAKMAELIEGQATIGHAWIIDLQAG.DARSDHVRPWADLLARQAD.IEAQPQADD
SsCfaL GEAAVHGPCSDVTEKSAAVERAWILDATATATATTATTRPWNDILADAPA.AIREVDVDD
ShABS b8 b9 a7 b10
160 170 180 ★190 200
ShABS ATAVGFTGGTTGRAKGVCRAPFDLEACLDASLTI........FGEGPWRFLVCIPIADLG
McbA IAVVAFTSGTTGTPKGVAHSSTAMSACVDAAVSM........YGRGPWRFLIPIPLSDLG
AcABS PASLAYTSGTTGSPKGVVHGTTAMAACLDVARAM........YGPPPWRFLVAIPLSDLG
NovL LAMVLYTSGTTGQPKGVCRSFGSWNAAALRGAAY........PRP...VFLTMTAVSQTV
CouL LAMVLYTSGTTGQPKGVCKPFGAWNATVVGLAGQ........PRPR.QTYLAMTAVSHTV
CloL LAMVLYTSGTTGQPKGVCRLFRSWNASVLGGAMH........PRP...AYLAMTAVSHTA
SimL TAVVTFSSGSTGRPKGTAWSFRVKADMVAASARR........AQK..ATALVTAPLTHSN
PsCfaL LAAIFYTSGTTGLTKGIVHSQATLAQAVDLMKAMMPPRTAQGALDTGAVHSMMDAIVPWS
PbCfaL IASIFYTSGTTGHPKGIVYSQKTLIDAVNLTKVTINPRLPKSDGDKPAILSLVDLISPWS
AlCfaL IASIFHTSGTTGRAKGVVYSHAALTAAADQLFGMFTPTQTTPPGSGYTLCTMFDLIVPWN
SsCfaL LAAIFYTSGTTGRPKSLVLSHRTLDAGLG.......LTQAQGVGRADATYYMINLVNPWG
ShABS a8 b11 a9 b12 a10 h2
210 220 230 240 250 260
ShABS GEMAEWTLAAGGTVVLREDFEPADILATIGAERTTHVFCAPGWVYQLAEHPALADAD..L
McbA GELAQCTLATGGTVVLLEEFQPDAVLEAIERERATHVFLAPNWLYQLAEHPALPRSD..L
AcABS GELAQWILACGGVVVLREDLDPLATLALLERERITHLFTAPSSLYQLAEHPDLDHFD..L
NovL AMIVDTVLAAGGSVLLRERFDPADFLRDVGEHRVTETFMGVAQLYAILGHPDARTAD..L
CouL GMVVDIALAAGGSVLLREKFDPTDFLRDVVTHRVTDTFMGVPQLYAILNHPDVRTTD..L
CloL GLIVDMALAAGGSVLLREKFDPGDFLRDVAQHRITETVMGVAQLYAILNHPDVRTAD..L
SimL GFVADDVLVSGGTVVLLPGFDETEVLRSVARYQVNRLAVSAPQLYALADHPETTRTD..L
PsCfaL ILMILAAHRLGRAVVLLPVLTAETTLALLQRLPLSFLKGAPSHFNNLLAAGEASAATPLP
PbCfaL ILITFAALQKGYSVLLLSEVDIENITETLKETQPAWIAGTPSNFHKIIKNEENNNS..L
AlCfaL IVLTLAAMAHGCAVVVARHNDPQSMLSLLRARRISWISGPPSTFQALLDAAQETAEP.AP
SsCfaL VLVLLTSLRRGSPVALAETNEPDVVLRMLRAHRFGWIGGAPTTYRALLAEARAAAEP.AP

```
ShABS        TT   b13        a11              b14  TTT  b15    h3   TT    TT
               270       280       290       300  *    310       320
ShABS   SSLTQIPYGGAPSTPARIADALEKLGRPLLVHCYGSQEGGWMTWLSAEDHVRADRYLLNS
McbA    SSLRRVVYGGAPAVPSRVAAARERMG.AVLMQNYGTQEAAFIAALTPDDHA..RRELLTA
AcABS   TALRLAVYGGAPAVPARTSAALRRLG.PRLMQNYGTQETGFVCALLPEDHG..HPELLDH
NovL    SSLRHVLYLGCPASPERLREAAALLP.GVLAQSYGSTEAGRITVLRAADHE..RPELLAT
CouL    SSLQHLVYVGCPASPERLREAVTVFP.GVLWQSYGSTETGRIAMLREDDHD..HPELLAT
CloL    SSLRHLLYLGCPASPERLQEAATVLP.GVLAQSYGSTEAGRITVLREADHE..RPELLAT
SimL    SSVRDLFYTGVAASPERVAVAEKVFG.SVLMQVYGTSETNIISWLIAGEHT..DAGLRAT
PsCfaL  SLTSTYSVSGGDPCPPKLGRRWHNLWGGTLRGSYGTTESG.PIFCQPDVAA....TEQSS
PbCfaL  DLSETVCVAGGDSCATELSQKFFECFGSHLQSSYGQTELGGPVIYHHDIYA....INEPS
AlCfaL  DLSATNCVAGGDACSLELSRQFLAKFGSRMQSCYGQTEIG.PVLFQPDLMA....AEVPS
SsCfaL  DLTDTRCVTGGDACPIELSREFAETFKSSLTGVYGMTETAAPVIEQPRIDA....VDEPS

        b16     b17               b18        b19    a12    b20      b21
ShABS     TT             TT      TT        TT              TT
          330       340       350       360       370       380
ShABS   VGKALPGTEIAIRD.QDGADLPVGTVGEVCVRSTMLMRGYWRLPELTAKTVRDGWLHTGD
McbA    VGRPLPHVEVEIRD.DSGGTLPRGAVGEVWVRSPMTMSGYWRDPERTAQVLSGGWLRTGD
AcABS   AGRLLPGVEVEIRD.TSGAVVPPGVVGEIWVRSPMTMSGYHADPARTGEVLVDGWVRTGD
NovL    VGRAVPGVTIAIRDPETGHDLPVGEIGEVVVHGPEVMAGYVADPEHTARVIRDGWVHTGD
CouL    VGRPMPGVTIAIRDPQTGRDLPVNEIGEVVVHSPNNMAGYIGDPEGTTRVVRDGWVHTGD
CloL    VGQAMPGVTIAIRDPETGRDLPVNQIGEVVVHSPEAMGGYVADPEHTARVVRDGWVHTGD
SimL    VGRPLEWLRVTIRDPQDERVLPTGETGEVWVNSPWRMDHYWNDPEQTARTVRDGWIRTGD
PsCfaL  IGWPLPGVALQQT...........ENGELLIRSPANTPGLWNGQD.ADRLPAARWIATGD
PbCfaL  IGWPLPGVEIKINN.......TQSSNGELLIRSPAKTIGIWNGHD.IELFPSDRWLATGD
AlCfaL  IGWPMPGVQVRIDA.......PDGEPGPLLLRGPSLAVGLWKDGE.IERFDPDRWLATED
SsCfaL  IGWPLPGVEIRVDG.......EPDEAGELMIRTPSRPVGSWNGTG.VDPFDRSQWLASGD

                          b22    h4  b23  b24      a13          b25          b26
ShABS            TT                    TT                            TTT
           390       400       410       420       430       440
ShABS   LGRLDTEGYLYLVDRAKDVIIVEAYNVYSQEVEHVLTGHPDVRYAAVVGVPDHDTTEAVY
McbA    VGTFDEDGHLHLTDRLQDIIIVEAYNVYSRRVEHVLTEHPDVRAAAVVGVPDPDSGEAVC
AcABS   LGHLSEADLLRILDRAKDLIIVEAYNVYSRRVEDVLCHHPKVLQAGVVGLPDVDRGERVC
NovL    FGSVDERGYVRLFGRMREVVKVQDTRVSPTEVEKVLVGCPGVVDACVYGHRGPDLIEELH
CouL    MGSVDERGYVRLFGRMHEMVKVQDTRVSPTEVEKVLVGCPGVVDACVYGHRRPDLIEELH
CloL    FGSVDERGYVRLFGRMHEMVKVQDTRVSPTEVEKVLVGCPGVVDACVYGHRRSDLIEELH
SimL    VGHLDDAGYLHLHGRLAGVIKTNGIKVYPVAVERSLLDHPDVAEAAVFGVENSDRVERIH
PsCfaL  LVQRQDDGGYLIIGREKDMLKCDAYSISPVEVEQELLKLPDIAEAVVFGVPDATIGERPV
PbCfaL  LVRQENNRNLIFLGREKDQIKIEGYPVYPIEIENTLIQHADIAASVVFSVPDKYAGERII
AlCfaL  VVRQRPDGCLLFLGRTQDLIKIGGYATSPLELEQHIGRHPDVADVVVFSVPEPKLGATIV
SsCfaL  IVRRRPDGCLLFVGRKKDLIMVEGYPIAPLEIERELSAHPDVAAAIAFGVPDDATGERVV

                          a14       h5     b27                a15
ShABS             TT
             450       460       470       480       490       500
ShABS   AAVVPAEGVGEIDVDELRALVRTTLGPVHEPKHLDVVDTIPTTPRGKPDKSALRTRWRAA
McbA    AAVVVADGAD.PDPEHLRALVRDHLGDLHVPRRVEFVRSIPVTPAGKPDKVKVRTWFTD.
AcABS   AAVVLASGT..ADADELREHVEAQLGEPHVPRLIRFVDTIPLTDGGKPDKSALRALFAPD
NovL    AAVVLGTEGA.PSFDTLRDHVARAMTPTHAPIRFVRWRRFPINNTGKVNRLRVREVSAEA
CouL    AAVVLSTEDA.PSFAALRDHVAQTMTPTHAPVRFVRWRQFPINNTGKTDRLRIREVSAEA
CloL    AAVVLSTDGA.PSFAALRDHVAQAMTPTHAPIRFVRWRQFPINNTGKVDRLRIREVSAEA
SimL    AVVVLREGAG.AGPEDLRQHVSSHLSPNHAPADIELRSSLPLIGFGKPDKLRLRADAMAR
PsCfaL  ALLRTTSGRE.LPTQQLKQHLKALIAEYKHPRQYLFVERIPLSSAGKVSRKQLASDYREI
PbCfaL  ALIQPQKNHS.LKAETIASYLSDNLAHYKHPSEYIFIKEIPVNTTGKISRRKLSNEYHLL
AlCfaL  AMIQPVDGKQ.VTCEELSVFLTGTIAQYKHPRAVVNVEQIPVMGNGKLGRKRLSDDYIAA
SsCfaL  ALVEPEPGRD.IDPAKLLEHLTGRIGAYKHPSEIIPVDRLPVLPTGKLGRQRLTADYLAA

ShABS

ShABS   QRA...........
McbA    ..............
AcABS   TPSR..........
NovL    RGDSPDVLVDR...
CouL    RGEGPDVLVDR...
CloL    RGESPDVLVDR...
SimL    REATHGE.......
PsCfaL  LGAAPST.......
PbCfaL  KSQAEKVFTL....
AlCfaL  RSNR..........
SsCfaL  REDASRAAAEESAS
```

Figure S2. Sequence alignment of *Sh*ABS, McbA and other ABSs was performed on ClustalW software (http://www.genome.jp/tools-bin/clustalw)[1] and visualised by ESPript 3.0 (http://espript.ibcp.fr/ESPript/cgi-bin/ESPript.cgi),[2] where letters coloured in white with red background and letters coloured in red indicate fully conserved residues and conserved residues of similar properties between groups, respectively. Secondary structure of *Sh*ABS in the 'adenylation' conformation (PDB code: 8PPP) is shown above the alignment. ABSs used for sequence alignment: *Sh*ABS, ABS from *Streptoalloteichus hindustanus* (WP_073481158.1); McbA, ABS from *Marinactinospora thermotolerans* (WP_144390195.1); *Ac*ABS, ABS from *Actinoalloteichus cyanogriseus* (WP_051713392.1); NovL, ABS from *Streptomyces niveus* (WP_069626142.1); CouL, ABS from *Streptomyces rishiriensis* (WP_109005073.1); CloL, ABS from *Streptomyces roseochromogenus* (WP_023545109.1); SimL, ABS from *Streptomyces antibioticus* (AAG34183.1); *Ps*CfaL, ABS from *Pseudomonas syringae* (WP_005894383.1); *Pb*CfaL, ABS from *Pectobacterium brasiliense* (WP_039313124.1); *Al*CfaL, ABS from *Azospirillum lipoferum* (WP_012975623.1); *Ss*CfaL, ABS from *Streptomyces scabies* (WP_013005377.1).

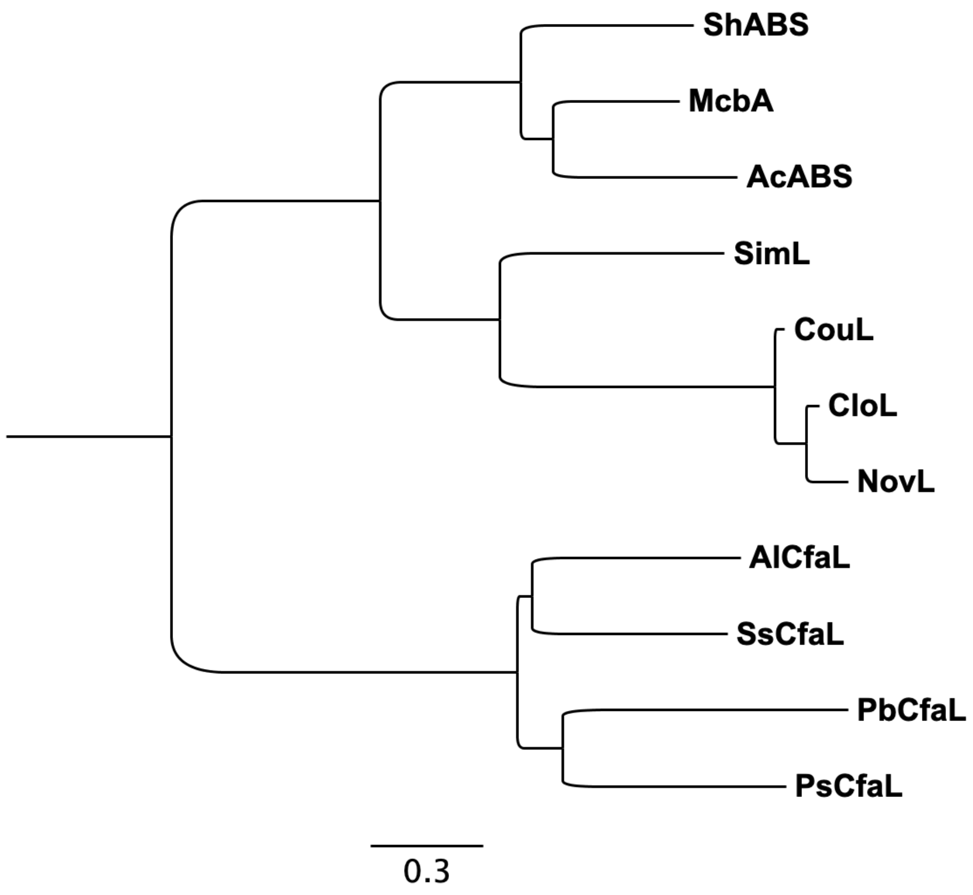

Figure S3. Phylogenetic tree of selected ABSs. The tree was constructed based on the multiple sequence alignment results in Figure S2, using FastTree v2.1.8 with default parameters[3] and was rooted to midpoint with the FigTree software (http://tree.bio.ed.ac.uk/software/figtree/) to ensure a better comparability. The numbers on the branches denote the number of substitutions per site.

ATGGGCAGCAGCCATCATCATCATCATCACAGCAGCGGCCTGGTGCCGCGCGGCAGCCATAT
GGGTTACCTGCACCGTGTGGTTGAAGGTCTGAAGGCGAACGCGGGTGGCGAAGCGCTGGTTA
GCGCGGACCGTCGTCTGACCGGTGCGGAGACCCTGGAGGAAATCCACCGTACCGCGCGTGCG
CTGGCGGCGCAGGGTCTGCGTCCGGGTGATGGCGTGGTTACCCTGCATGGTAACGGTGTGGA
GGCGGTGGTTCTGCGTATTGCGGTTCAACTGCTGGGTTGCCGTTATGCGGGTCTGCGTCCGG
TGTTTGCGACCCGTGAAAAAGCGAACTTTCTGGCGGAGGCGGAAGCGGCGGCGTTCGTTTAT
CAGCCGGACATGGCGGATGAAGCGGCGGAACTGCTGCGTGAGGTGCCGACCCCGCGTGTTCT
GAGCCTGGGTCCGGCGCCGCTGGGTGAAGACCTGGTTGCGCTGGCGGGTGCGCAAAGCGCGG
AGCCGGTTGAATTCACCGCGGATGAACGTGCGGCGACCGCGGTGGGTTTTACCGGTGGCACC
ACCGGTCGTGCGAAGGGCGTTTGCCGTGCGCCGTTTGACCTGGAGGCGTGCCTGGATGCGAG
CCTGACCATCTTCGGTGAAGGCCCGTGGCGTTTTCTGGTGTGCATCCCGATTGCGGACCTGG
GTGGCGAGATGGCGGAATGGACCCTGGCGGCGGGTGGCACCGTGGTTCTGCGTGAGGACTTC
GAACCGGCGGATATCCTGGCGACCATCGGTGCGGAGCGTACCACCCATGTGTTTTGCGCGCC
GGGTTGGGTTTACCAACTGGCGGAACATCCGGCGCTGGCGGATGCGGATCTGAGCAGCCTGA
CCCAAATTCCGTATGGTGGCGCGCCGAGCACCCCGGCGCGTATTGCGGATGCGCTGGAGAAG
CTGGGTCGTCCGCTGCTGGTTCACTGCTACGGCAGCCAGGAAGGTGGTTGGATGACCTGGCT
GAGCGCGGAAGACCATGTGCGTGCGGATCGTTATCTGCTGAACAGCGTTGGTAAAGCGCTGC
CGGGCACCGAAATCGCGATTCGTGACCAAGATGGTGCGGACCTGCCGGTGGGTACCGTTGGC
GAGGTGTGCGTTCGTAGCACCATGCTGATGCGTGGTTATTGGCGTCTGCCGGAACTGACCGC
GAAGACCGTGCGTGATGGTTGGCTGCACACCGGTGACCTGGGCCGTCTGGATACCGAGGGCT
ACCTGTATCTGGTGGACCGTGCGAAAGATGTGATCATTGTTGAAGCGTACAACGTTTATAGC
CAGGAAGTGGAACACGTTCTGACCGGTCATCCGGATGTGCGTTATGCGGCGGTGGTTGGCGT
TCCGGACCACGATACCACCGAGGCGGTTTATGCGGCGGTGGTTCCGGCGGAGGGTGTGGGTG
AAATCGACGTTGATGAACTGCGTGCGCTGGTGCGTACCACCCTGGGTCCGGTTCACGAACCG
AAACACCTGGACGTGGTTGATACCATTCCGACCACCCCGCGTGGCAAGCCGGATAAAAGCGC
GCTGCGTACCCGTTGGCGTGCGGCGCAACGTGCG*

Figure S4. Synthetic gene encoding *Sh*ABS, codon optimised for expression in *E. coli.* *N*-terminal hexahistidine tag and thrombin cleavage site was labelled in grey.

Section 2. Cloning, Expression and Purification of *Sh*ABS and mutants

2.1 General

Chemically competent cells were purchased from Merck and NEB. Q5 Site-Directed Mutagenesis Kit and DpnI restriction enzyme were supplied from New England Biolabs (NEB). In-Fusion Snap Assembly Kit was purchased from Takara Bio. LB medium (high salt Miller's broth) and TB (Terrific Broth) medium were purchased from Melford. Oligonucleotides were synthesised by Merck Life Science Limited or Integrated DNA Technologies.

2.2 Gene Cloning and Expression

Synthetic gene of *Sh*ABS in pET-28a(+) with *N*-terminal hexahistidine tag was purchased from GeneArt (Invitrogen). Codons were optimised for *E. coli* expression. The recombinant pET28a(+)-*Sh*ABS transformed into *E. coli* BL21 (DE3) using 30 μg mL^{-1} kanamycin as the antibiotic marker on Luria-Bertani (LB) agar. A single colony grown overnight at 37 °C was then used to inoculate 5 mL of LB broth containing 30 μg mL^{-1} kanamycin, which was then grown for 18 h at 37°C with shaking at 180 rpm. These starter cultures were then each used to inoculate 1 L LB broth containing 30 μg mL^{-1} kanamycin and these cultures grown until the optical density (OD 600) had reached 0.6. Expression of the *Sh*ABS gene was then induced by the addition of 1 mM isopropyl β-D-1-thiogalactopyranoside (IPTG), after which the culture was incubated at 16°C in an orbital shaker at 180 rpm for approximately 18 h. Cells were then harvested by centrifugation for 20 min at 5,422 *g*.

2.2. Purification of ShABS

The cell pellet from 1 L growth was resuspended in 50 mL 50 mM HEPES buffer (pH 7.5) containing 300 mM NaCl, 10 mM $MgCl_2$, 1 % glycerol (v/ v) and 20 mM imidazole ('binding buffer' henceforth). The cells were lysed at 27 MPa in a cell disruptor and the lysate pelleted by centrifugation for 30 min at 28,928 *g* in a Lynx6000 centrifuge. The supernatants were then loaded onto a 5 mL His-Trap™ Chelating HP nickel column. After washing with 10 column volumes of binding buffer, *Sh*ABS was eluted with a gradient of 20–300 mM imidazole over 20 column volumes. Column fractions containing the protein, as determined by SDS-PAGE analysis (**Figure S5a**), were

pooled and then concentrated using a 30 kDa cut-off Centricon® filter membrane. The concentrated fractions were then loaded onto a pre-equilibrated S75 Superdex™ 16/600 gel filtration column and eluted with buffer lacking imidazole, at a flow rate of 0.8 mL min^{-1}. Fractions containing pure *Sh*ABSs, as determined by SDS-PAGE analysis (**Figure S5a**), were pooled, flash cooled in 500 µL aliquots in liquid nitrogen, and stored at -80 °C. All mutants were purified with same methods and subjected to SDS-PAGE analysis (**Figure S5b** and **S5c**).

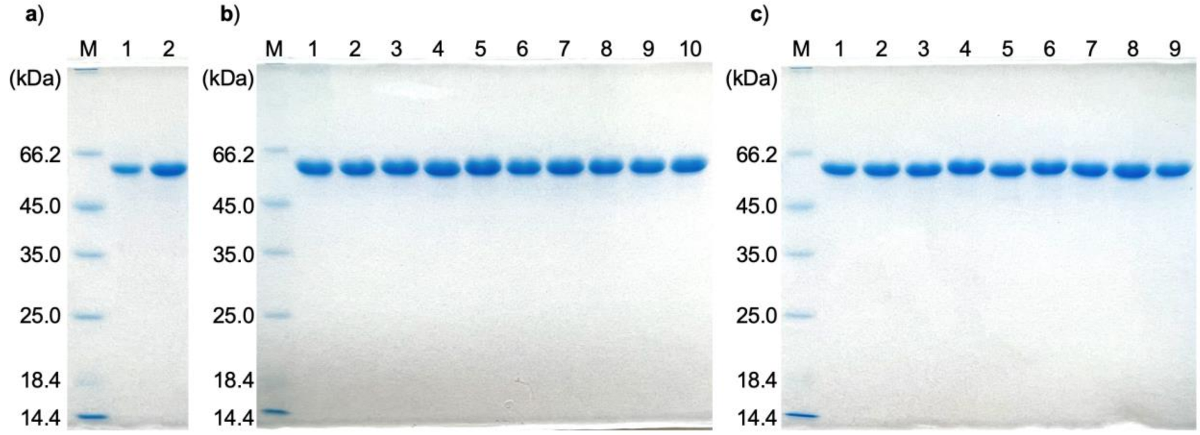

Figure S5. SDS-PAGE analysis of *Sh*ABS and mutants. **a**) Purification of wild-type *Sh*ABS with His-Trap™ Chelating HP nickel column (lane 1) and S75 Superdex™ 16/600 gel filtration column (lane 2). M, protein molecular weight marker; **b**) Purified mutants R175A, C183A, L207A, E210A, W214A, F246A, C299A, S302A, Q303A, W307A (lane 1-10) within the acid substrate binding pocket; **c**) Purified mutants V84A, I202A, P203A, W251A, E408A, Y410A, V473A (lane 1-7) within the amine substrate binding pocket and mutants K492A, K492H (lane 8-9) for protein crystallisation.

For protein crystallisation, the gene of *Sh*ABS was subcloned into a modified pET28a vector pET-YSBLIC-3C with a human rhinovirus (HRV) 3C protease cleavage site between the *N*-terminal hexahistidine tag and the gene which has been proved to assist protein crystallisation.[4] The pET-YSBLIC-3C vector was cloned with the primers 5'-GGCGCGCCTTCTCCTCAC-3' (forward) and 5'-TGCTGGTCCCTGGAACAGAAC-3' (reverse); The gene of *Sh*ABS was cloned with the primers 5'-TTCCAGGGACCAGCAATGGGTTACCTGCACCGTGTGG-3' (forward) and 5'-AGGAGAAGGCGCGCCTTACGCACGTTGCGCCGC-3' (reverse) before being ligated using the In-Fusion Snap Assembly Kit (Takara Bio). After confirming the gene

with sequencing, the recombinant pET-YSBLIC-3C-S*h*ABS was expressed in *E. coli* Bl21 (DE3) and purified with a His-Trap nickel column. The *N*-terminal His_6-tag was removed by the HRV 3C protease while being dialyzed at 4 °C into buffer overnight (50 mM HEPES, pH 7.5, 1 mM DTT) using dialysis membranes (12,000-14,000 MWCO). The resultant reaction mixtures were loaded onto His-Trap nickel column again and the flow-through was collected and concentrated for gel filtration. Protein concentrations were determined by measuring absorbance at 280 nm using NanoPhotometer (NP80, IMPLEN) and the molecular extinction coefficient given in **Figure S1.**

2.3. Site-directed mutagenesis of ShABS

Site-directed mutagenesis of S*h*ABS was performed using the Q5 Site-Directed Mutagenesis Kit following the instruction. Primers were designed on NEBaseChanger (nebasechanger.neb.com) (Table S2). PCRs contained 1 x Q5 Hot Start High-Fidelity Master Mix, 0.5 µM each of forward and reverse primer and 0.2 ng μL^{-1} of template DNA (pET-28a(+)-S*h*ABS). After initial denaturation for 30 s at 98°C, 25 cycles of denaturation at 98°C for 10 s, annealing at 64-68°C for 20 s, and extension at 72°C for 3.50 min were performed, followed by a final extension at 72°C for 7 min. PCR products were treated with Kinase, Ligase & DpnI (KLD) from the kit, transformed to the Stellar Competent Cells (Merck), plated on LB Miller agar containing 30 $\mu g\ mL^{-1}$ kanamycin and incubated overnight at 37 °C. Plasmid DNA of the mutants were isolated (Monarch Plasmid Miniprep Kit, NEB) and sent for Sanger sequencing (Eurofins Genomics).

Table S2. Primers for mutagenesis of S*h*ABS. Mutated sites were underlined.

Mutant	Forward Primer 5' → 3'	Reverse Primer 5' → 3'
Mutants in acid substrate binding pocket		
R175A	GGGCGTTTGCGCGGCGCCGTTTGACC	TTCGCACGACCGGTGGTG
C183A	CCTGGAGGCGGCGCTGGATGCGAGCC	TCAAACGGCGCACGGCAA
L207A	TCCCGATTGCGGACGCGGGTGGCGAGAT	TGCACACCAGAAAACGCCACGGGCCTTCAC
E210A	CCTGGGTGGCGCGATGGCGGAAT	TCCGCAATCGGGATGCACAC

W214A	GATGGCGGAAGCGACCCTGGCGGCG	TCGCCACCCAGGTCCGCA
F246A	CACCCATGTGGCGTGCGCGCCGGGTT	GTACGCTCCGCACCGATGGTCGCCA
C299A	GCTGGTTCACGCGTACGGCAGCCAGGAAG	AGCGGACGACCCAGCTTC
S302A	CTGCTACGGCGCGCAGGAAGGTGGTTGG	TGAACCAGCAGCGGACGA
Q303A	CTACGGCAGCGCGGAAGGTGGTTG	CAGTGAACCAGCAGCGGA
W307A	GGAAGGTGGTGCGATGACCTGGCTG	TGGCTGCCGTAGCAGTGA
Mutants in amine substrate binding pocket		
V84A	TCTGCGTCCGGCGTTTGCGACCC	CCCGCATAACGGCAACCC
I202A	TCTGGTGTGCGCGCCGATTGCGGACC	AAACGCCACGGGCCTTCA
P203A	GGTGTGCATCGCGATTGCGGACC	AGAAAACGCCACGGGCCTTC
W251A	CGCGCCGGGTGCGGTTTACCAAC	CAAAACACATGGGTGGTACG
E408A	GATCATTGTTGCGGCGTACAACGTTTATAG	ACATCTTTCGCACGGTCC
Y410A	TGTTGAAGCGGCGAACGTTTATAGCCAG	ATGATCACATCTTTCGCAC
V473A	CCTGGGTCCGGCGCACGAACCGAAAC	GTGGTACGCACCAGCGCA
Mutants for crystallisation		
K492A	CCCGCGTGGCGCGCCGGATAAAA	GTGGTCGGAATGGTATCAAC
K492H	CCCGCGTGGCCATCCGGATAAAA	GTGGTCGGAATGGTATCAAC

Section 3. Amide Product Standard Synthesis

3.1. General

All chemicals were of high analytical grade and purchased from Sigma-Aldrich, Alfa Aesar, Acros Organics or Fluorochem and were at least 95% pure. HPLC solvents were purchased from VWR Chemicals and were of HPLC grade.

3.2. Amide synthesis

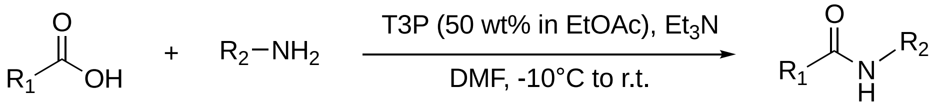

Scheme S1

The β-carboline acids **5-9** were synthesised according to procedures detailed previously.[5,6] Amide product standards were synthesised using T3P® (propanephosphonic acid anhydride) as coupling reagent (**Scheme S1**).[5,6] For compounds **10a-17a** and **19m**, acid **10-17** or **19** (0.82 mmol, 1.0 equiv.), amine **a** or **m** (155 µL or 121.4 µL; 1.23 mmol, 1.5 equiv.), Et_3N (570 µL, 4.1 mmol, 5.0 equiv.), were added to dry DMF (2 mL). For compounds **5a-5m**, acid **5** (81 mg, 0.32 mmol, 1.0 equiv.), amine **a-m** (0.48 mmol, 1.5 equiv.) and Et_3N (223 µL, 1.6 mmol, 5.0 equiv.) were used. The reaction mixture was cooled down to -10 °C and T3P® solution (50 wt% in EtOAc, 381 µL, 0.64 mmol, 2.0 equiv.) was added carefully. The solution was stirred at -10 °C for 10 min and then 1 h at room temperature. The reaction was then quenched by adding 10 mL water and stirred for 5 min. 10 mL of 0.2 N HCl was then added and the reaction was extracted with EtOAc (3 x 20 mL). The organic phases were collected and washed with 0.2 N HCl, 0.2 N NaOH and brine, dried over anhydrous Na_2SO_4, filtered and evaporated under reduced pressure. The crude reaction product was then purified by flash chromatography to obtain the final amide products.

3.3. Compound characterisation

Thin layer chromatography (TLC) was carried out using Merck 5554 aluminium backed silica plates (silica gel 60 F254) and spots were visualized using UV light (at 254 nm). Retention factors (R_f) are reported along with the solvent system used, in parentheses.

Flash column chromatography was performed according to the method reported by Still and co-workers[7] using Merck 60 silica gel (particle size 40–63 μm) and the solvent systems for TLC.

NMR spectra were obtained in the solvent indicated, using a JEOL ECX400 or JEOL ECS400 spectrometer (400MHz and 101 MHz for ^{1}H and ^{13}C, respectively), a Bruker 500 (500 MHz and 126 MHz for ^{1}H and ^{13}C, respectively) or a Bruker 600 (600 MHz and 151 MHz for ^{1}H and ^{13}C, respectively). Chemical shifts are reported in parts per million and were referenced to the residual non-deuterated solvent of the deuterated solvent used ($CHCl_3$ TMH = 7.26 and TMC = 77.16 ($CDCl_3$), (CHD_2)SO(CD_3) TMH = 2.50 and TMC = 39.52 {SO(CD_3)$_2$}, ^{1}H, ^{13}C, respectively). Spectra were typically run at a temperature of 298 K. All ^{13}C NMR spectra were obtained with ^{1}H decoupling. NMR spectra were processed using MestreNova software (Version 14.2.1-27684, released May 2021). Compound structures are shown with arbitrary numbering indicated by ChemDraw (Version 20.0.0.38). For the ^{1}H NMR spectra the resolution varies from 0.15 to 0.5 Hz; the coupling constants have been quoted to ± 0.5 Hz in all cases for consistency. ^{1}H and ^{13}C NMR chemical shifts are quoted to 2 decimal places.

MS spectra were measured using a Bruker Daltonics micrOTOF MS, Agilent series 1200LC with electrospray ionisation (ESI and APCI) or on a Thermo LCQ using electrospray ionisation, with <5 ppm error recorded for all HRMS samples. Mass spectral data is quoted as the *m/z* and mass to charge ratios (*m/z*) are reported in Daltons.

Infrared spectra were obtained using a Bruker ALPHA-Platinum FTIR Spectrometer with a platinum-diamond ATR sampling module.

Melting points were recorded using a Stuart digital SMP3 machine.

Section 4. X-ray Crystallography and Molecular Docking

4.1. X-ray Crystallography

Wild-type S*h*ABS and mutants K492A/H were expressed and purified as above and N-terminal His_6-tags were removed as described. Enzymes in 50 mM HEPES pH 7.5, 300 mM NaCl, 10 mM $MgCl_2$ and 1% glycerol after gel filtration were concentrated to 77 mg mL^{-1} (1.42 mM). 2 molar equivalents of ligand (α,β-methylene ATP, carboxylic acid **5** or amide **10a** in 100 mM stock solution) was added to the protein and incubated on ice for 30 min. Proteins were then subjected to crystallization screening using the commercial screening plates CSS I&II, PACT (Molecular Dimensions) and Index (Hampton research) in a 96-well sitting drop format using a Mosquito robot (TTP Labtech), in which 150 nL of protein solution was mixed with 150 nL of precipitant solution. Crystal trays were placed at 4 ˚C for crystallisation.

Three datasets from *Sh*ABS crystals are presented: Dataset #1 is a complex of *Sh*ABS K492H in complex with AMP-CPP refined to 2.57 Å resolution, obtained with precipitant containing 0.2 M $LiSO_4$, 0.1 M HEPES, pH 7.5, 25% PEG 3350; Dataset #2 is again derived from K492H in complex with AMP-CPP obtained with the same precipitants as dataset#1, but is of significantly higher resolution at 2.02 Å. In this complex only density for the adenosine part of AMP-CPP could be modelled. Dataset #3 comes from crystals of WT *Sh*ABS pre-complexed with amide **10a** with precipitant 1.8 M Li_2SO_4, 0.1 M Bis-tris propane, pH 6.5, and was refined to 2.90 Å, but no electron density consistent with the amide was was observed. All crystals were harvested directly into liquid nitrogen from the crystallisation drop and stored for data collection.

Datasets #1, #2 and #3 were collected at the Diamond Light Source, Didcot, Oxfordshire, U.K. on beamlines I03, I03 and I04 respectively. Data were processed and integrated using XDS[8] and scaled using SCALA[9] included in the Xia2 processing system.[10] Data collection statistics are provided in **Table S3**. Crystals for Datasets #1, #2 and #3 were obtained in space groups $P2_1$, $P2_1$ and $P6_32_2$ respectively, with two, two and one molecules in the asymmetric unit in each case; The structures were solved by molecular replacement using MOLREP[11] with monomer 'A' of the $McbA_{Ad}$ from *Marinactinospora thermotolerans* (PDB code 6SQ8) as the initial model. The structures were then built and refined using iterative cycles in Coot[12] and REFMAC,[13]

employing local NCS restraints in the refinement cycles, where relevant. For dataset #1, following building and refinement of the protein and water molecules in this complex, residual density was observed in the omit maps at the active sites of each monomer, which could be clearly modelled as AMP-CPP. For Dataset #2, residual omit density was also observed, but only the atoms for adenosine could be modelled reliably. The final structures for Datasets #1, #2 and #3 exhibited % R_{cryst} /R_{free} values of 21.2/27.6 and 22.7/26.3 and 17.5/21.4 respectively Refinement statistics for the structures are presented in **Table S3.** The coordinates and structure factor files have been deposited in the Protein Databank (PDB) with accession codes **8PPP**, **8PYX** and **8PYY** respectively.

Table S3. Data collection and refinement statistics for *Sh*ABS datasets. Numbers in brackets refer to data for highest resolution shells.

	$ShABS_{Ad}$ K492H in complex with AMP-CPP **Dataset #1** **8PPP**	$ShABS_{Ad}$ K492H in complex with adenosine **Dataset #2** **8PYX**	$ShABS_{open}$ wild-type **Dataset #3** **8PYY**
Beamline	I03	I03	I04
Wavelength (Å)	0.95881	0.95881	0.95374
Resolution (Å)	58.12-2.57 (2.68-2.57)	29.33-2.02 (2.06-2.02)	57.26-2.90 (3.08-2.90)
Space Group	$P2_1$	$P2_1$	$P6_32_2$
Unit cell (Å)	a = 82.70; b = 95.70; c = 87.20 $\alpha = \gamma = 90.00°$; β = 117.80°	a = 82.17; b = 96.16; c = 87.32 $\alpha = \gamma = 90.00°$; β = 117.74°	a = b = 161.53; c = 162.38 $\alpha = \beta = 90.00°$; γ = 120.00°
No. of molecules in the asymmetric unit	2	2	1
Unique reflections	38357 (4648)	78528 (3766)	28308 (4468)
Completeness (%)	99.8 (99.9)	99.0 (83.2)	100.0 (100.0)
R_{merge} (%)	0.17 (1.11)	0.12 (1.05)	0.34 (1.93)
$R_{p.i.m.}$	0.10 (0.67)	0.06 (0.55)	0.05 (0.30)
Multiplicity	6.9 (7.1)	7.0 (6.4)	40.1 (40.2)
$<I/\sigma(I)>$	8.5 (1.7)	11.6 (1.7)	11.3 (1.5)
Overall *B* from Wilson plot ($Å_2$)	36	26	48
$CC_{1/2}$	1.00 (0.83)	1.00 (0.80)	1.00 (0.83)
R_{cryst}/ R_{free} (%)	21.2/27.6	22.7/26.3	17.5/21.4
r.m.s.d 1-2 bonds (Å)	0.007	0.007	0.007
r.m.s.d 1-3 angles (°)	1.477	1.398	1.598
Avge main chain B ($Å^2$)	39	31	51
Avge side chain B ($Å^2$)	43	35	57
Avge waters B ($Å^2$)	31	35	40
Avge Ligand B ($Å^2$)	80	57	-

4.2. Molecular docking

The S*h*ABS$_{am}$ model is constructed on the SWISS-MODEL[14] online server with default parameters using the McbA$_{Am}$ (PDB: 6sq8, Chain E) structure as model to obtain the desired conformation. Docking of the chiral amide products (*R*)-**12a** and (*R*)-**5c** to the S*h*ABS$_{am}$ model was performed on AutoDock Vina.[15] The grid box was set to 80, 82, and 64 Å for X, Y, and Z, respectively, and was centred to both the acid and amine substrate binding pockets; the exhaustiveness was set to 20. Docking results with the highest binding energy were selected and visualised in PyMOL.[16]

Section 5. Small-scale Biotransformations

Acid and amine substrate stock solutions were prepared in DMSO to a final concentration of 100 mM. ATP was dissolved in Milli-Q water to a final concentration of 100 mM. Inorganic pyrophosphatase (IPase, Merck, cat. no.: I1643) was prepared to a final concentration of 100 U mL^{-1} in Milli-Q water. Small-scale biotransformation screening reactions were performed on 200 µL scale with 0.4 mM acid substrate, 0.6 mM amine substrate (1.5 eq.), 0.8 mM ATP (2 eq.) and 2 U mL^{-1} IPase in 50 mM sodium phosphate buffer, pH 7.5. Reactions were initiated by the addition of ABS enzyme to a final concentration of 1.0 mg mL^{-1}. The reactions were then placed in an incubating shaker at 37°C and 800 rpm. 100 µL sample was taken at 1 h and 24 h, respectively, and reactions were stopped with the addition of 200 µL MeCN (0.1% (v/v) formic acid). Samples were then centrifuged at 16,300 *g* for 10 min and transferred to vials for RP-HPLC analysis. Calibration curves of acid substrates and amidation products were plotted with HPLC peak areas versus concentrations ranging from 0-0.5 mM. All reactions and analysis were performed in duplicate.

For the analysis of *Sh*ABS enantioselectivity, racemic acids or amines were used as substrates. *Sh*ABS was added to a final concentration of 0.1 or 1.0 mg mL^{-1} and reaction times were varied from 4 to 24h to avoid complete consumption of both substrate enantiomers. 100 µL sample was mixed with 200 µL MeCN (0.1% v/v formic acid) for conversion analysis; another 100 µL sample was extracted with 100 µL EtOAc twice and transferred to vials for chiral HPLC analysis. Chiral amide standards were prepared using the corresponding chiral substrates. Enantiomeric excess (*ee*) values were calculated with HPLC peak area of each enantiomer. All reactions were performed in duplicate.

In the initial pharmaceutical amide target screening, 1 mM acid substrate, 1 mM (1.0 equiv.) amine substrate, 1.5 mM ATP (1.5 equiv.), 2 U mL^{-1} IPase and 1.0 mg mL^{-1} S*h*ABS were added to 50 mM NaP_i buffer, pH 7.5 and incubated at 37°C and 800 rpm. 100 µL sample was taken at 16 h, mixed with 200 µL MeCN (0.1% (v/v) formic acid), centrifuged at 16,300 *g* for 10 min and then subjected to LC-MS analysis (**Figure S6**). After confirming amide products with authentic standards, small-scale biotransformation of **18l**, **19m**, **11n** and **20o** were performed with 5 mM acid substrate,

10 mM (2.0 eq.) amine substrate, 10 mM ATP (2 eq.) and 2.0 mg mL^{-1} *Sh*ABS at 37°C and 800 rpm. Amine substrates were supplied with the same amount at 8, 24 and 32h for each reaction. 20 µL samples were taken at 8, 24, 32 and 48h, mixed with 80 µL reaction buffer and 200 µL MeCN (0.1% v/v formic acid) before HPLC analysis. Calibration curves of acid substrates and amide products were plotted with HPLC peak areas versus concentrations ranging from 0-1.0 mM. Conversions over time were shown in **Figure S7**. All reactions and analysis were performed in duplicate.

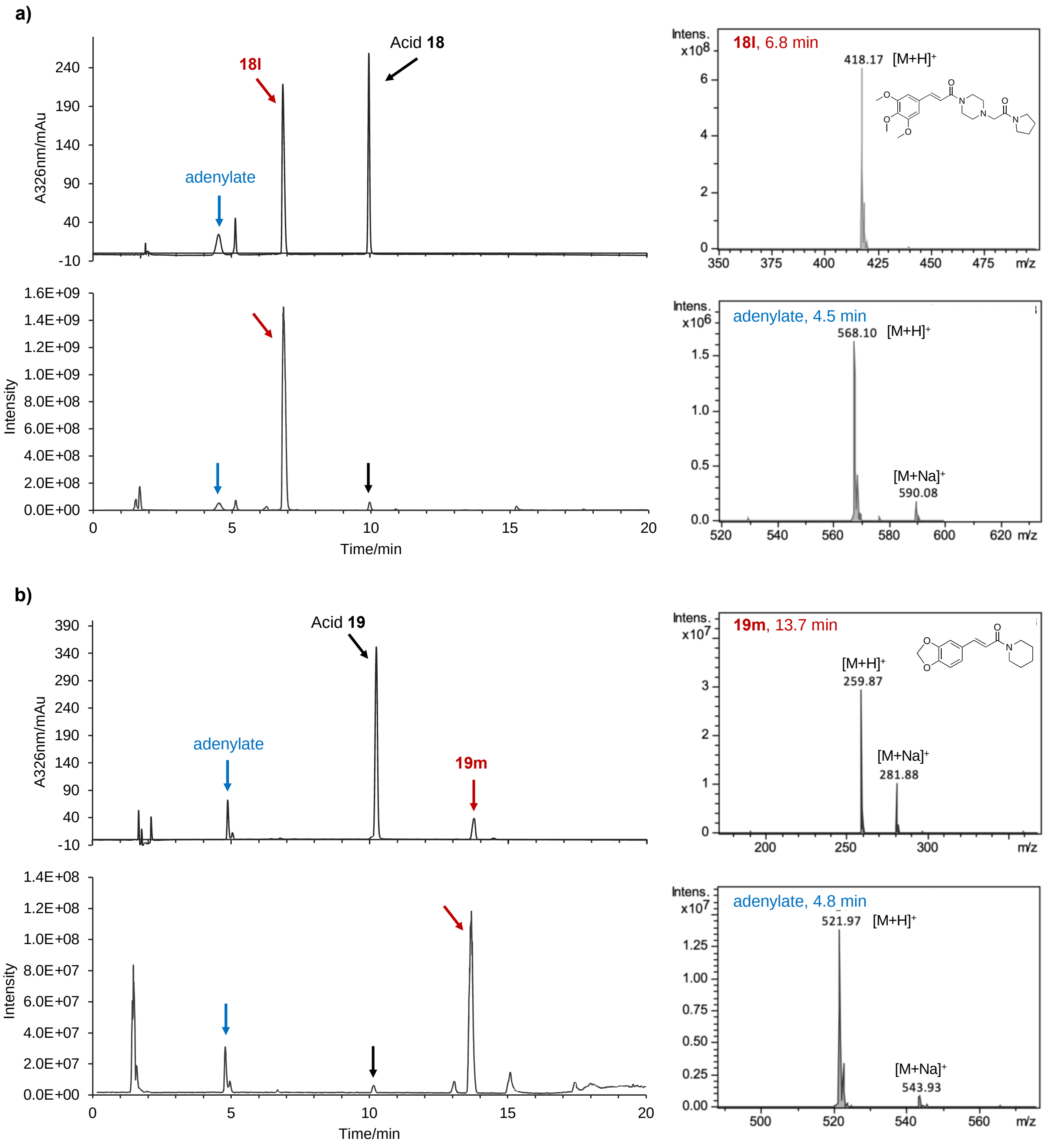

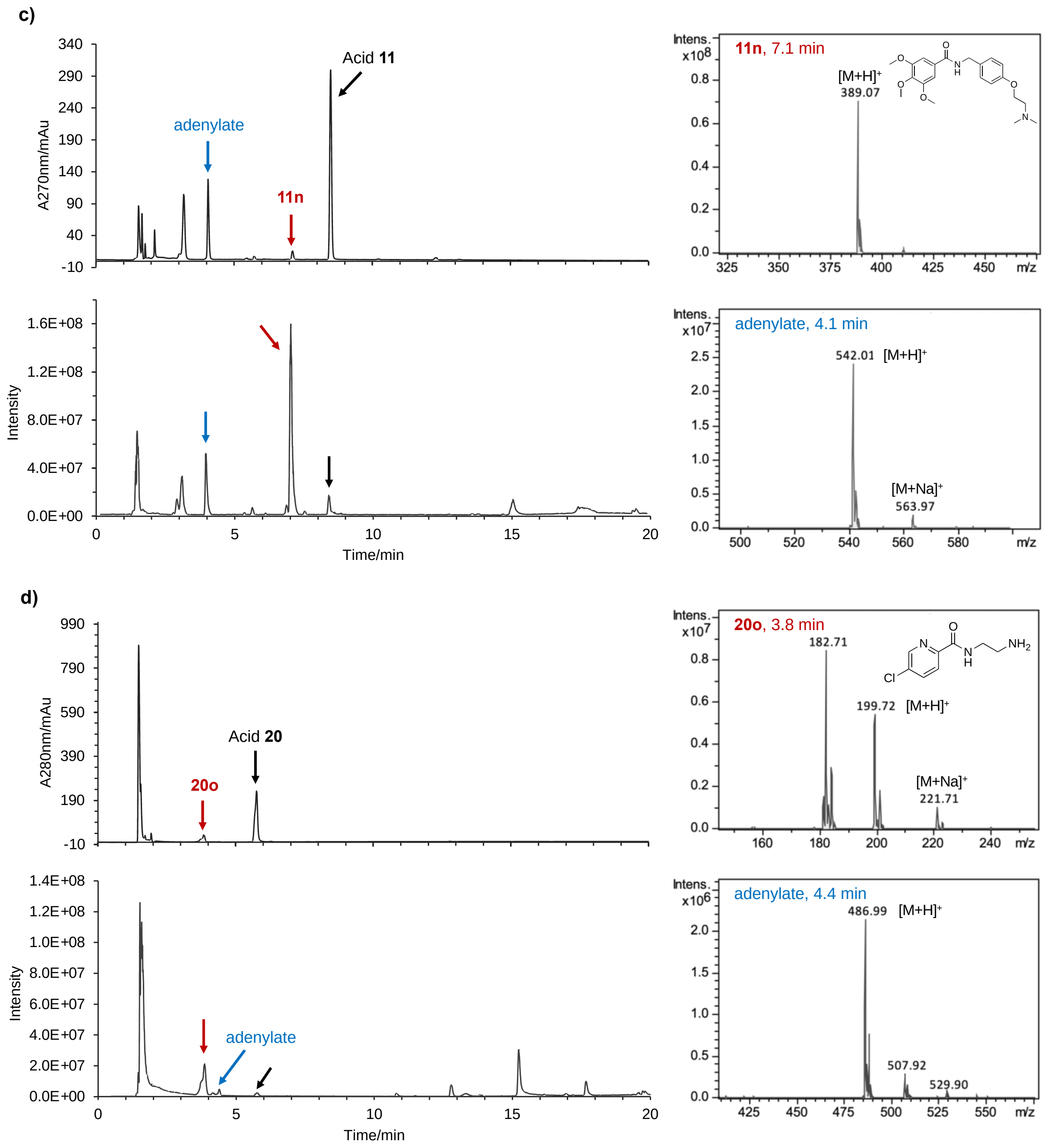

Figure S6. LC-MS chromatograms in the synthesis of pharmaceutical amides a) **18l**, b) **19m**, c) **11n** and d) **20o** catalyzed by *Sh*ABS (top: UV; bottom: MS). MS spectra of both amide products and acid adenylates were shown.

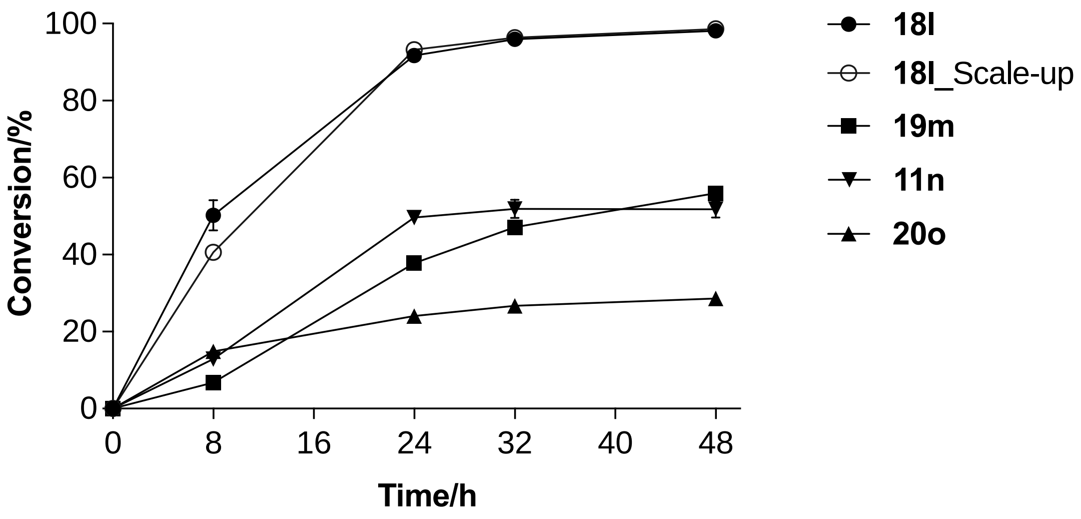

Figure S7. Time course conversion of pharmaceutical amides **18l**, **19m**, **11n** and **20o** and the scale-up biotransformation of **18l**. Amine substrates were supplied at 8, 24 and 32h.

Section 6. Scale-up Biotransformations

Scale-up biotransformation of **18l** was performed in a 50 mg (acid substrate) scale. Before the reaction, 50 mg of acid **18** (0.21 mmol) and 82.8 mg of amine **l** (0.42 mmol, 2 eq.) were dissolved in 500 µL DMSO, respectively; 231.3 mg ATP (0.42 mmol, 2 equiv.) was dissolved in 1 mL Mili-Q. All reaction components were added to 50 mM sodium phosphate buffer, pH 7.5 in a 100 mL Schott bottle to give 5 mM acid **18**, 10 mM amine **l**, 10 mM ATP, 0.5 U mL^{-1} IPase. The reaction was initiated by adding purified S*h*ABS to the final concentration of 2.0 mg mL^{-1} and incubated at 37°C, 160 rpm. The amine substrate was supplied with the same amount at 8, 24 and 32h. After 48h, the reaction has reached 99% conversion (**Figure S7**) and the amide product was extracted with DCM (3 x 30 mL) after the reaction mixture was adjusted to pH 11-12 with K_2CO_3. The organic phases were collected and the solvent was evaporated under reduced pressure. The resulting mixture was then purified with preparative HPLC (Shimadzu LC-20) equipped with UV detection and the SunFire C18 OBD Prep Column, 100Å, 5 µm, 19 mm X 150 mm (Waters). The amide product was eluted with Methanol:Water (60:40, v/v) with column temperature at 30°C and flow rate at 10 mL/min. After purification, the mobile phase was evaporated under reduced pressure and elevated temperature (60°C). It resulted 83.1 mg (0.20 mmol) of the desired product **18l** (99% conversion, 95% yield) as a light yellow liquid. The ^{1}H and ^{13}C NMR spectra are shown in **Figure S8a** and **S8b**, respectively, which match the ones reported in literature.[17]

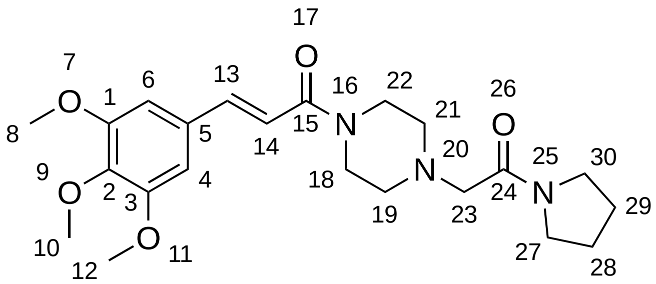

18l

^{1}H NMR ($CDCl_3$, 600 MHz) δ 7.55 (1H, d, *J* = 15.3 Hz, H-13), 6.83 – 6.60 (3H, m, H-4, H-6, H-14, overlapped), 3.87 (6H, s, H-8, H-12), 3.85 (3H, s, H-10), 3.82 – 3.63 (4H, m, H-18, H-22), 3.46 (4H, dt, *J* = 14.0, 6.9 Hz, H-27, H-30), 3.17 (2H, s, H-23), 2.73 – 2.55 (4H, m, H-19, H-21), 1.95 (2H, p, *J* = 6.8 Hz, H-28 or H-29), 1.84 (2H, p, *J* = 6.8 Hz, H-28 or H-29). **^{13}C NMR ($CDCl_3$, 151 MHz)** δ 167.41 (C-24), 165.47 (C-15), 153.50 (C-1, C-3), 143.01 (C-13), 139.72 (C-2), 130.93 (C-5), 116.39 (C-14), 105.13 (C-4, C-

6), 61.05 (C-10), 60.67 (C-23), 56.33 (C-8, C-12), 53.24 (C-19, C-21), 46.29 (C-27, C-30), 45.96 (C-18, C-22), 26.29 (C-28 or C-29), 24.17 (C-28 or C-29).

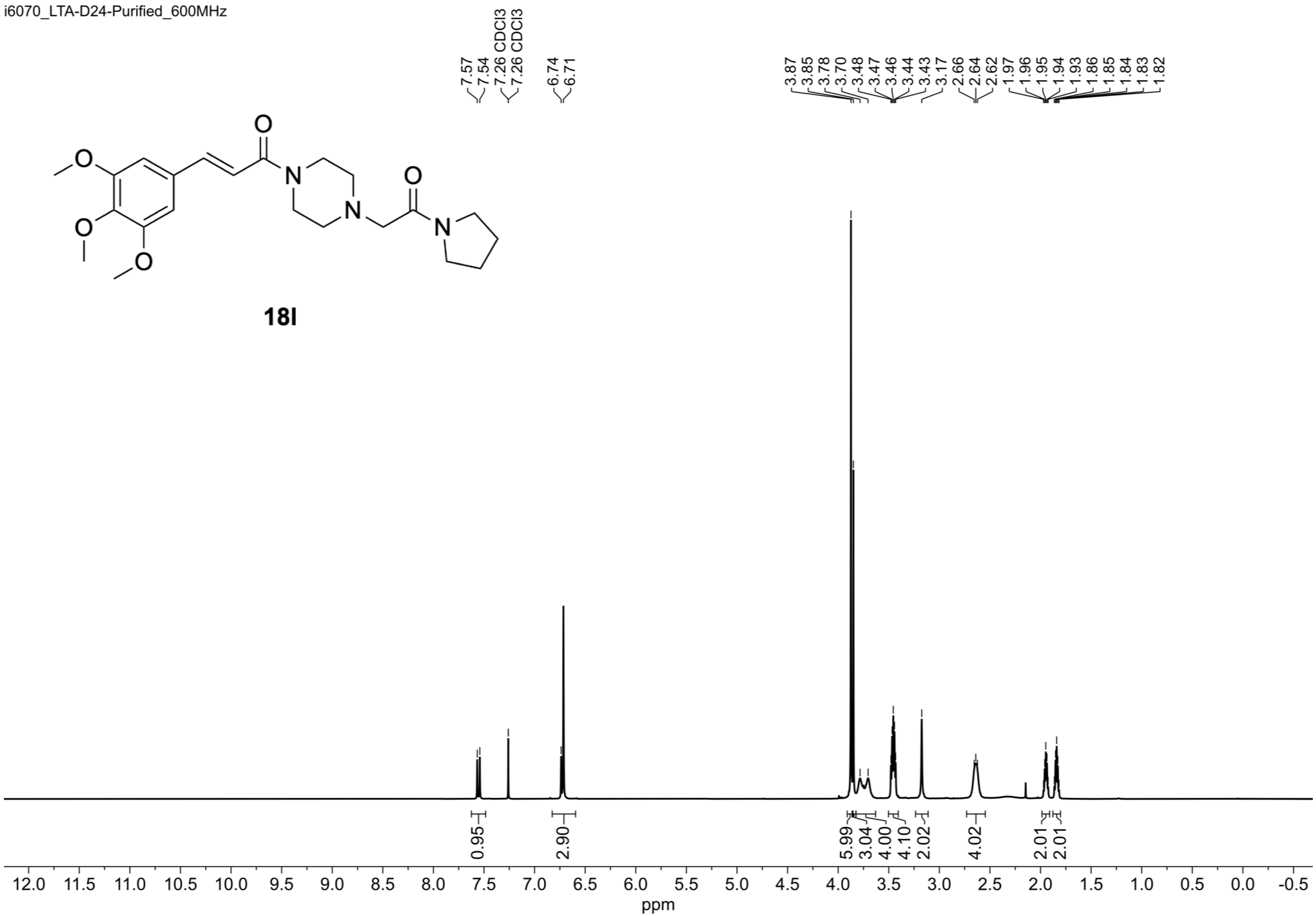

Figure S8a. ^{1}H NMR spectrum of compound **18l** from the scale-up biotransformation (**$CDCl_3$, 600 MHz**).

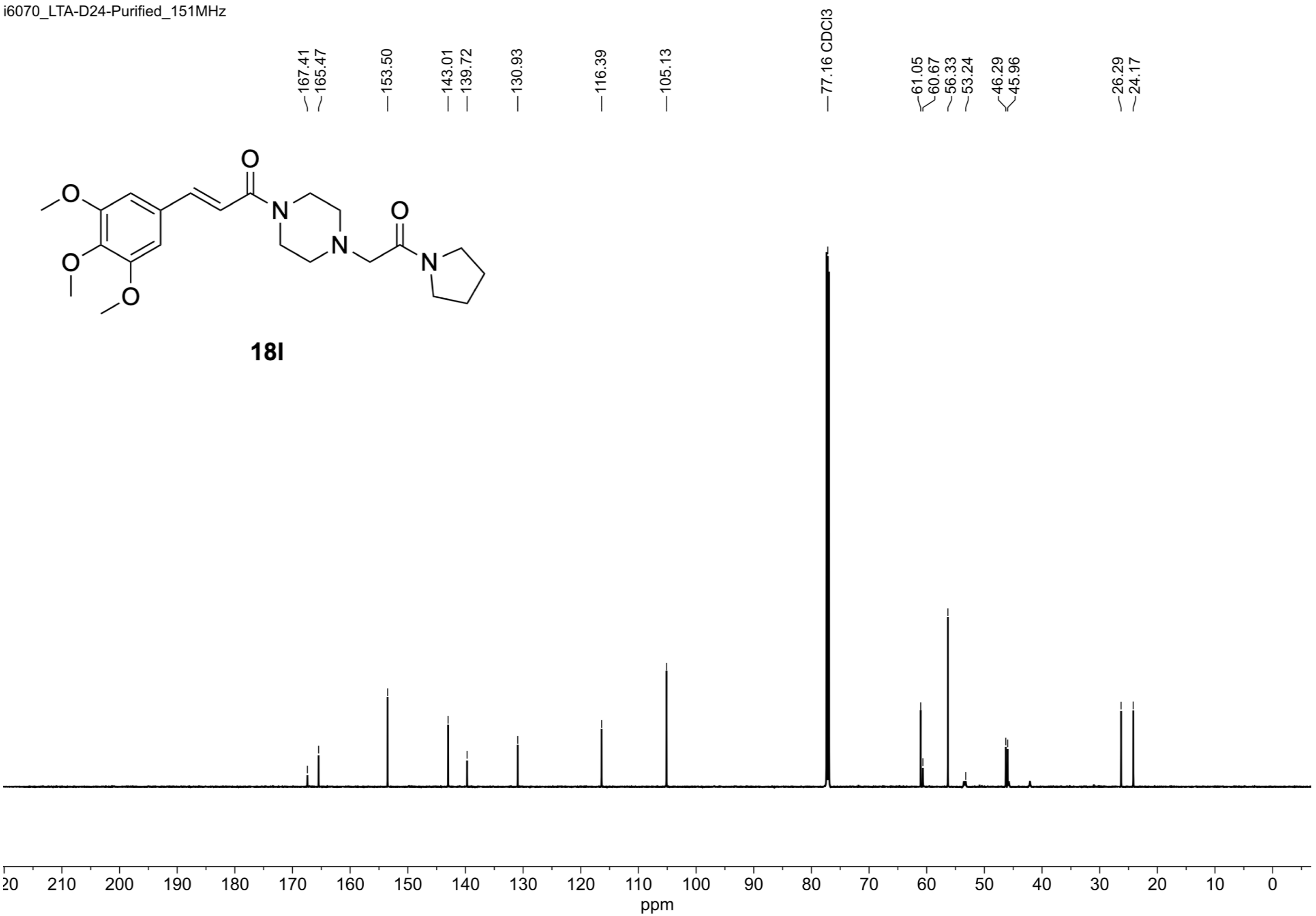

Figure S8b. ^{13}C NMR spectrum of compound **18l** from the scale-up biotransformation (**$CDCl_3$, 151 MHz**).

Section 7. RP-HPLC and chiral HPLC analysis

7.1 Reverse-phase (RP) HPLC

Biotransformations were analysed on the Agilent 1260 Infinity II LC System, with diode array UV detector, fitted with an Agilent Poroshell 120 EC-C18 column, 2,7 µm, 4,6 x 150 mm. The eluents employed were: A) 0.1% v/v solution of formic acid in water; B) 0.1% v/v solution of formic acid in acetonitrile.

Method I: 10% to 80% B in 10 min, hold for 2 min; 80% to 10% B in 0.2 min, hold for 5 min for re-equilibration. 35 °C, flow 1.0 mL min^{-1}.

Method II: 30% to 80% B in 7 min, hold for 2 min; 80% to 30% B in 0.2 min, hold for 5 min for re-equilibration. 35 °C, flow 1.0 mL min^{-1}.

Retention times and calibration curves for acid substrates and amidation products are listed in **Table S4**.

Table S4. Retention times and calibration curves for substrates and products.X: compound concentration (mM); Y: peak area (mAU).

Compounds	Method	Retention times/min	Wavelength/nm	Calibration curves
Acid substrate screening				
**10/10a**	I	4.3/7.3	230	Y = 2159*X - 2.043/<br>Y = 1967*X + 13.37
**11/11a**	I	4.5/7.3	260	Y = 1769*X - 1.616/<br>Y = 1877*X + 9.661
**12/12a**	I	3.0/6.6	218	Y = 1161*X + 2.594/<br>Y = 1574*X + 11.54
**13/13a**	I	5.6/8.2	208	Y = 1412*X - 2.613<br>Y = 3619*X + 112.2
**14/14a**	I	9.2/10.9	218	Y = 1513*X + 8.432<br>Y = 2306*X + 5.679
**15/15a**	I	5.7/8.1	280	Y = 3858*X + 21.34<br>Y = 4286*X + 50.55

**16/16a**	I	6.8/8.9	230	Y = 8808*X + 69.32<br>Y = 9831*X + 26.64
**17/17a**	I	7.5/9.3	260	Y = 2978*X + 6.658<br>Y = 2723*X - 1.306
Amine substrate screening				
**5**	II	3.5	286	Y = 4878*X + 12.52
**5c**	II	7.1	286	Y = 6619*X + 22.67
**5d**	II	7.9	286	Y = 6601*X + 38.00
**5e**	II	9.3	286	Y = 6822*X + 14.26
**5f**	II	8.2	286	Y = 7009*X + 24.88
**5g**	II	8.2	286	Y = 7232*X + 51.12
**5h**	II	7.6	286	Y = 7275*X + 20.87
**5i**	II	7.4	286	Y = 6623*X + 26.75*
**5j**	II	8.7	286	Y = 6623*X + 26.75
**5k**	II	7.4	286	Y = 6479*X + 25.74
Pharmaceutical amide synthesis				
**18/18l**	I	5.3/3.8	300	Y = 3405*X + 35.55/<br>Y = 3608*X + 19.31
**19/19m**	I	5.5/7.5	320	Y = 2906*X + 39.18/<br>Y = 3323*X + 37.17
**11n**	I	3.8	260	Y = 2200*X + 11.96
**20/20o****	I	2.9/1.8	230	-

*Calibration curve of **5i** was obtained from **5j** due to structure similarity.

Conversion of **20 to **20o** was calculated based on peak areas by HPLC analysis.

7.2 Chiral HPLC

Samples were analysed on the Agilent 1200 LC System, with diode array UV detector, fitted with either the CHIRALPAK® IB column, 4.6 mm x 250 mm, 5 μm, the CHIRALPAK® IC column, 4.6 mm x 250 mm, 5 μm, or the CHIRALPAK® AD-H column, 4.6 x 150 mm, 5 μm. 80% hexane and 20% isopropanol were used as eluents, 25 °C and 1.0 mL min^{-1} were applied for both columns. Retention times of each enantiomer and analysis details are listed in **Table S5**.

Table S5. Retention times of chiral amide products.

Compounds	Column	Retention times/min	Wavelength/nm
(*S*)/(*R*)-12a	AD-H	4.1/4.7	280
(*S*)/(*R*)-13a	IB	6.2/5.9	210
(*S*)/(*R*)-14a	IB	5.2/4.9	210
(*S*)/(*R*)-5c	IB	11.2/9.6	280
(*S*)/(*R*)-5d	IB	13.1/15.7	280
(*S*)/(*R*)-5e	IB	6.0/6.3	280
(*S*)/(*R*)-5f	IB	22.2/15.7	280
(*S*)/(*R*)-5g	IC	48.0/27.2	280
(*S*)/(*R*)-5h	IC	22.4/18.5	280

7.3 LC-MS

High Performance Liquid Chromatography-Electrospray Ionization Mass Spectrometry (LC-MS) of pharmaceutical amides was performed using a Dionex UltiMate® 3000 Ci Rapid Separation LC system equipped with an UltiMate® 3000 photodiode array detector probing at 210–400 nm, coupled to a HCT ultra ETD II (Bruker Daltonics) ion trap spectrometer, using Chromeleon® 6.80 SR12 software

(ThermoScientific), esquireControl version 6.2, Build 62.24 software (Bruker Daltonics), and Bruker compass HyStar 3.2-SR2, HyStar version 3.2, Build 44 software (Bruker Daltonics) at the University York Centre of Excellence in Mass Spectrometry (CoEMS). Electrospray ionization (ESI) mass spectra of amides were obtained on a Bruker Solarix XR 9.4 T FTICR mass spectrometer. Mass spectrometry data analysis was performed using ESI Compass 1.3 DataAnalysis, version 4.4 software (Bruker Daltonics). All mass spectrometry was conducted in positive ion mode unless stated otherwise. Scan range was 100-600 *m*/*z*.

Samples were prepared and analysed using an Agilent Poroshell 120 EC-C18 column, 2,7 µm, 4,6 x 150 mm (**Table S6**). The eluents employed were: A) 0.1% v/v solution of formic acid in water; B) 0.1% v/v solution of formic acid in MeCN. A multistep gradient method was programmed as follow: 10% to 80% of B in 20 min, hold for 5 min; 80% to 10% of B in 0.5 min, hold for 5 min for re-equilibration. The column temperature was 35 °C and flow rate was 0.4 mL min^{-1}.

Table S6. Pharmaceutical amide targets and estimated conversion by LC-MS.

Name	Function	Structure	Conversion	$[M+H]^+$ calculated	$[M+H]^+$ found
Cinepazide	cardiovascular and cerebrovascular treatment	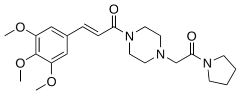	52%	418.23	418.17
Ilepcimide	Anticonvulsant	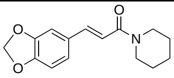	16%	260.13	259.88
Lazabemide	Antiparkinson	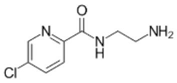	14%	200.06	199.72
Trimethobenzamide	Nausea and vomiting treatment	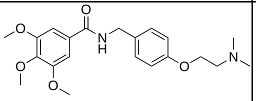	4%	389.21	389.07
Moclobemide	Depressive disorders treatment	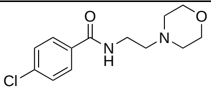	2%	269.11	268.87
Procainamide	arrhythmias treatment	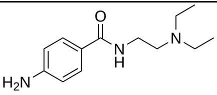	<1%	236.18	235.92
Precursor of losmapimod	Cardiovascular disease treatment	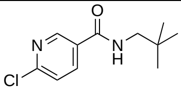	<1%	227.10	226.81

Section 8. Chiral HPLC and LC-MS Chromatograms

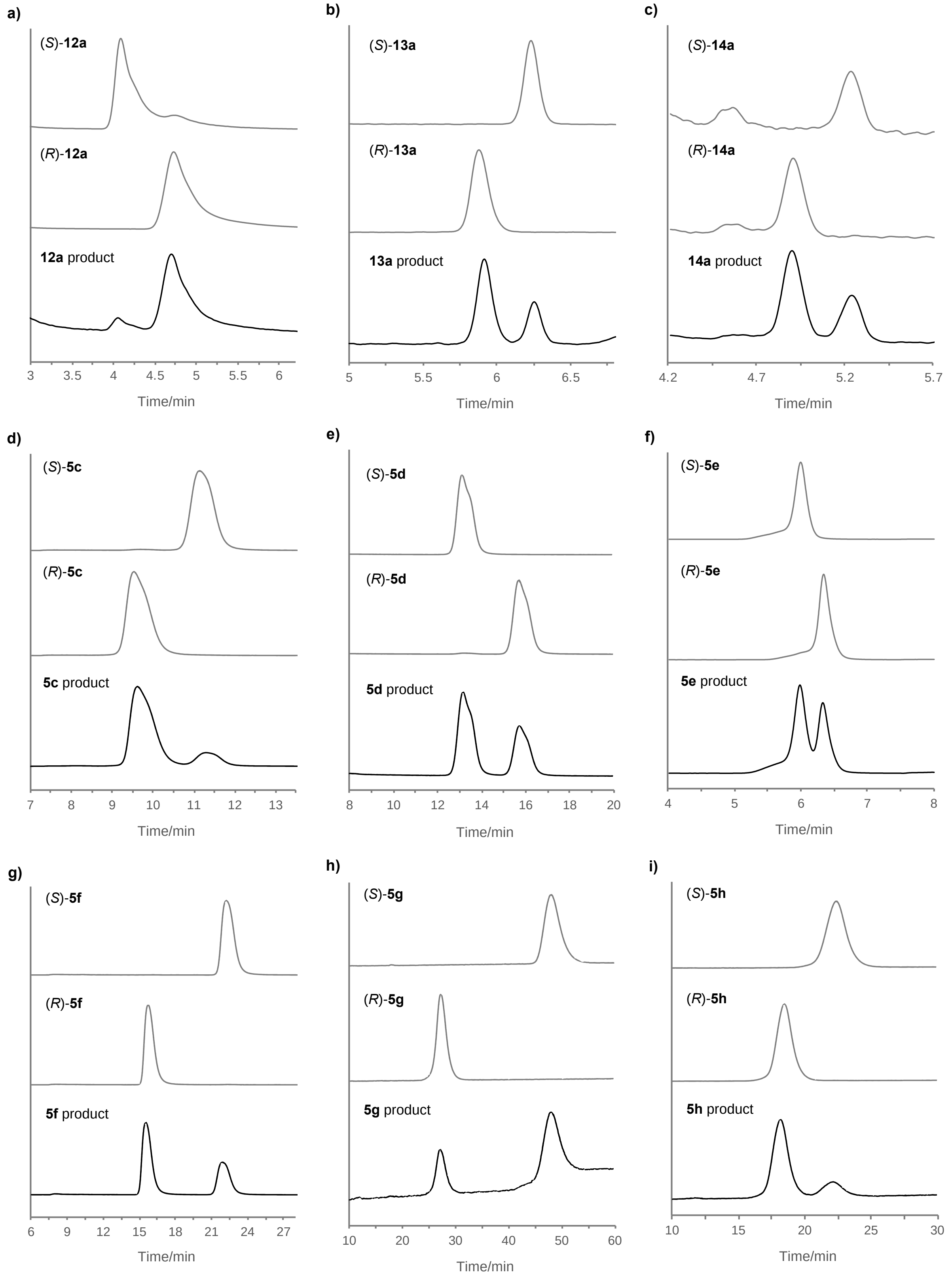

Figure S9. Chiral HPLC chromatograms of **12a-14a** and **5c-5h**. Both enantiomer standards and enzymatic products were shown.

Section 9. Compound characterization and NMR spectra

Data for compounds **5a-9a**, **5b-9b**, **5c-9c**, **5d-9d**, **10a**, **16a** and **5i** were in accord with those previously described.[5,6]

3,4,5-Trimethoxy-*N*-(2-phenylethyl)benzamide (11a)

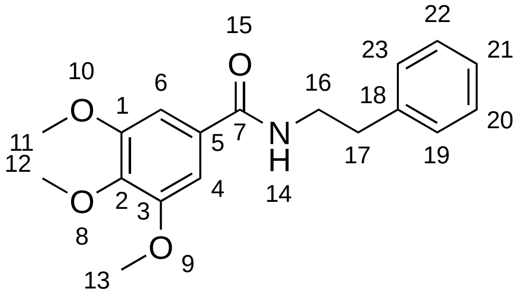

11a

White solid: **mp** 127-129 °C; **R$_f$** = 0.15 (EtOAc:Hex, 1:2, v/v); **IR** (thin film, cm $^{-1}$): 3296 (NH, w), 3104, 3083, 3067, 3026, 2994, 2982, 2937, 2839, 1631 (-NH-CO, m), 1580, 1538, 1503, 1454, 1436, 1411, 1364, 1340, 1281, 1234, 1195, 1179, 1128, 1056, 1022, 991, 932, 899, 842, 810, 779, 726, 693, 667; **^{1}H NMR (CDCl$_3$, 400 MHz)** δ 7.24 (2H, ddd, *J* = 7.6, 4.9, 3.4 Hz, H-20, H-22), 7.21 – 7.11 (3H, m, H-19, H-21, H-23), 6.96 (2H, s, H-4, H-6), 6.82 (1H, s, H-14), 3.81 (3H, s, H-12), 3.76 (6H, s, H-11, H-13), 3.61 (2H, q, *J* = 6.5 Hz, H-16), 2.86 (2H, t, *J* = 7.1 Hz, H-17); **^{13}C NMR (CDCl$_3$, 101 MHz)** δ 167.23 (C-7), 152.98 (C-1, C-3), 140.53 (C-2), 138.87 (C-18), 130.01 (C-5), 128.70 (C-19, C-23), 128.51 (C-20, C-22), 126.44 (C-21), 104.21 (C-4, C-6), 60.75 (C-12), 56.00 (C-11, C-13), 41.25 (C-16), 35.56 (C-17); **HRMS (ESI+, *m/z*):** calculated for $(C_{18}H_{22}NO_4)^+$ [(M+H)$^+$]: 316.1548; found: 316.1543.

2-(4-hydroxyphenoxy)-*N*-(2-phenylethyl)propanamide (12a)

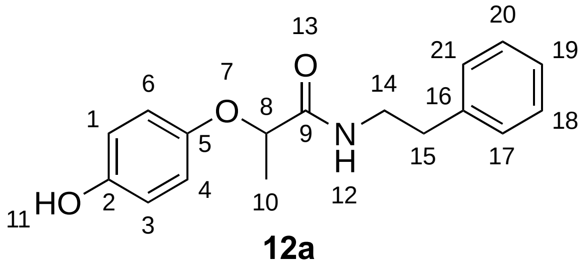

12a

Light yellow liquid: **R$_f$** = 0.08 (EtOAc:Hex, 1:3, v/v); **IR** (thin film, cm $^{-1}$): 3400, 3302, 3086, 3061, 3028, 2984, 2935, 2871, 1650 (-NH-CO, m), 1601, 1536, 1505, 1452, 1366, 1332, 1301, 1264, 1209, 1083, 1030, 1007, 938, 877, 826, 734, 699; **^{1}H NMR (CDCl$_3$, 600 MHz)** δ 8.10 (1H, s, H-11), 7.29 (2H, t, *J* = 7.3 Hz, H-18, H-20), 7.24 (1H, t, *J* = 7.3 Hz, H-19), 7.12 (2H, d, *J* = 6.9 Hz, H-17, H-21), 6.87 (2H, d, *J* = 8.9 Hz, H-1,

H-3), 6.83 (1H, t, H-12), 6.73 (2H, d, *J* = 9.0 Hz, H-4, H-6), 4.58 (1H, q, *J* = 6.7 Hz, H-8), 3.71 – 3.49 (2H, m, H-14), 2.82 (2H, ddt, *J* = 44.8, 13.9, 6.9 Hz, H-15), 1.54 (3H, d, *J* = 6.8 Hz, H-10); **^{13}C NMR (CDCl$_3$, 151 MHz)** δ 173.41 (C-9), 151.86 (C-2), 150.22 (C-5), 138.29 (C-16), 128.72 (C-18, C-20 or C-17, C-21), 128.62 (C-18, C-20 or C-17, C-21), 126.55 (C-19), 116.81 (C-4, C-6), 116.33 (C-1, C-3), 75.77 (C-8), 40.28 (C-14), 35.50 (C-15), 18.86 (C-10); **HRMS (ESI+, *m/z*):** calculated for $(C_{17}H_{20}NO_3)^+$[(M+H)$^+$]: 286.1443; found: 286.1438.

2-Phenyl-*N*-(2-phenylethyl)propanamide (13a)

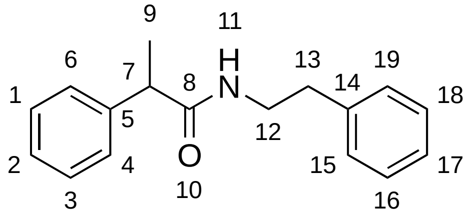

13a

White solid: **mp** 88-89 °C; **R$_f$** = 0.18 (EtOAc:Hex, 1:5, v/v); **IR** (thin film, cm $^{-1}$): 3245 (NH, w), 3061, 3030, 2967, 2933, 2900, 2873, 1656, 1634 (-NH-CO, m), 1599, 1554, 1495, 1452, 1354, 1336, 1305, 1260, 1232, 1193, 1150, 1071, 1030, 989, 967, 910, 883, 840, 785, 753, 720, 697; **^{1}H NMR (CDCl$_3$, 500 MHz)** δ 7.36 – 7.12 (8H, m, H-1, H-2, H-3, H-4, H-6, H-16, H-17, H-18), 7.00 (2H, d, *J* = 6.6 Hz, H-15, H-19), 5.83 (1H, s, H-11), 3.56 – 3.43 (2H, m, H-7, H-12'), 3.41 – 3.32 (1H, m, H-12''), 2.70 (1H, t, *J* = 7.7 Hz, H-13), 1.50 (3H, d, *J* = 7.2 Hz, H-9); **^{13}C NMR (CDCl$_3$, 126 MHz)** δ 174.08 (C-8), 141.31 (C-5), 138.77 (C-14), 128.72 (C-1, C-3), 128.62 (C-15, C-19), 128.41 (C-16, C-18), 127.51 (C-4, C-6), 127.05 (C-2), 126.23 (C-17), 46.86 (C-7), 40.68 (C-12), 35.44 (C-13), 18.32 (C-9); **HRMS (ESI+, *m/z*):** calculated for $(C_{17}H_{20}NO)^+$[(M+H)$^+$]: 254.1545; found: 254.1539.

2-[4-(2-methylpropyl)phenyl]-*N*-(2-phenylethyl)propanamide (14a)

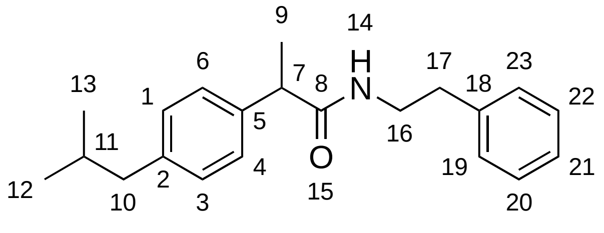

14a

White solid: **mp** 103-105 °C; **R$_f$** = 0.20 (EtOAc:Hex, 1:5, v/v); **IR** (thin film, cm $^{-1}$): 3253 (NH, w), 3068, 3026, 2968, 2954, 2933, 2867, 2848, 1632 (-NH-CO, m), 1548, 1510, 1497, 1468, 1454, 1422, 1381, 1364, 1351, 1288, 1236, 1193, 1169, 1104, 1074, 1027, 1004, 915, 856, 783, 760, 748, 699; **^{1}H NMR (CDCl$_3$, 600 MHz)** δ 7.25 – 7.16 (3H, m,

H-20, H-21, H-22), 7.10 (4H, q, H-1, H-3, H-4, H-6), 7.00 (1H, d, *J* = 6.6 Hz, H-19, H-23), 3.54 – 3.45 (2H, m, H-7, H-16'), 3.38 (1H, dq, *J* = 13.6, 6.9 Hz, H-16''), 2.70 (2H, td, *J* = 6.8, 3.1 Hz, H-17), 2.47 (2H, d, *J* = 7.2 Hz, H-10), 1.86 (1H, dp, *J* = 13.6, 6.8 Hz, H-11), 1.49 (3H, d, *J* = 7.2 Hz, H-9), 0.92 (6H, d, *J* = 6.6 Hz, H-12, H-13); **^{13}C NMR ($CDCl_3$, 151 MHz)** δ 174.52 (C-8), 140.78 (C-2), 138.94 (C-18), 138.49 (C-5), 129.70 (C-1, C-3), 128.83 (C-19, C-23), 128.61 (C-20, C-22), 127.48 (C-4, C-6), 126.45 (C-21), 46.86 (C-7), 45.14 (C-10), 40.79 (C-16), 35.65 (C-17), 30.29 (C-11), 22.51 (C-12, C-13), 18.42 (C-9); **HRMS (ESI+, *m/z*):** calculated for $(C_{21}H_{28}NO)^+$[$(M+H)^+$]: 310.2171; found: 310.2165.

(2*E*)-3-Phenyl-*N*-(2-phenylethyl)prop-2-enamide (15a)

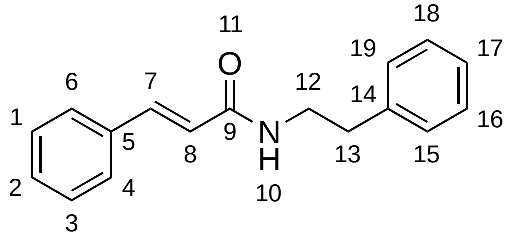

15a

White solid: **mp** 126-128 °C; **R_f** = 0.10 (EtOAc:Hex, 1:5, v/v); **IR** (thin film, cm $^{-1}$): 3300 (NH, w), 3059, 3028, 2937, 2875, 2859, 1650 (-NH-CO, m), 1613, 1576, 1540, 1493, 1450, 1332, 1283, 1215, 1193, 1031, 967, 908, 865, 750, 720, 699; **^{1}H NMR ($CDCl_3$, 500 MHz)** δ 7.66 (1H, d, *J* = 15.7 Hz, H-7), 7.44 (2H, dd, *J* = 7.7, 1.9 Hz, H-4, H-6), 7.31 – 7.24 (5H, m, H-1, H-2, H-3, H-16, H-18), 7.23 – 7.17 (3H, m, H-15, H-17, H-19), 6.83 (1H, t, *J* = 5.9 Hz, H-10), 6.54 (1H, d, *J* = 15.7 Hz, H-8), 3.65 (2H, dd, *J* = 13.9, 6.5 Hz, H-12), 2.90 (2H, t, *J* = 7.3 Hz, H-13); **^{13}C NMR ($CDCl_3$, 126 MHz)** δ 166.23 (C-9), 140.59 (C-7), 138.85 (C-14), 134.78 (C-5), 129.52 (C-2), 128.71 (C-1, C-3), 128.66 (C-15, C-19), 128.53 (C-16, C-18), 127.71 (C-4, C-6), 126.39 (C-17), 121.07 (C-8), 41.00 (C-12), 35.67 (C-13); **HRMS (ESI+, *m/z*):** calculated for $(C_{17}H_{18}NO)^+$[$(M+H)^+$]: 252.1388; found: 252.1383.

2-(3-Benzoylphenyl)-*N*-(2-phenylethyl)propanamide (17a)

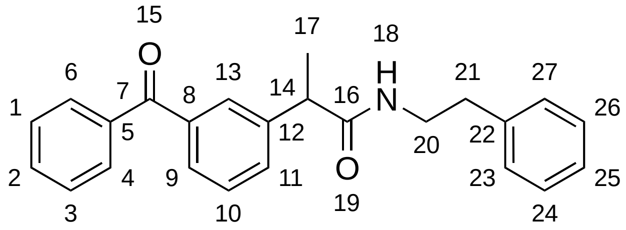

17a

Transparent liquid; **R_f** = 0.11 (EtOAc:Hex, 1:3, v/v); **IR** (thin film, cm $^{-1}$): 3306 (NH, w), 3061, 3028, 2971, 2931, 2871, 1646 (-NH-CO, s), 1597, 1578, 1534, 1497, 1448, 1366,

1317, 1283, 1228, 1197, 1179, 1138, 1073, 1030, 999, 954, 910, 820, 722, 697, 642; **^{1}H NMR ($CDCl_3$, 500 MHz)** δ 7.75 – 7.67 (3H, m, H-4, H-6, H-13), 7.58 (1H, dt, *J* = 7.7, 1.4 Hz, H-9), 7.50 (2H, t, *J* = 7.4 Hz, H-2, H-11), 7.43 – 7.30 (3H, m, H-1, H-3, H-10), 7.13 (2H, t, *J* = 7.2 Hz, H-24, H-26), 7.10 – 7.04 (1H, m, H-25), 6.97 (2H, d, *J* = 6.7 Hz, H-23, H-27), 6.48 (1H, q, *J* = 6.0 Hz, H-18), 3.58 (1H, q, *J* = 7.1 Hz, H-14), 3.47 – 3.37 (1H, m, H-20'), 3.37 – 3.28 (1H, m, H-20''), 2.66 (2H, t, *J* = 7.1 Hz, H-21), 1.45 (3H, d, *J* = 7.1 Hz, H-17); **^{13}C NMR ($CDCl_3$, 126 MHz)** δ 196.30 (C-7), 173.46 (C-16), 141.86 (C-12), 138.59 (C-22), 137.50 (C-8), 137.16 (C-5), 132.31 (C-2), 131.37 (C-11), 129.74 (C-4, C-6), 128.85 (C-13), 128.63 (C-9), 128.42 (C-23, C-27), 128.36 (C-10), 128.19 (C-24, C-26), 128.08 (C-1, C-3), 126.06 (C-25), 46.30 (C-14), 40.61 (C-20), 35.25 (C-21), 18.33 (C-17); **HRMS (ESI+, *m/z*):** calculated for $(C_{24}H_{24}NO_2)^+$[(M+H)+]: 358.1807; found: 358.1802.

1-Acetyl-*N*-(2-aminooctane)-*9H*-β-carboline-3-carboxamide (5e)

(or **1-Acetyl-*N*-(octan-2-yl)-*9H*-pyrido[3,4-b]indole-3-carboxamide** ??**)**

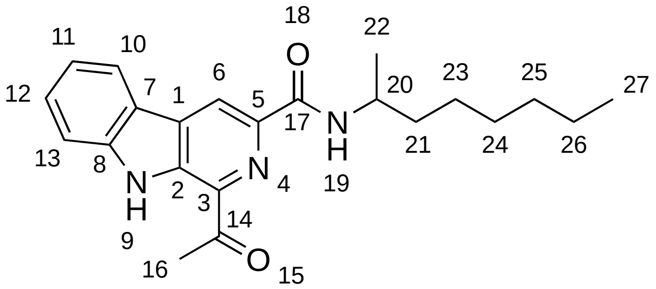

5e

Yellow solid: **mp** 157-159 °C; **R$_f$** = 0.26 (EtOAc:Hex, 1:5, v/v); **IR** (thin film, cm $^{-1}$): 3381, 3336 (NH, w), 2953, 2926, 2855, 1664 (-NH-CO, m), 1627, 1595, 1520, 1495, 1446, 1362, 1305, 1287, 1246, 1213, 1183, 1156, 1056, 1018, 961, 903, 887, 850, 791, 746, 740, 679; **^{1}H NMR ($CDCl_3$, 500 MHz)** δ 10.45 (1H, s, H-9), 9.06 (1H, s, H-6), 8.14 (1H, d, *J* = 7.8 Hz, H-10), 7.84 (1H, d, *J* = 8.7 Hz, H-19), 7.62 – 7.51 (2H, m, H-12, H-13), 7.33 (1H, ddd, *J* = 8.0, 6.6, 1.4 Hz, H-11), 4.34 – 4.20 (1H, m, H-20), 2.89 (3H, s, H-16), 1.71 – 1.57 (2H, m, H-21), 1.48 – 1.39 (2H, m, H-23), 1.39 – 1.34 (1H, m, H-24), 1.33 (3H, d, *J* = 6.6 Hz, H-22), 1.28 (4H, dt, *J* = 7.2, 3.7 Hz, H-25, H-26), 0.88 – 0.84 (2H, m, H-27); **^{13}C NMR ($CDCl_3$, 126 MHz)** δ 202.15 (C-14), 163.98 (C-17), 141.64 (C-8), 139.56 (C-5), 136.25 (C-2), 133.49 (C-3), 132.64 (C-1), 129.67 (C-12), 122.27 (C-10), 121.47 (C-11), 121.08 (C-7), 118.42 (C-6), 112.25 (C-13), 45.48 (C-20), 37.25 (C-21), 31.87 (C-25), 29.33 (C-24), 26.13 (C-23), 25.71 (C-16), 22.68 (C-26), 21.21 (C-22), 14.15 (C-27); **HRMS (ESI+, *m/z*):** calculated for $(C_{22}H_{27}N_3O_2)^+$ [(M+H)$^+$]: 366.2182; found: 366.2176.

1-Acetyl-*N*-(1-(2-naphthyl)ethylamine)-*9H*-β-carboline-3-carboxamide (5f)

(or **1-Acetyl-*N*-[1-(naphthalen-2-yl)ethyl]-*9H*-pyrido[3,4-b]indole-3-carboxamide** ??)

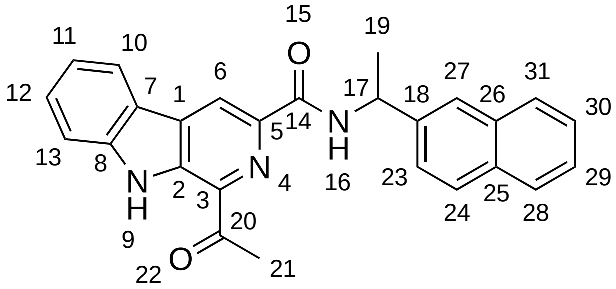

5f

Yellow solid: **mp** 200-203 °C; $\mathbf{R_f}$ = 0.16 (EtOAc:Hex, 1:5, v/v); **IR** (thin film, cm $^{-1}$): 3369 (NH, w), 3053, 2971, 2928, 1662 (-NH-CO, w), 1625, 1595, 1513, 1491, 1460, 1446, 1372, 1358, 1330, 1287, 1242, 1207, 1179, 1150, 1111, 1058, 1012, 961, 905, 887, 850, 814, 789, 738; **^{1}H NMR ($CDCl_3$, 600 MHz)** δ 10.49 (1H, s, H-9), 9.07 (1H, s, H-6), 8.40 (1H, d, *J* = 8.3 Hz, H-16), 8.11 (1H, d, *J* = 7.9 Hz, H-10), 7.91 (1H, s, H-27), 7.87 – 7.78 (3H, m, H-24, H-28, H-31), 7.61 (1H, dd, *J* = 8.6, 1.8 Hz, H-23), 7.58 – 7.50 (2H, m, H-12, H-13), 7.46 (2H, pd, *J* = 6.9, 1.5 Hz, H-29, H-30), 7.33 (1H, dd, *J* = 7.9, 6.8 Hz, H-11), 5.64 (1H, p, *J* = 7.1 Hz, H-17), 2.86 (3H, s, H-21), 1.82 (3H, d, *J* = 6.9 Hz, H-19); **^{13}C NMR ($CDCl_3$, 151 MHz)** δ 202.01 (C-20), 163.98 (C-14), 141.61 (C-8), 140.97 (C-18), 139.15 (C-5), 136.23 (C-2), 133.47 (C-3, C-26), 132.80 (C-25), 132.57 (C-1), 129.68 (C-12), 128.64 (C-24), 127.96 (C-31), 127.68 (C-28), 126.28 (C-29), 125.91 (C-30), 124.71 (C-23), 124.58 (C-27), 122.21 (C-10), 121.48 (C-11), 120.98 (C-7), 118.48 (C-6), 112.28 (C-13), 49.01 (C-17), 25.71 (C-21), 22.30 (C-19); **HRMS (ESI+, *m/z*):** calculated for $(C_{26}H_{22}N_3O_2)^+[(M+H)^+]$: 408.1712; found: 408.1707.

1-Acetyl-*N*-(1-(1-Naphthyl)ethylamine)-*9H*-β-carboline-3-carboxamide (5g)

(or 1-Acetyl-*N*-[1-(naphthalen-1-yl)ethyl]-*9H*-pyrido[3,4-b]indole-3-carboxamide ??)

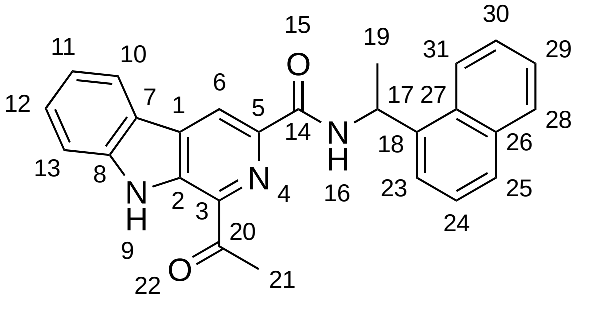

5g

Yellow solid: **mp** 233-234 °C; $\mathbf{R_f}$ = 0.16 (EtOAc:Hex, 1:5, v/v); **IR** (thin film, cm $^{-1}$): 3393, 3345 (NH, w), 3041, 2971, 2928, 2871, 1660 (-NH-CO, m), 1625, 1595, 1513, 1489, 1444, 1395, 1370, 1356, 1328, 1285, 1238, 1205, 1175, 1144, 1107, 1091, 1058, 1009, 999, 963, 908, 885, 854, 795, 775, 759, 740, 675; **^{1}H NMR ($CDCl_3$, 500 MHz)** δ 10.40

(1H, s, H-9), 9.13 (1H, s, H-6), 8.37 (1H, d, *J* = 8.6 Hz, H-16), 8.31 (1H, d, *J* = 8.6 Hz, H-31), 8.18 (1H, dd, *J* = 7.9, 1.0 Hz, H-10), 7.90 – 7.86 (1H, m, H-28), 7.82 (1H, d, *J* = 8.2 Hz, H-25), 7.68 (1H, d, *J* = 7.1 Hz, H-23), 7.62 – 7.53 (3H, m, H-12, H-13, H-30), 7.53 – 7.47 (2H, m, H-24, H-29), 7.35 (1H, ddd, *J* = 8.0, 6.9, 1.2 Hz, H-11), 6.24 (1H, dq, *J* = 8.7, 6.9 Hz, H-17), 2.79 (3H, s, H-21), 1.87 (3H, d, *J* = 6.8 Hz, H-19); **^{13}C NMR ($CDCl_3$, 126 MHz)** δ 202.23 (C-20), 163.77 (C-14), 141.65 (C-8), 139.25 (C-5), 139.00 (C-18), 136.41 (C-2), 134.18 (C-26), 133.60 (C-3), 132.70 (C-1), 131.22 (C-27), 129.82 (C-12), 128.98 (C-28), 128.43 (C-25), 126.63 (C-30), 125.94 (C-24), 125.49 (C-29), 123.63 (C-31), 122.70 (C-23), 122.36 (C-10), 121.62 (C-11), 121.12 (C-7), 118.67 (C-6), 112.30 (C-13), 45.31 (C-17), 25.77 (C-21), 21.83 (C-19); **HRMS (ESI+, *m/z*):** calculated for $(C_{26}H_{22}N_3O_2)^+$ [$(M+H)^+$]: 408.1712; found: 408.1707.

1-Acetyl-*N*-(1-phenylpropylamine)-*9H*-β-carboline-3-carboxamide (5h)

(or 1-Acetyl-*N*-(1-phenylpropyl)-*9H*-pyrido[3,4-b]indole-3-carboxamide ??)

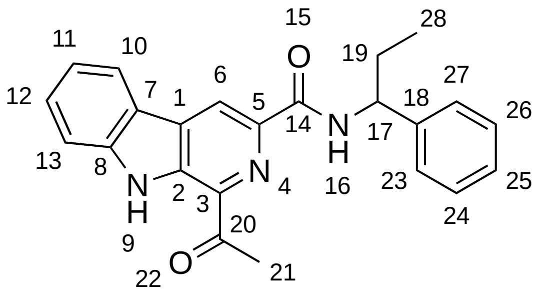

5h

Yellow solid: **mp** 191-193 °C; **R_f** = 0.19 (EtOAc:Hex, 1:5, v/v); **IR** (thin film, cm^{-1}): 3338 (NH, w), 3059, 3030, 2959, 2928, 2871, 1672, 1648 (-NH-CO, m), 1634, 1591, 1525, 1493, 1452, 1364, 1332, 1285, 1260, 1236, 1211, 1181, 1152, 1062, 985, 963, 914, 885, 850, 828, 787, 742, 697; **^{1}H NMR ($CDCl_3$, 500 MHz)** δ 10.45 (1H, s, H-9), 9.01 (1H, s, H-6), 8.35 (1H, d, *J* = 8.6 Hz, H-16), 8.08 (1H, d, *J* = 7.9 Hz, H-10), 7.57 – 7.48 (2H, m, H-12, H-13), 7.44 (2H, d, *J* = 8.1 Hz, H-23, H-27), 7.37 (2H, t, *J* = 7.7 Hz, H-24, H-26), 7.32 – 7.23 (2H, m, H-11, H-25), 5.21 (1H, q, *J* = 7.5 Hz, H-17), 2.86 (3H, s, H-21), 2.11 – 1.97 (2H, m, H-19), 1.02 (3H, t, *J* = 7.4 Hz, H-28); **^{13}C NMR ($CDCl_3$, 126 MHz)** δ 202.01 (C-20), 164.06 (C-14), 142.52 (C-18), 141.60 (C-8), 139.22 (C-5), 136.22 (C-2), 133.44 (C-3), 132.59 (C-1), 129.68 (C-12), 128.77 (C-24, C-26), 127.37 (C-25), 126.63 (C-23, C-27), 122.20 (C-10), 121.48 (C-11), 120.98 (C-7), 118.45 (C-6), 112.27 (C-13), 54.95 (C-17), 29.80 (C-19), 25.67 (C-21), 10.75 (C-28); **HRMS (ESI+, *m/z*):** calculated for $(C_{23}H_{22}N_3O_2)^+$ [$(M+H)^+$]: 372.1712; found: 372.1707.

1-Acetyl-*N*-(4-ethylaniline)-*9H*-β-carboline-3-carboxamide (5j)

(or 1-Acetyl-*N*-(4-ethylphenyl)-*9H*-pyrido[3,4-b]indole-3-carboxamide)

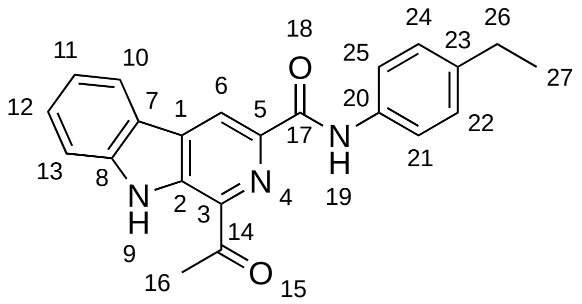

5j

Yellow solid: **mp** 267-269 °C; **R$_f$** = 0.28 (EtOAc:Hex, 1:5, v/v); **IR** (thin film, cm $^{-1}$): 3345 (NH, w), 3020, 2963, 2928, 2871, 2853, 1666 (-NH-CO, m), 1625, 1613, 1591, 1532, 1499, 1460, 1409, 1375, 1354, 1338, 1311, 1298, 1242, 1232, 1209, 1177, 1156, 1116, 1101, 1050, 1018, 963, 908, 885, 865, 832, 814, 787, 767, 750, 736, 677; **^{1}H NMR (CDCl$_3$, 500 MHz)** δ 10.43 (1H, s, H-9), 9.93 (1H, s, H-19), 9.18 (1H, s, H-6), 8.24 (1H, d, *J* = 7.9 Hz, H-10), 7.73 (2H, d, *J* = 8.4 Hz, H-21, H-25), 7.68 – 7.58 (2H, m, H-12, H-13), 7.40 (1H, ddd, *J* = 8.0, 6.8, 1.2 Hz, H-11), 7.26 (3H, d, *J* = 8.3 Hz, H-22, H-24; partially obscured by solvent peak), 2.98 (3H, s, H-16), 2.68 (2H, q, *J* = 7.6 Hz, H-26), 1.27 (3H, t, *J* = 7.6 Hz, H-27); **^{13}C NMR (CDCl$_3$, 126 MHz)** δ 202.10 (C-14), 162.40 (C-17), 141.77 (C-8), 140.53 (C-23), 139.34 (C-5), 136.55 (C-2), 135.66 (C-20), 133.51 (C-3), 133.07 (C-1), 130.04 (C-12), 128.64 (C-22, C-24), 122.50 (C-10), 121.82 (C-11), 121.13 (C-7), 119.97 (C-21, C-25), 118.69 (C-6), 112.38 (C-13), 28.54 (C-26), 25.90 (C-16), 15.85 (C-27); **HRMS (ESI+, *m/z*):** calculated for $(C_{22}H_{19}N_3NaO_2)^+$ [(M+Na)$^+$]: 380.1375; found: 380.1369.

1-Acetyl-*N*-(cyclohexylamine)-*9H*-β-carboline-3-carboxamide (5k)

(1-Acetyl-*N*-cyclohexyl-*9H*-pyrido[3,4-b]indole-3-carboxamide)

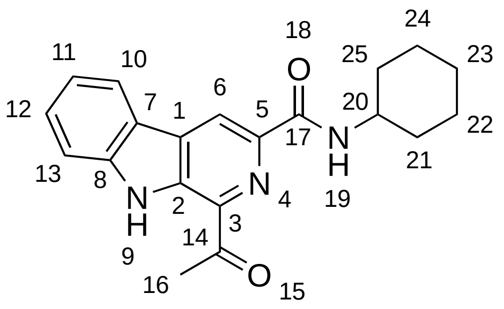

5k

Light yellow solid: **mp** 228-230 °C; **R$_f$** = 0.20 (EtOAc:Hex, 1:5, v/v); **IR** (thin film, cm $^{-1}$): 3371, 3361 (NH, w), 3092, 2933, 2916, 2845, 1678, 1660 (-NH-CO, m), 1627, 1595, 1560, 1523, 1493, 1464, 1452, 1358, 1348, 1330, 1291, 1256, 1242, 1207, 1181, 1154, 1107, 1083, 1060, 1012, 958, 934, 887, 844, 791, 953, 740, 677; **^{1}H NMR (CDCl$_3$, 500 MHz)** δ 10.43 (1H, s, H-9), 9.04 (1H, s, H-6), 8.14 (1H, d, *J* = 7.8 Hz, H-10), 7.94 (1H, d, *J* = 8.5 Hz, H-19), 7.62 – 7.52 (2H, m, H-12, H-13), 7.33 (1H, ddd, *J* = 8.1, 6.8, 1.3

Hz, H-11), 4.14 – 4.03 (1H, m, H-20), 2.87 (3H, s, H-16), 2.13 – 2.05 (2H, m, H-21', H-25'), 1.84 – 1.75 (2H, m, H-22', H-24'), 1.67 (1H, dt, *J* = 13.1, 3.9 Hz, H-23'), 1.55 – 1.35 (4H, m, H-21'', H-25'', H-22'', H-24''), 1.35 – 1.25 (1H, m, H-23''); **^{13}C NMR ($CDCl_3$, 126 MHz)** δ 202.13 (C-14), 163.68 (C-17), 141.61 (C-8), 139.54 (C-5), 136.21 (C-2), 133.44 (C-3), 132.59 (C-1), 129.65 (C-12), 122.28 (C-10), 121.46 (C-11), 121.05 (C-7), 118.37 (C-6), 112.24 (C-13), 48.12 (C-20), 33.29 (C-21, C-25), 25.75 (C-23), 25.69 (C-16), 24.89 (C-22, C-24); **HRMS (ESI+, *m/z*):** calculated for $(C_{20}H_{22}N_3O_2)^+$ $[(M+H)^+]$: 336.1712; found: 336.1707.

(*2E*)-3-(*2H*-1,3-benzodioxol-5-yl)-1-(piperidin-1-yl)prop-2-en-1-one (Ilepcimide, 19m)

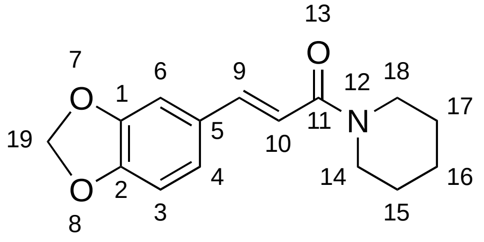

19m

White solid: **mp** 84-86 °C; **R_f** = 0.24 (EtOAc:Hex, 1:2, v/v); **IR** (thin film, cm^{-1}): 3065, 3006, 2939, 2853, 2786, 1642 (-NH-CO, m), 1587, 1491, 1466, 1440, 1356, 1297, 1248, 1213, 1189, 1138, 1124, 1101, 1016, 971, 930, 854, 840, 816, 761; **^{1}H NMR ($CDCl_3$, 600 MHz)** δ 7.47 (1H, d, *J* = 15.4 Hz, H-9), 6.94 (1H, d, *J* = 1.7 Hz, H-6), 6.90 (1H, dd, *J* = 8.0, 1.7 Hz, H-4), 6.71 – 6.63 (2H, m, H-3, H-10), 5.88 (2H, s, H-19), 3.52 (4H, d, *J* = 46.6 Hz, H-14, H-18), 1.62 – 1.55 (2H, m, H-16), 1.55 – 1.47 (4H, m, H-15, H-17); **^{13}C NMR ($CDCl_3$, 151 MHz)** δ 165.31 (C-11), 148.74 (C-2), 148.11 (C-1), 141.81 (C-9), 129.83 (C-5), 123.46 (C-4), 115.63 (C-10), 108.36 (C-3), 106.25 (C-6), 101.32 (C-19), 46.87 (C-14 or C-18), 43.23 (C-14 or C-18), 26.68 (C-15, C-17), 25.55 (C-15, C-17), 24.58 (C-16); **HRMS (ESI+, *m/z*):** calculated for $(C_{15}H_{18}NO_3)^+$$[(M+H)^+]$: 260.1287; found: 260.1281.

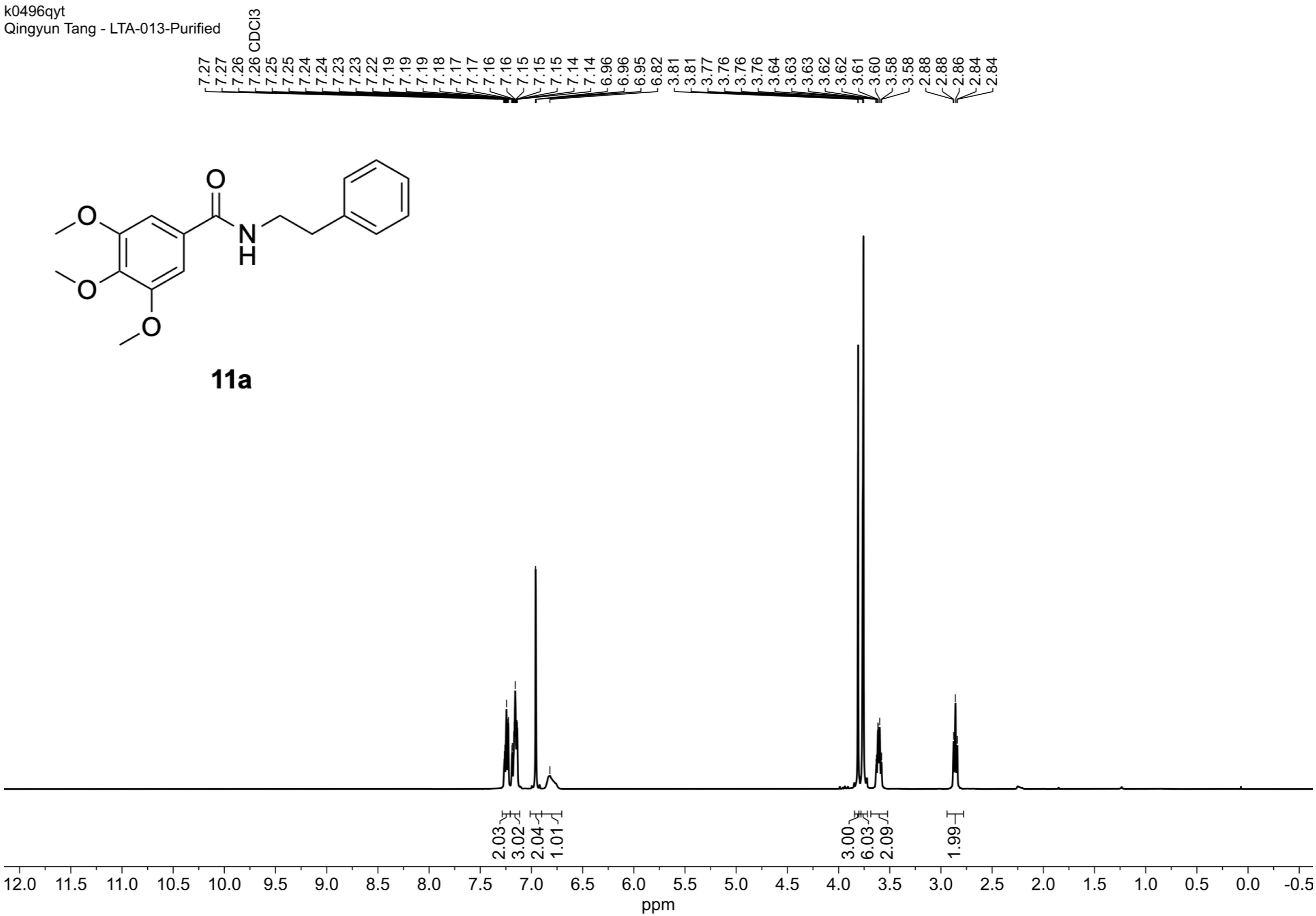

^{1}H NMR spectrum of compound 11a ($CDCl_3$, 400 MHz).

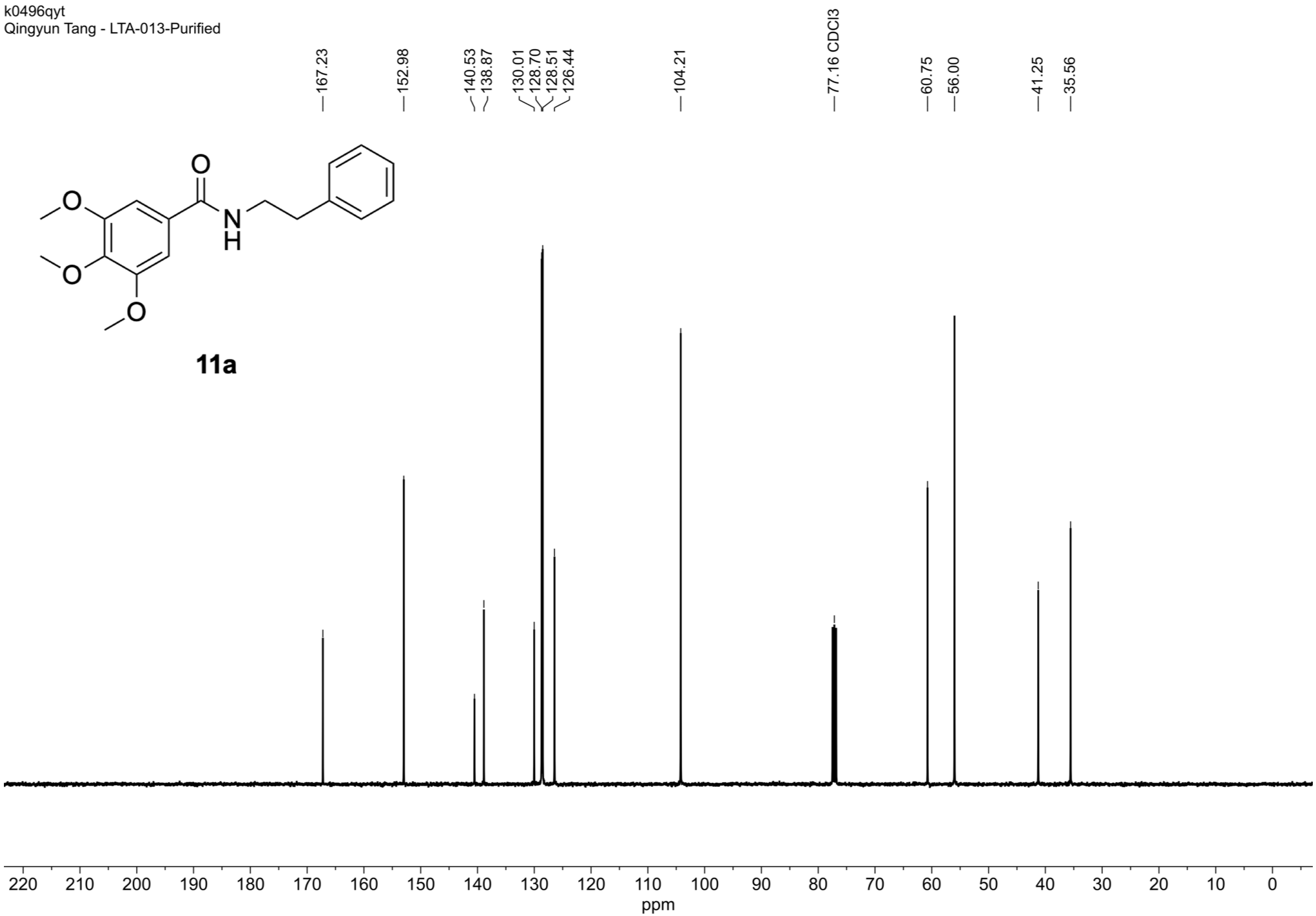

^{13}C NMR spectrum of compound 11a ($CDCl_3$, 101 MHz).

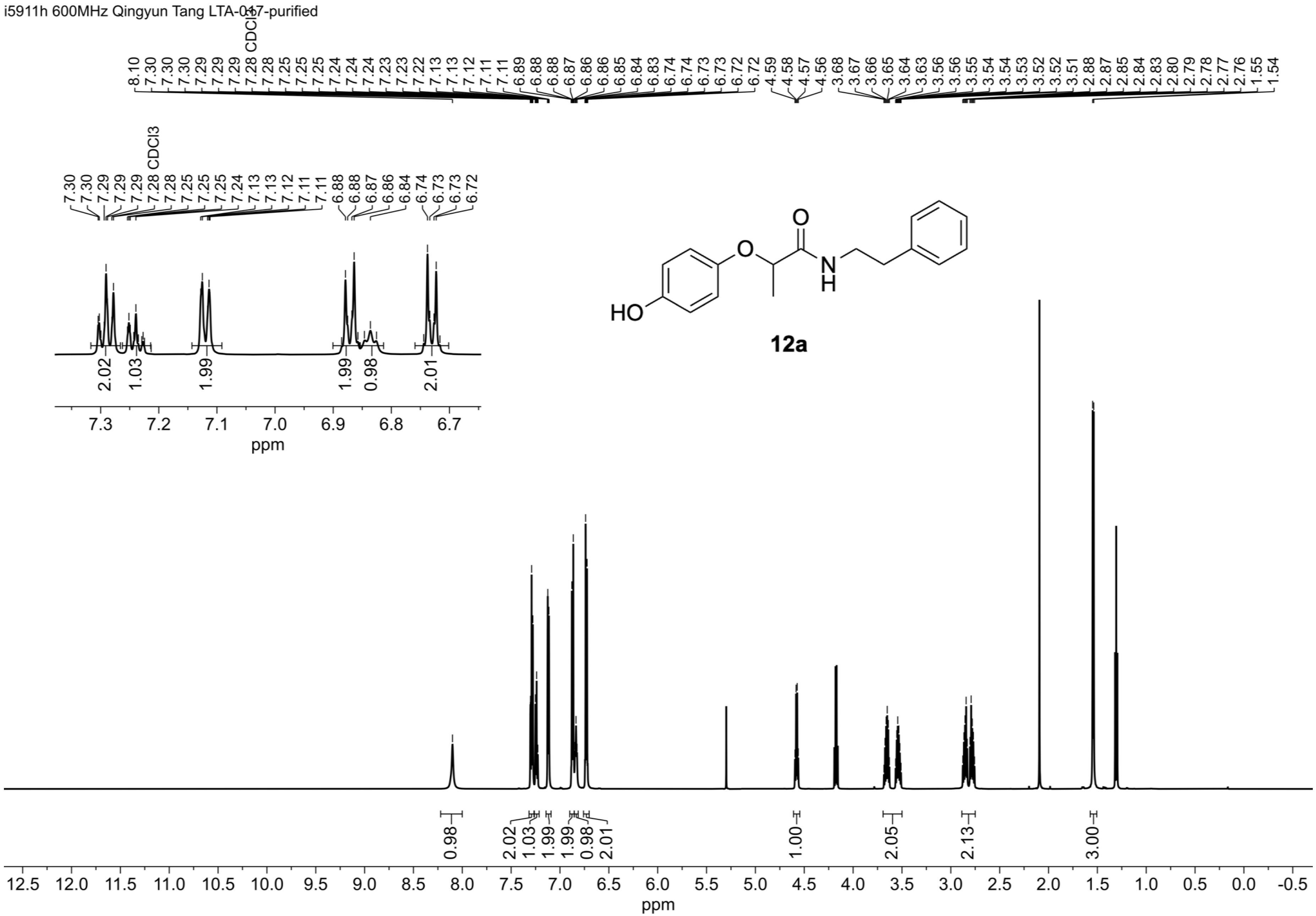

^{1}H NMR spectrum of compound 12a ($CDCl_3$, 600 MHz). Trace EtOAc present at δ 1.31 (t), 2.10 (s) and 4.18 (q).

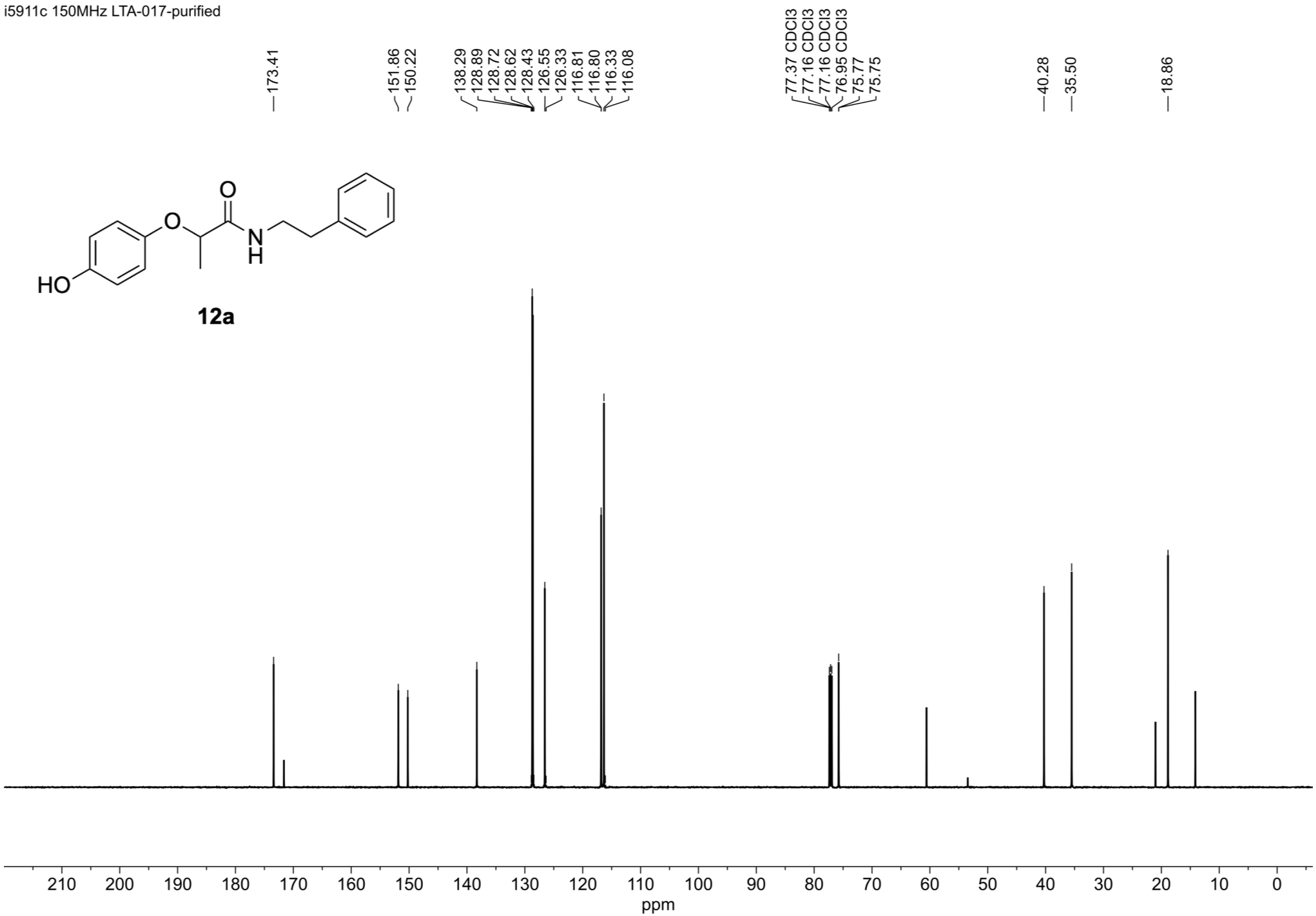

^{13}C NMR spectrum of compound 12a ($CDCl_3$, 151 MHz). Trace EtOAc present at δ 21.02, 60.58 and 171.63.

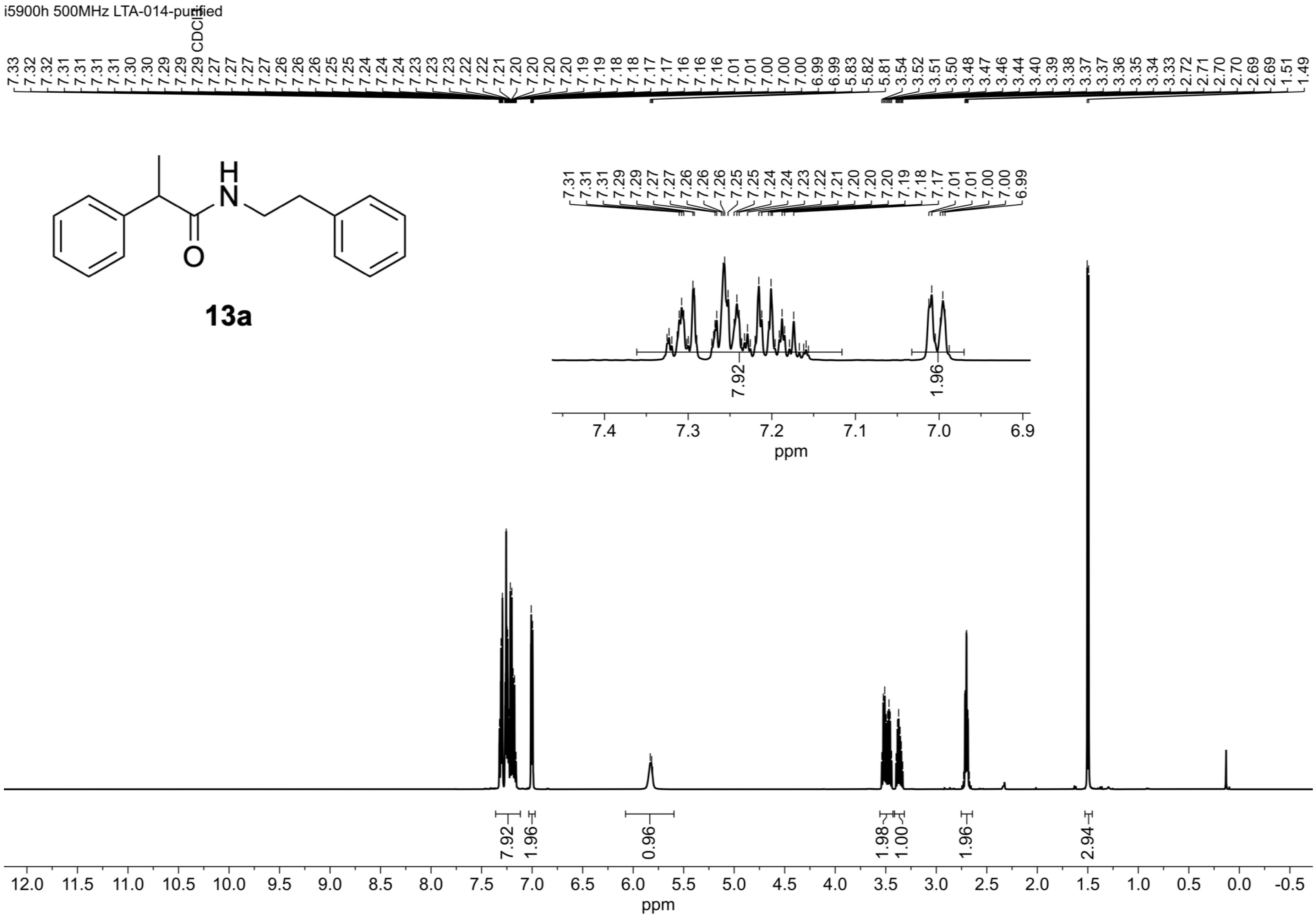

^{1}H NMR spectrum of compound 13a ($CDCl_3$, 500 MHz).

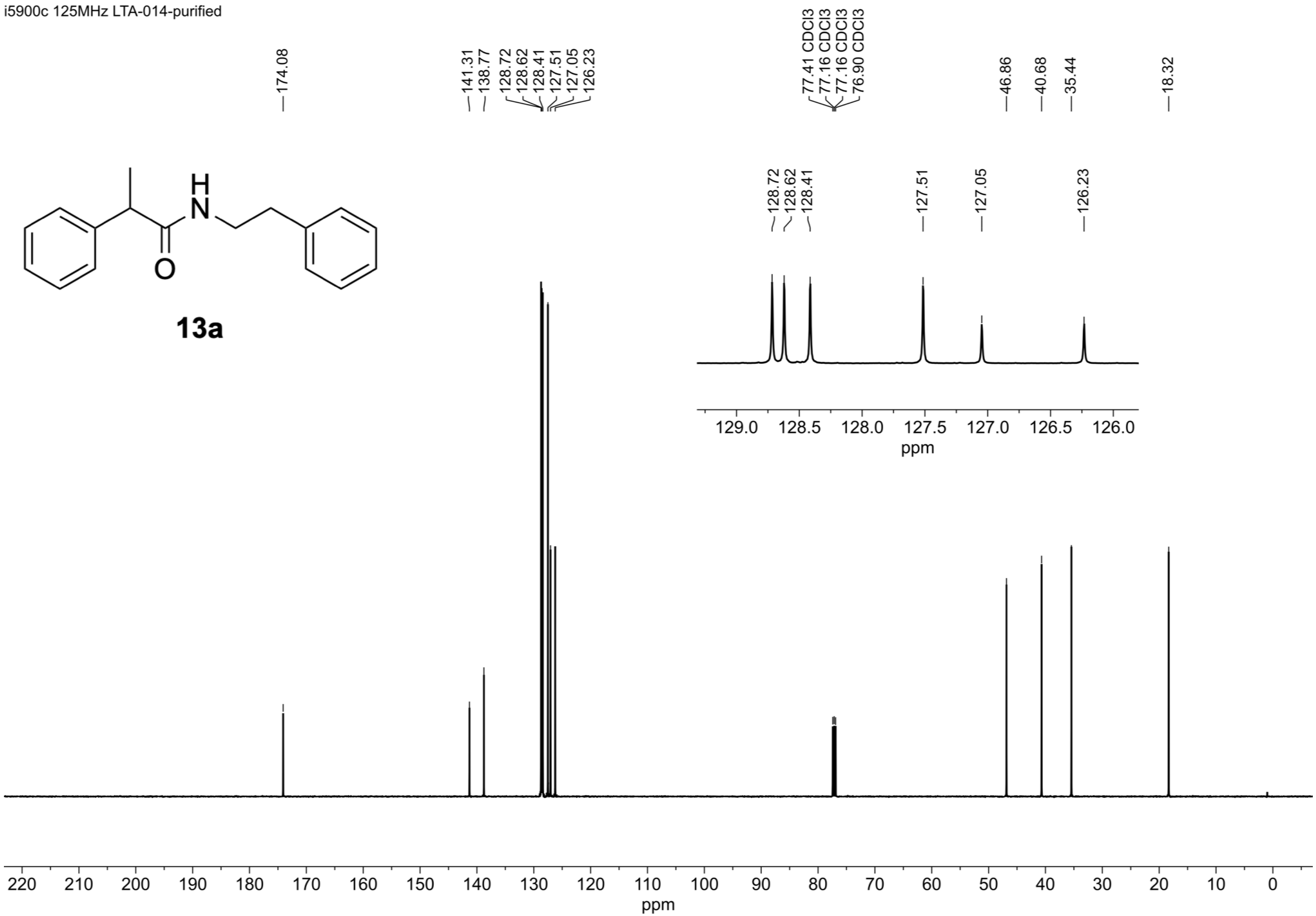

^{13}C NMR spectrum of compound 13a ($CDCl_3$, 126 MHz).

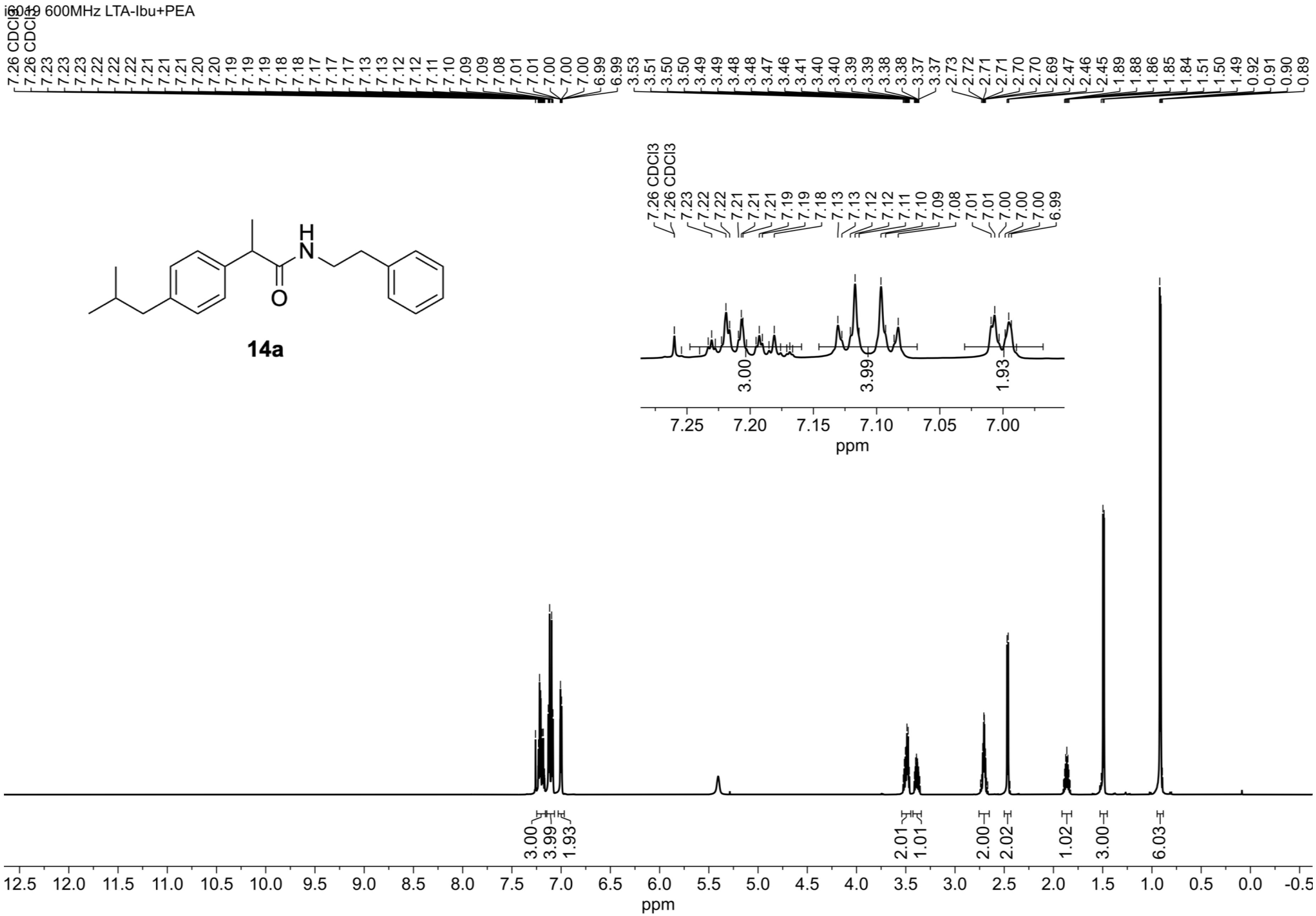

14a

^{1}H NMR spectrum of compound 14a ($CDCl_3$, 600 MHz).

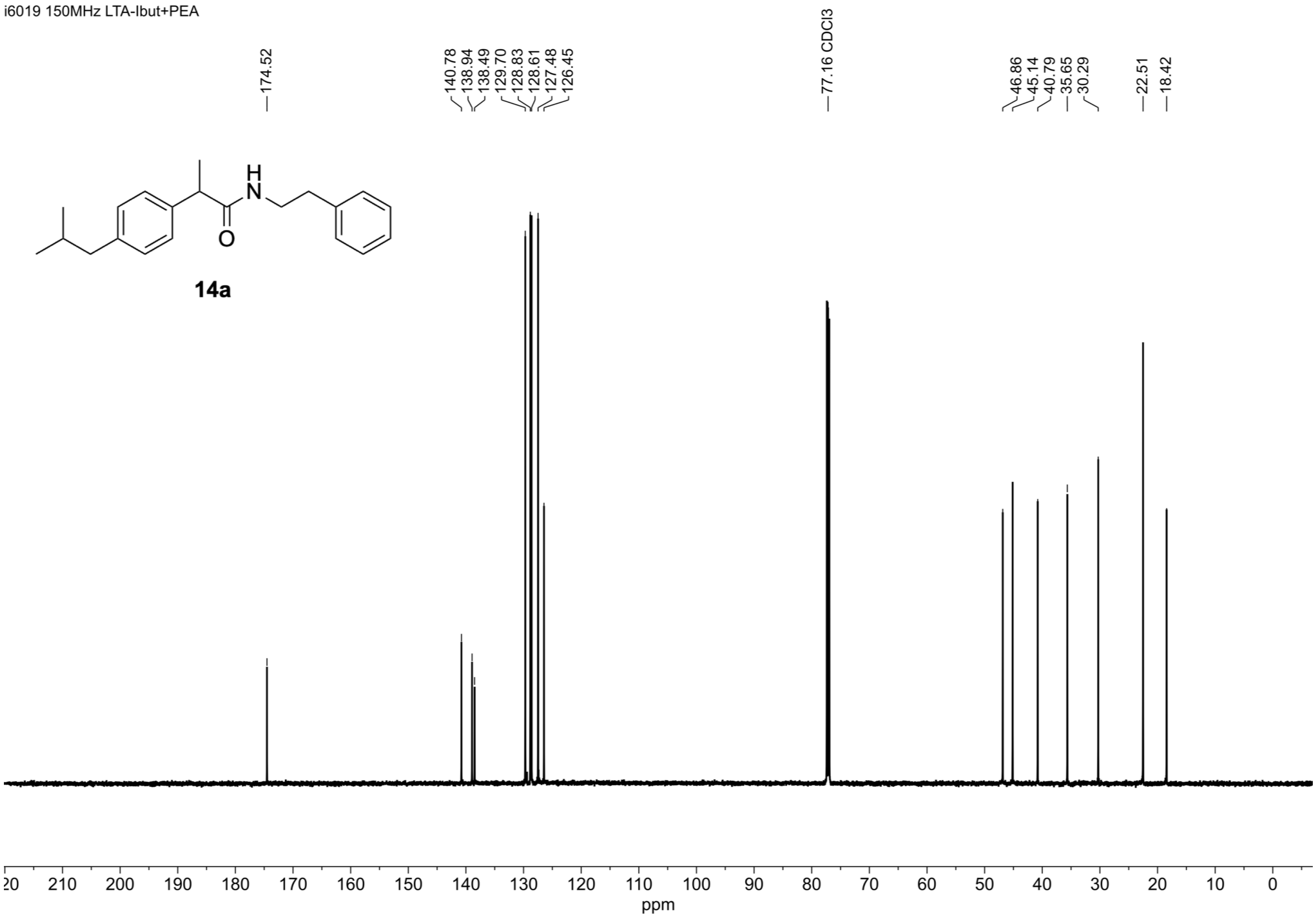

^{13}C NMR spectrum of compound 14a ($CDCl_3$, 151 MHz).

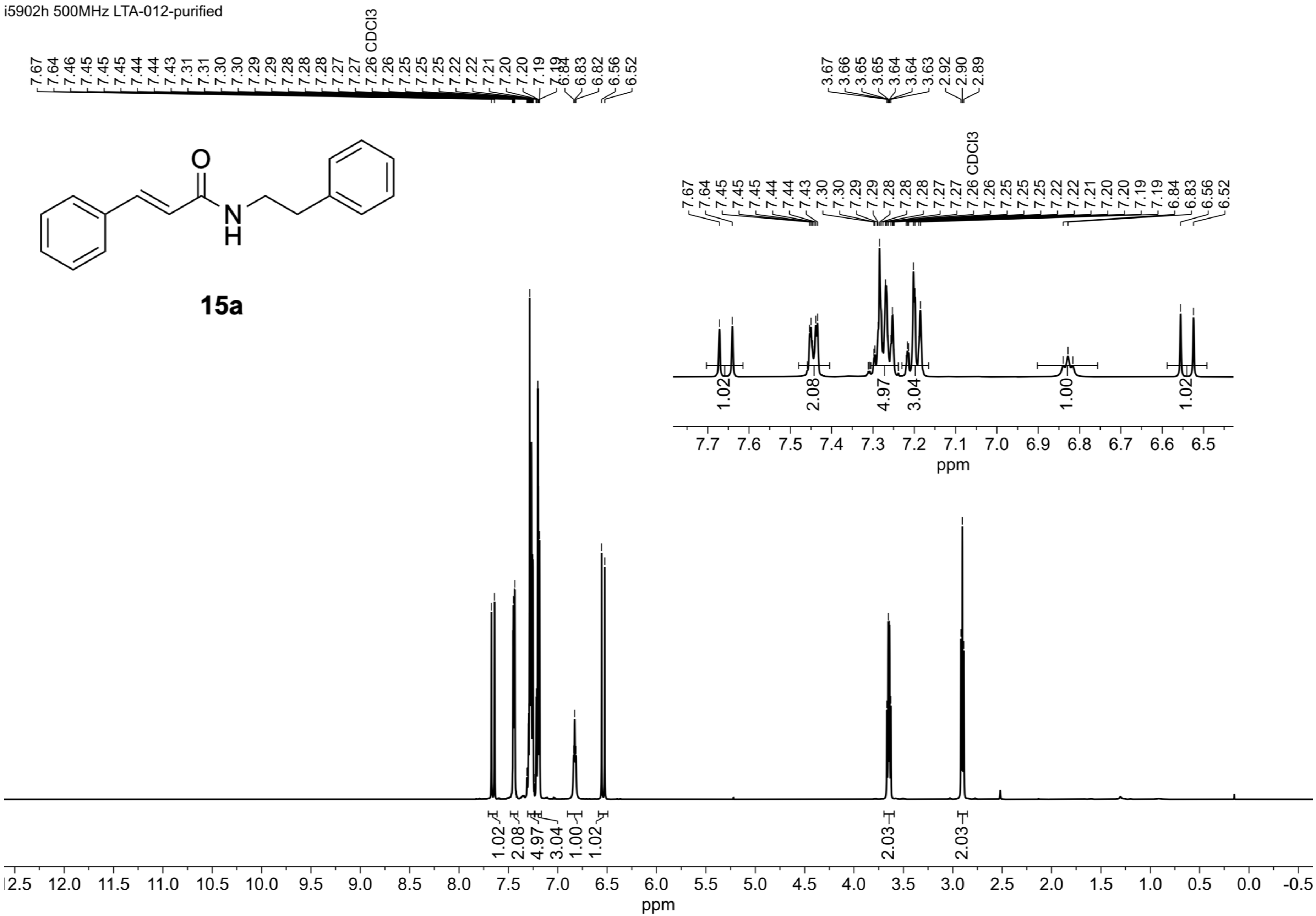

^{1}H NMR spectrum of compound 15a ($CDCl_3$, 500 MHz).

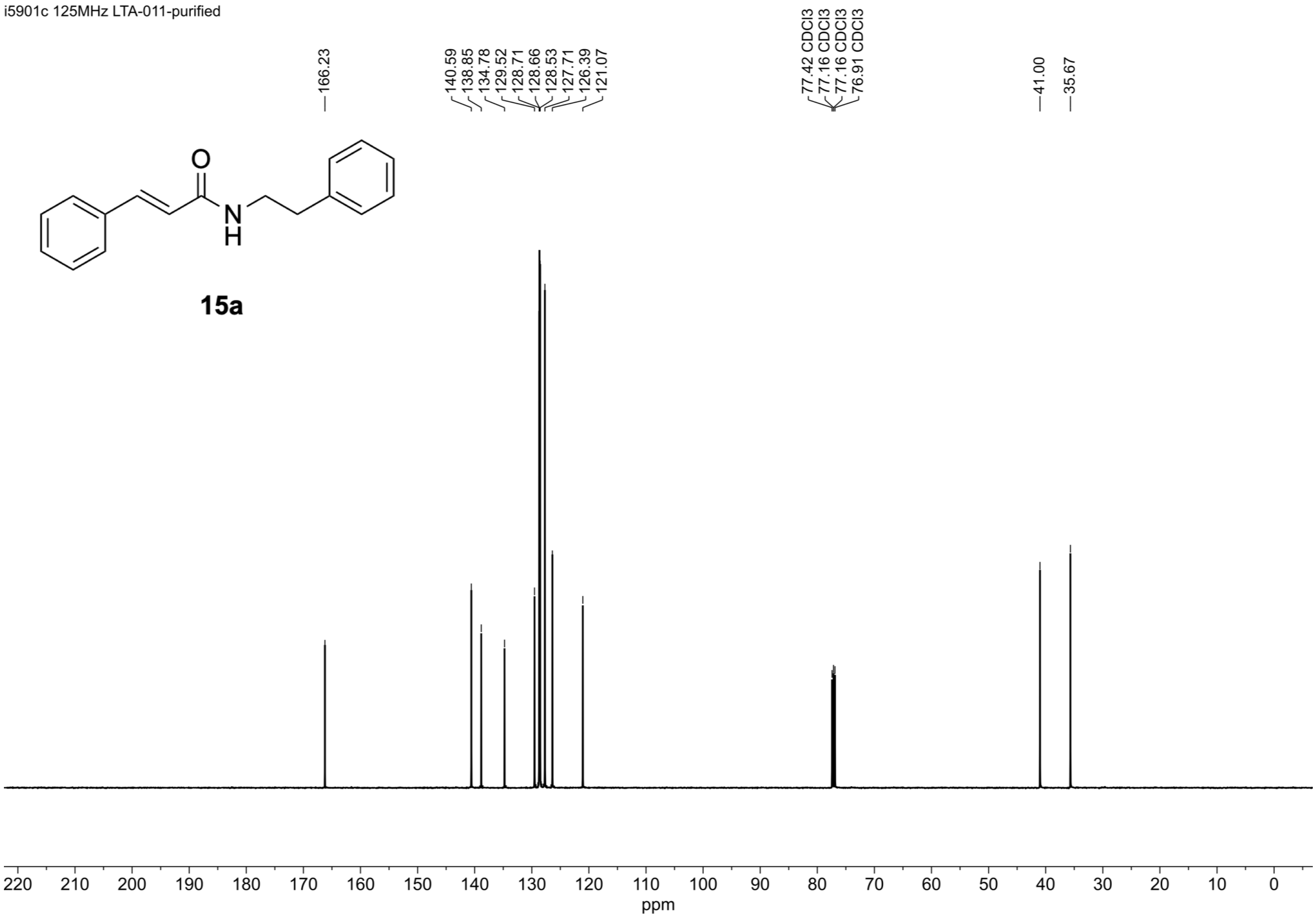

^{13}C NMR spectrum of compound 15a ($CDCl_3$, 126 MHz).

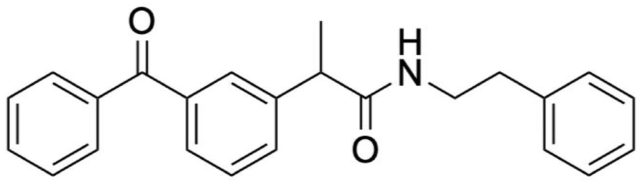

17a

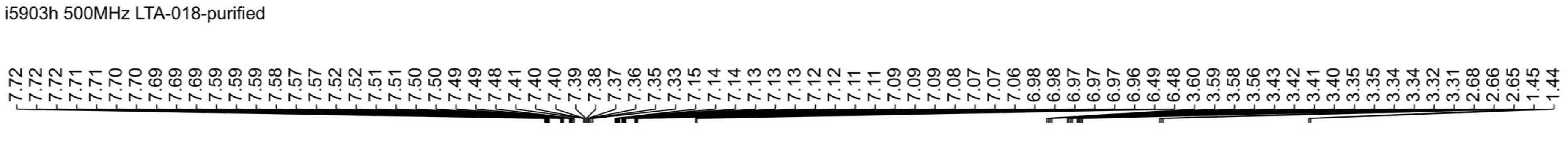

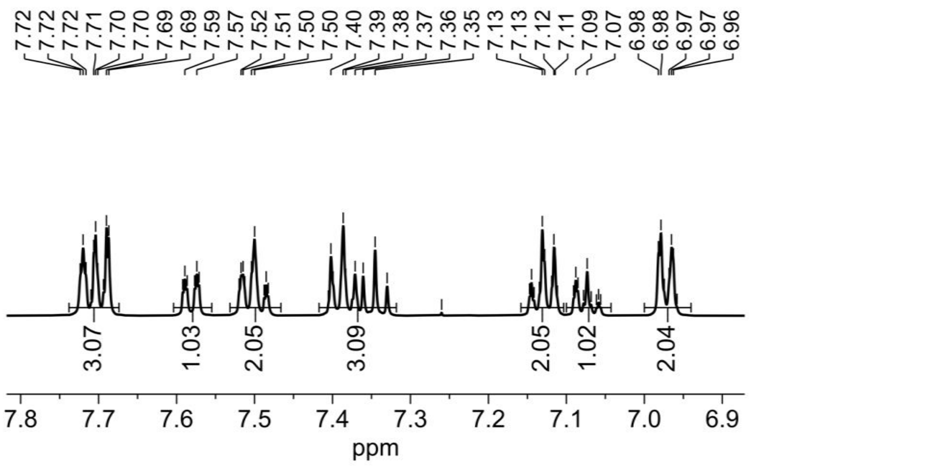

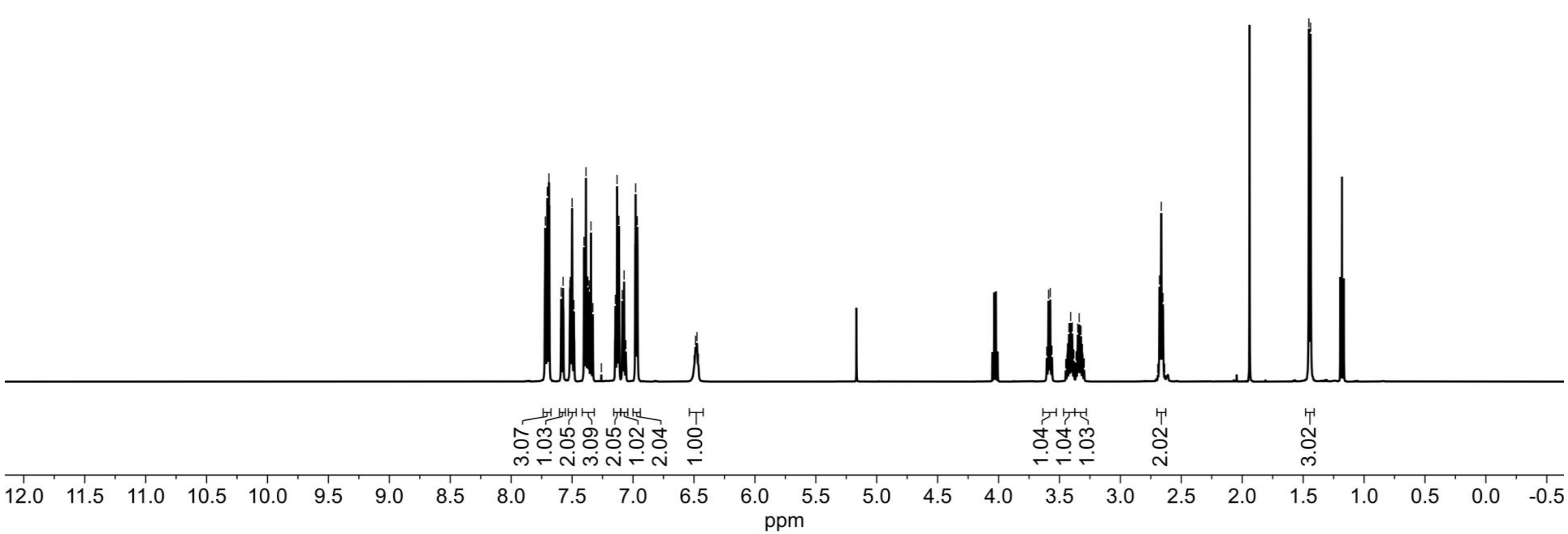

^{1}H NMR spectrum of compound 17a ($CDCl_3$, 500 MHz). Trace EtOAc present at δ 1.18 (t), 1.94 (s), and 4.03 (q).

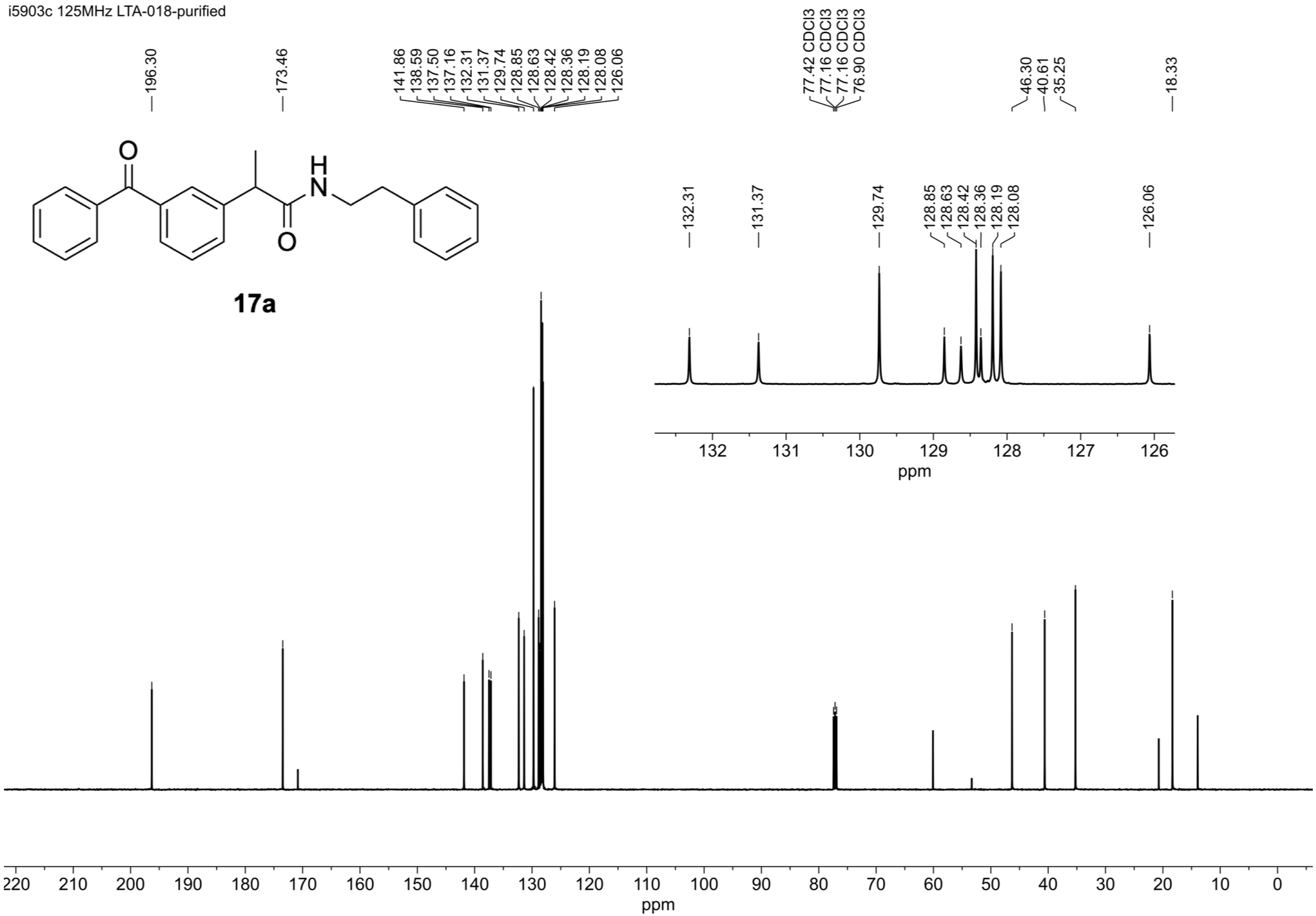

^{13}C NMR spectrum of compound 17a ($CDCl_3$, 126 MHz). Trace EtOAc present at δ 13.93, 20.72, 60.09 and 170.83.

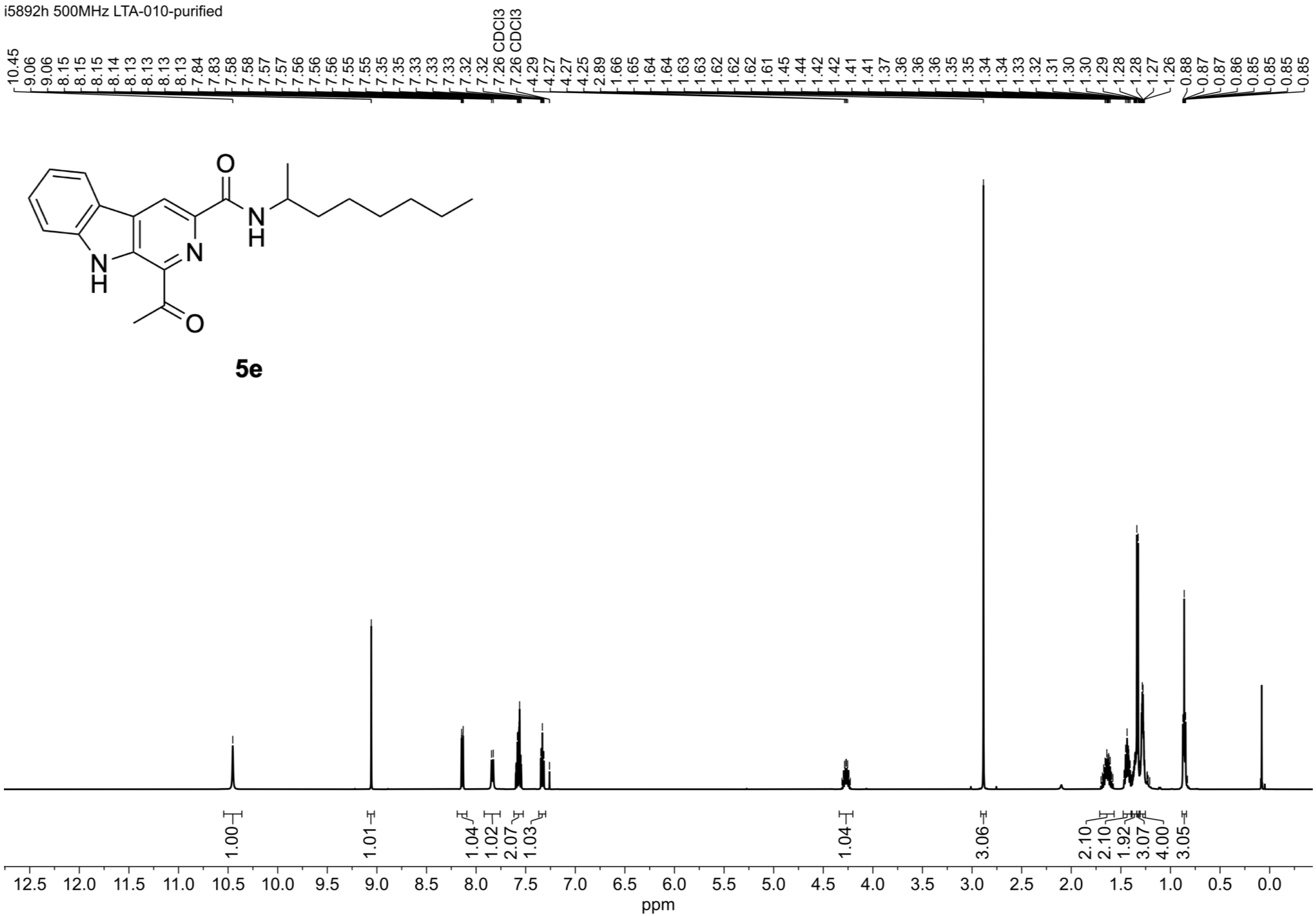

^{1}H NMR spectrum of compound 5e ($CDCl_3$, 500 MHz).

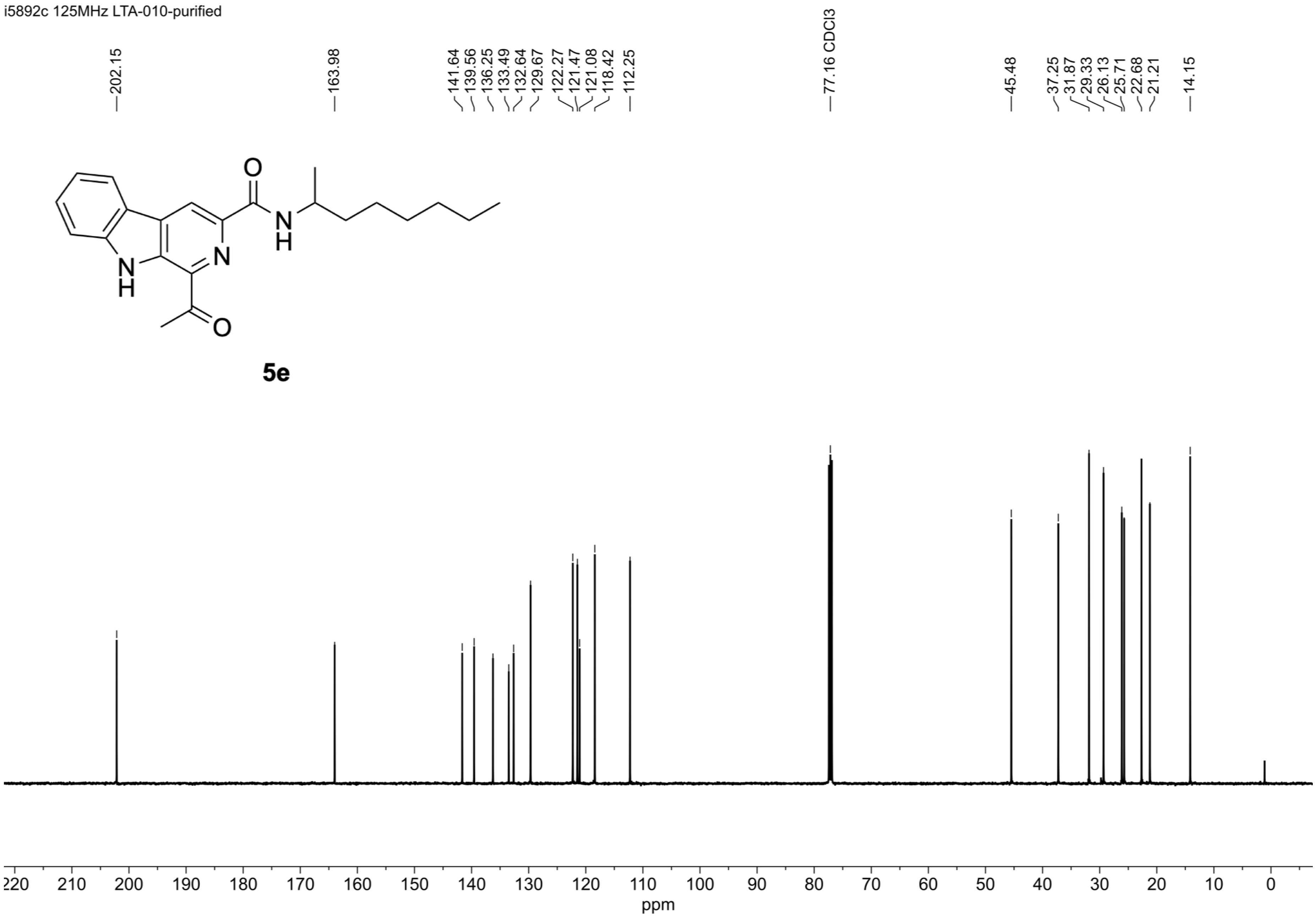

^{13}C NMR spectrum of compound 5e ($CDCl_3$, 126 MHz).

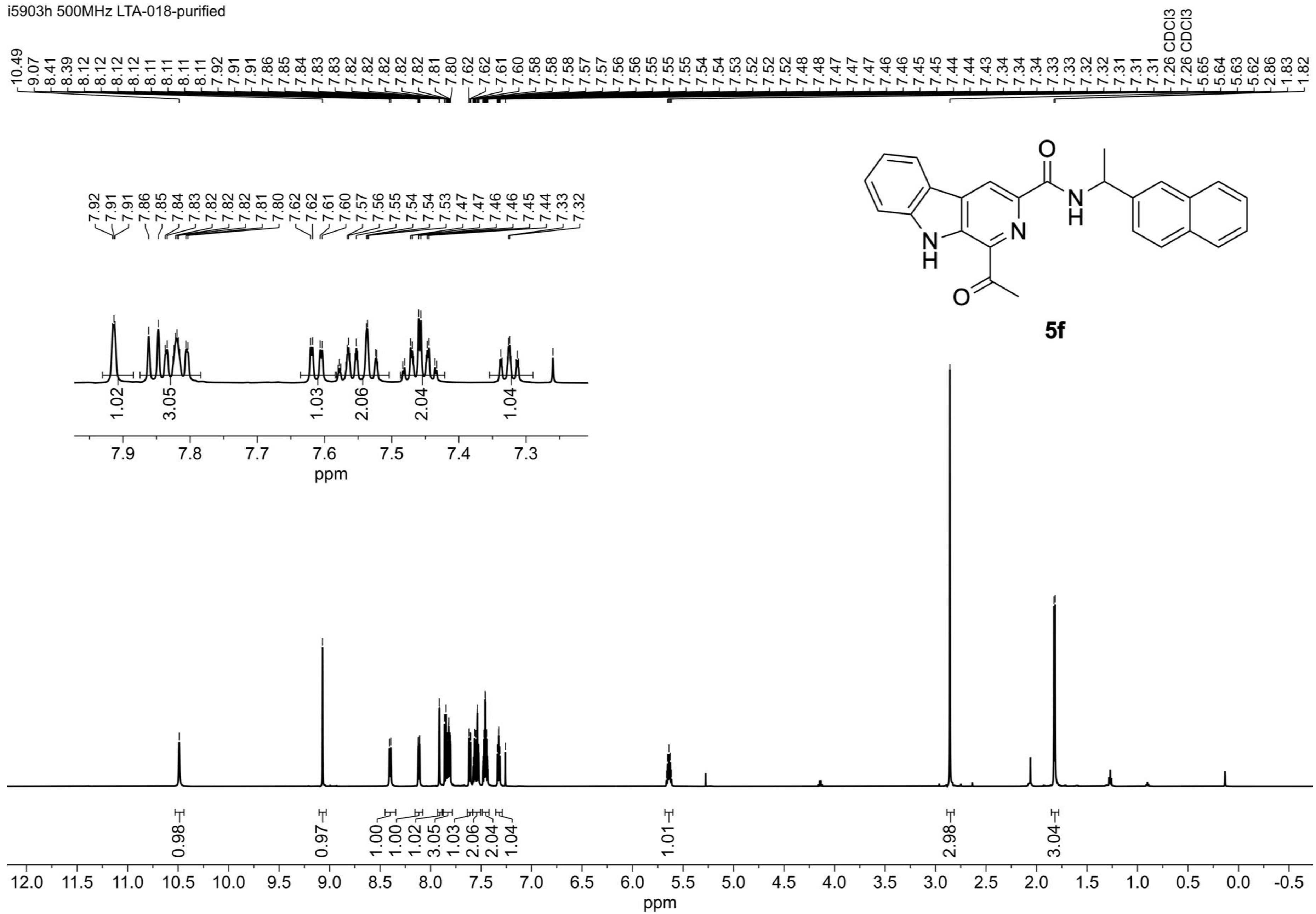

^{1}H NMR spectrum of compound 5f ($CDCl_3$, 600 MHz).

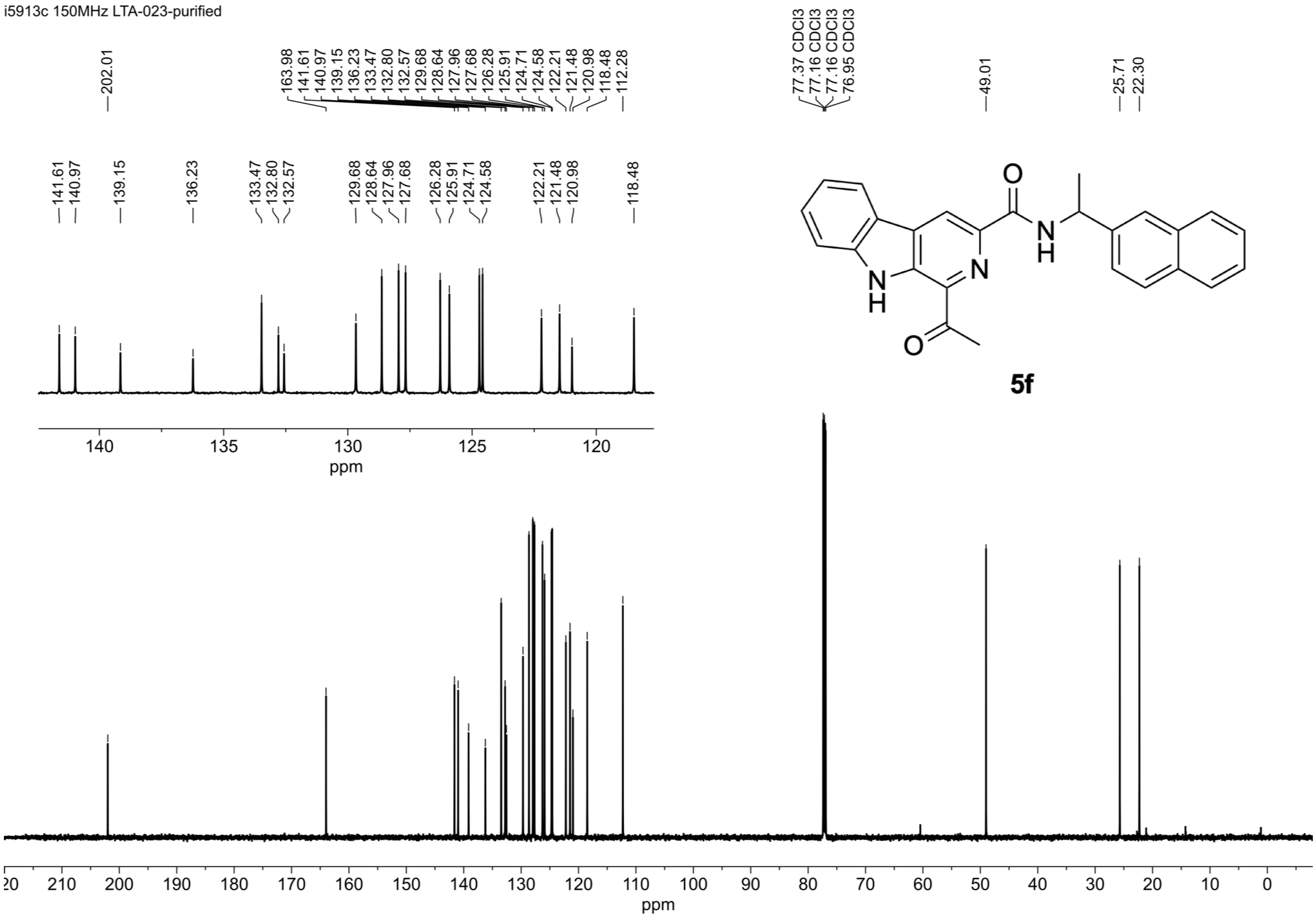

^{13}C NMR spectrum of compound 5f ($CDCl_3$, 151 MHz).

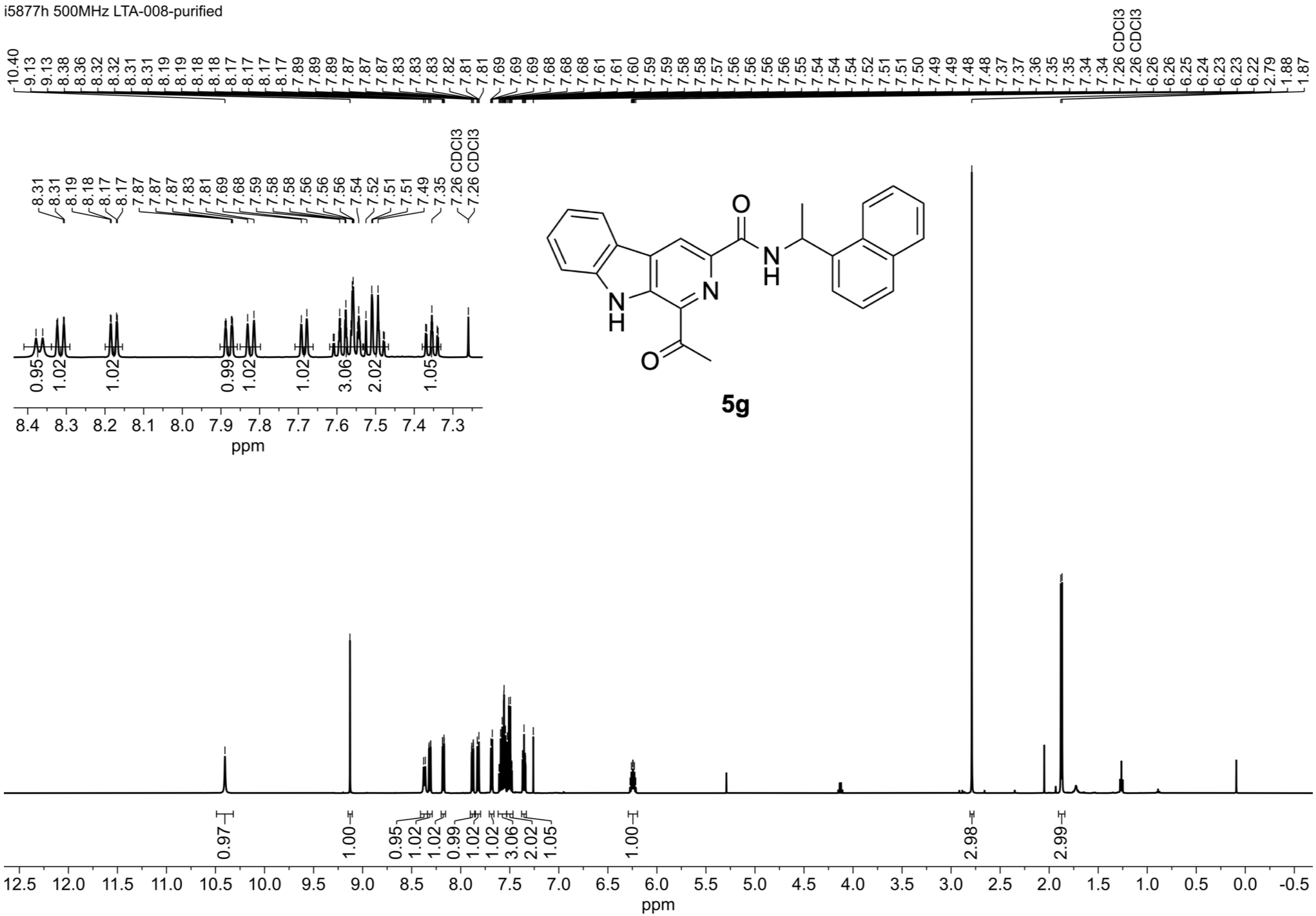

^{1}H NMR spectrum of compound 5g ($CDCl_3$, 500 MHz).

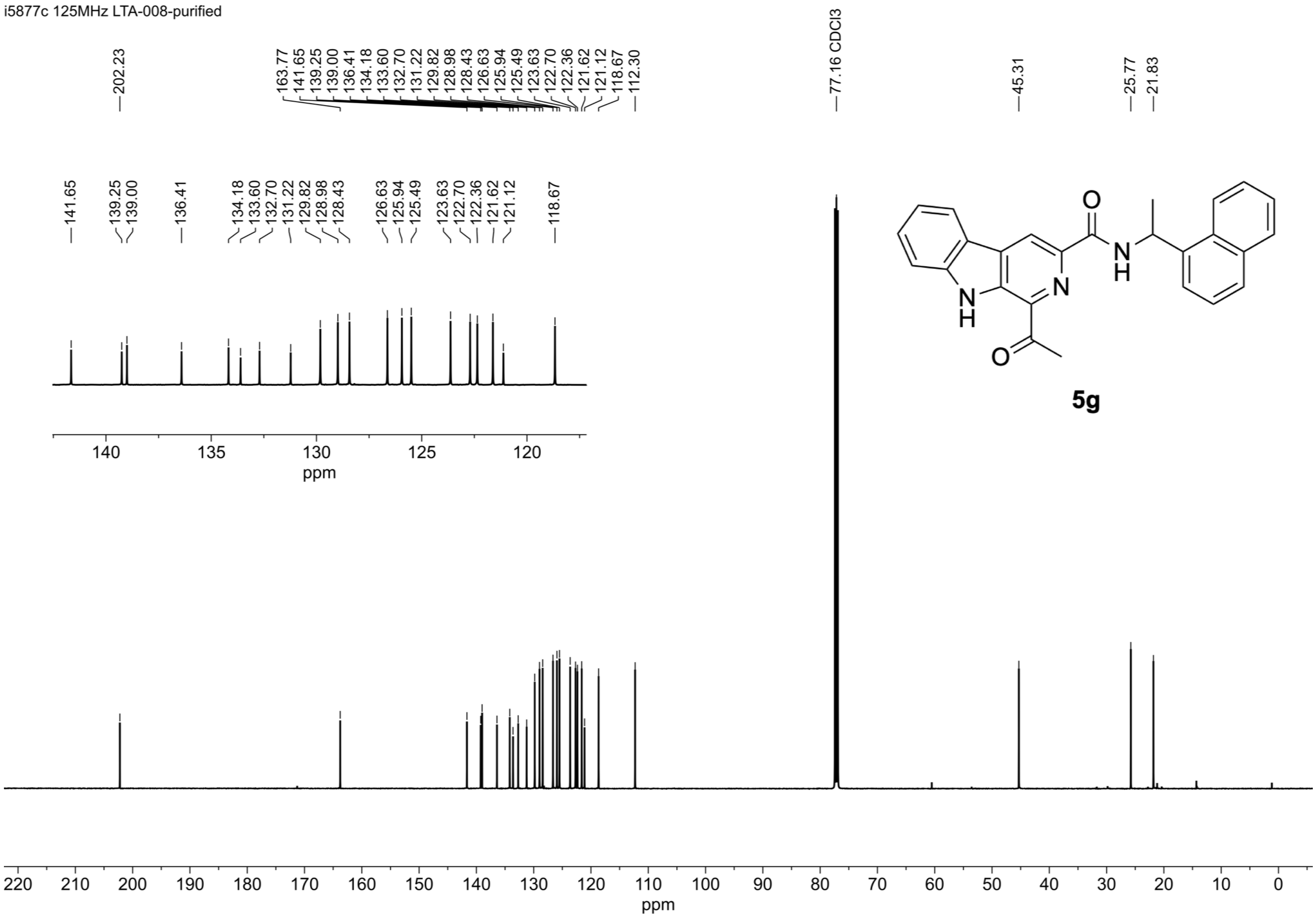

^{13}C NMR spectrum of compound 5g ($CDCl_3$, 126 MHz).

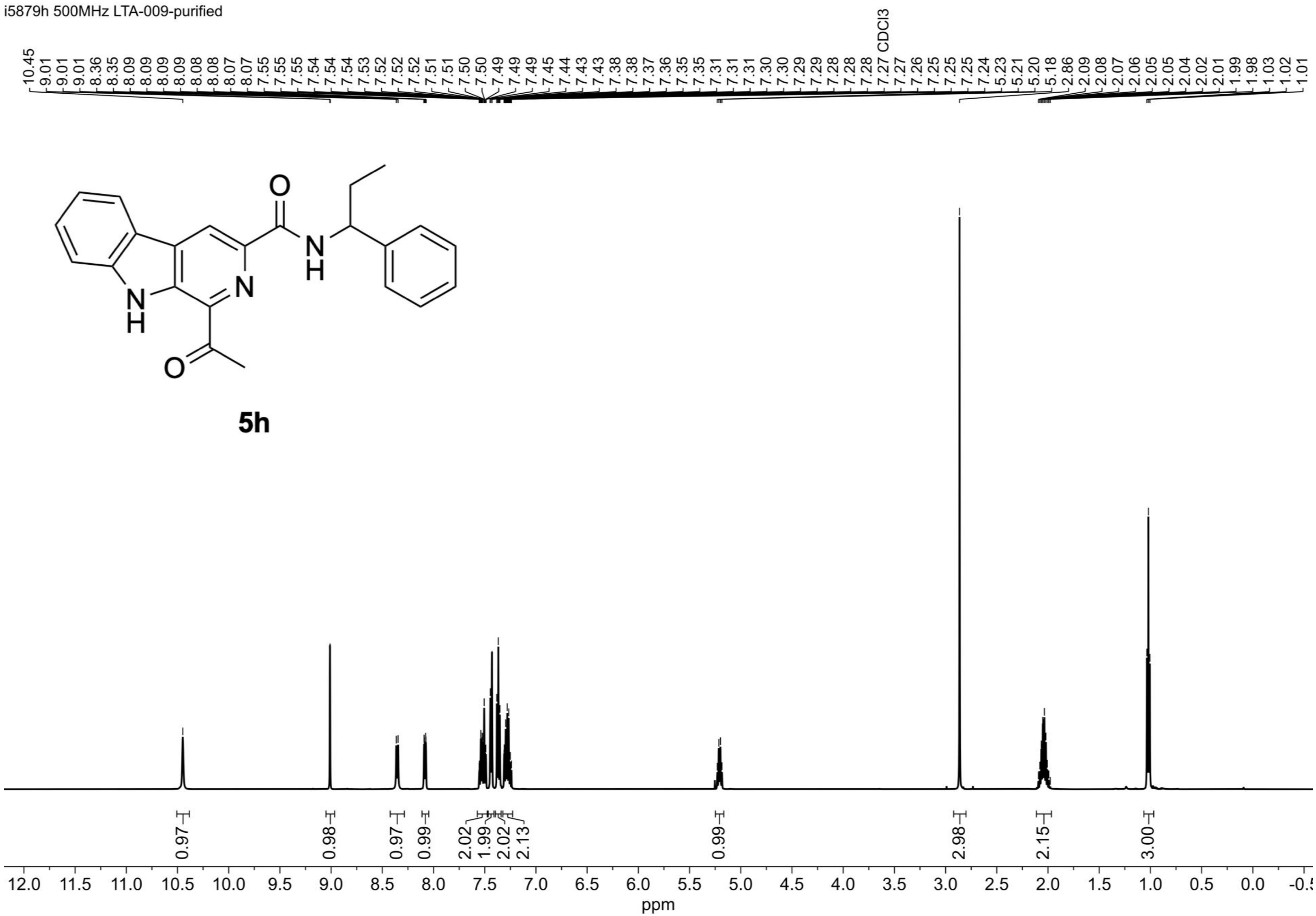

^{1}H NMR spectrum of compound 5h (CDCl$_3$, 500 MHz).

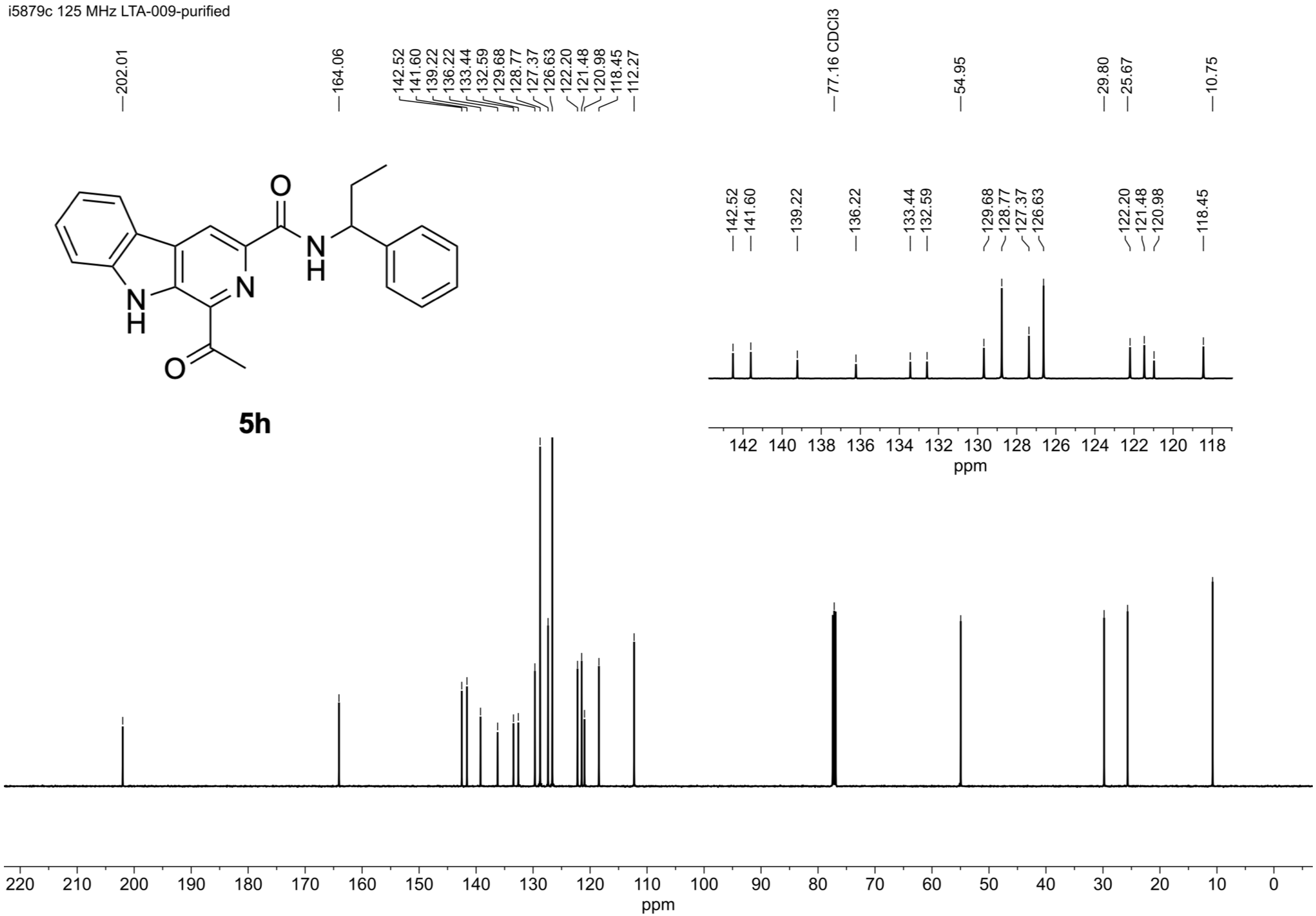

^{13}C NMR spectrum of compound 5h ($CDCl_3$, 126 MHz).

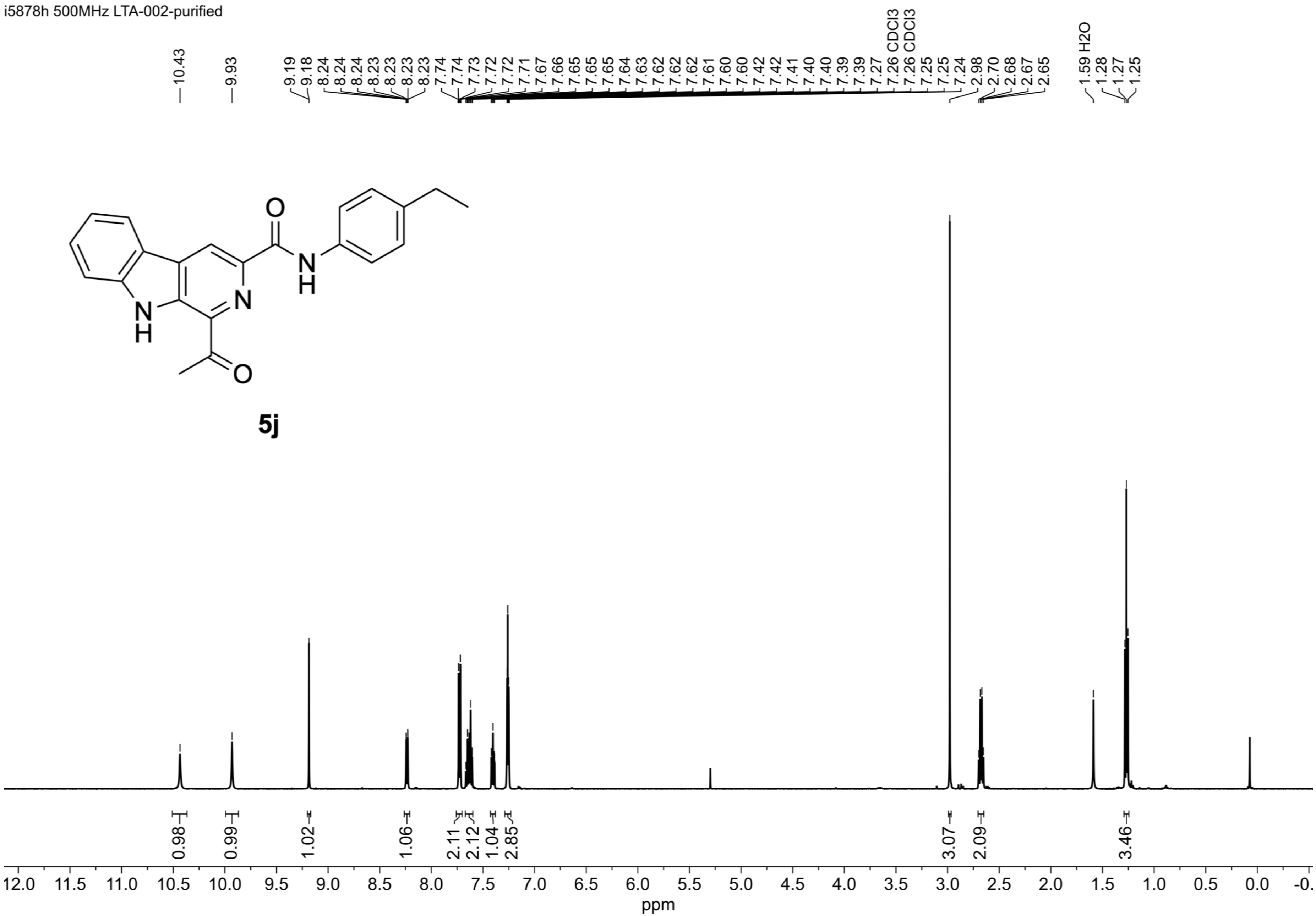

^{1}H NMR spectrum of compound 5j ($CDCl_3$, 500 MHz).

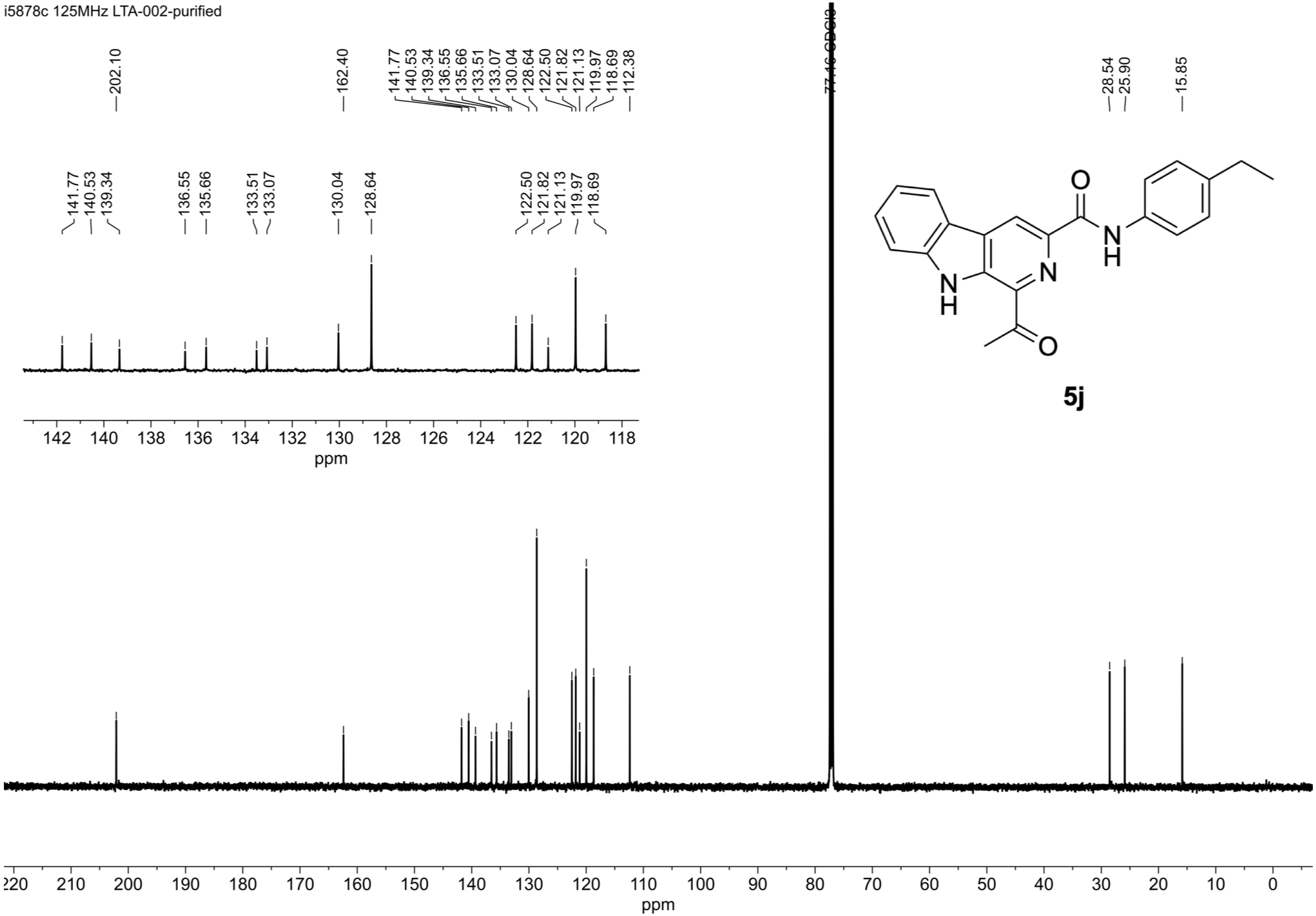

5j

^{13}C NMR spectrum of compound 5j ($CDCl_3$, 126 MHz; carbon signal for $CDCl_3$ is shown off-scale).

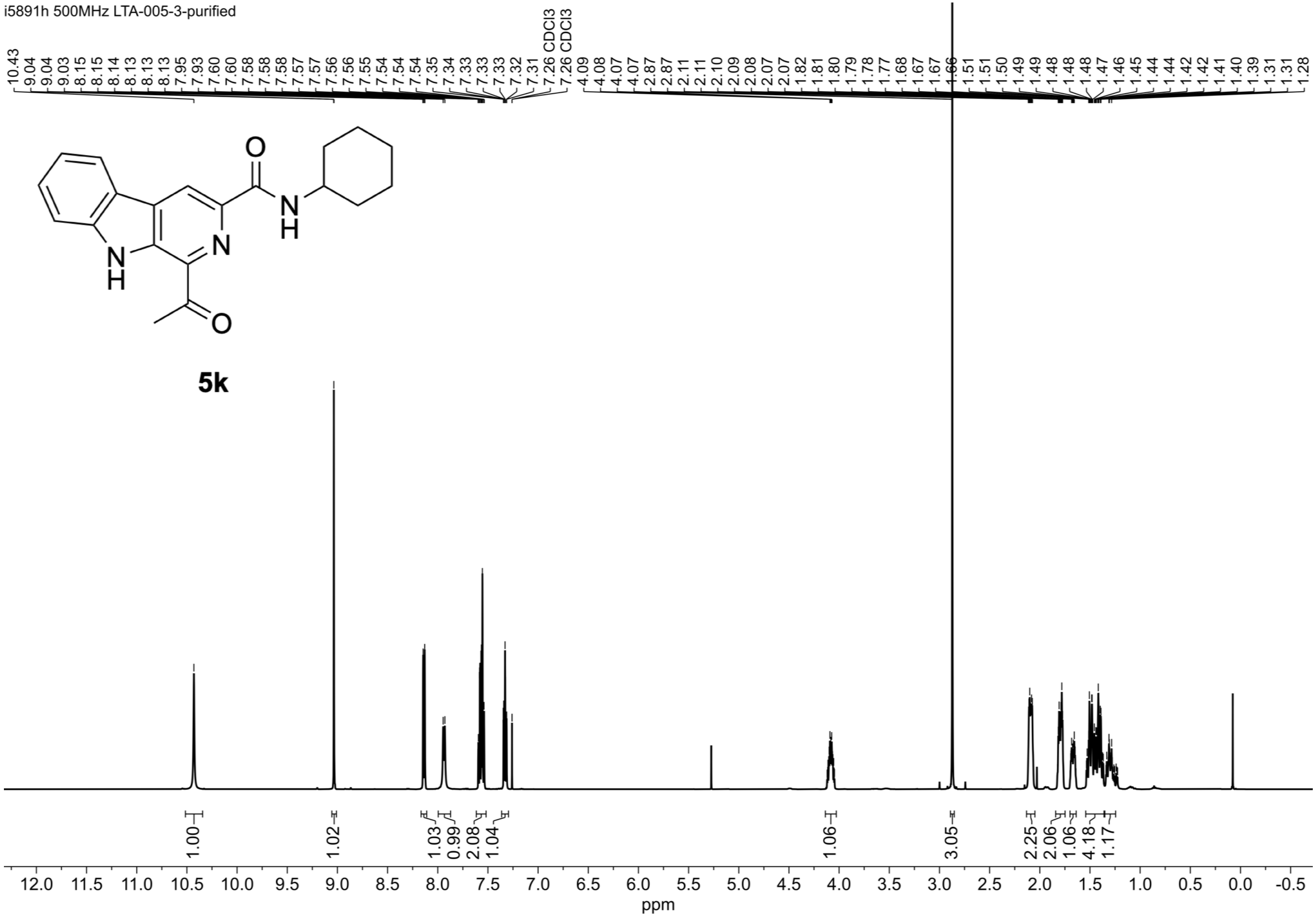

^{1}H NMR spectrum of compound 5k ($CDCl_3$, 500 MHz; the signal at δ 2.87 is shown off-scale).

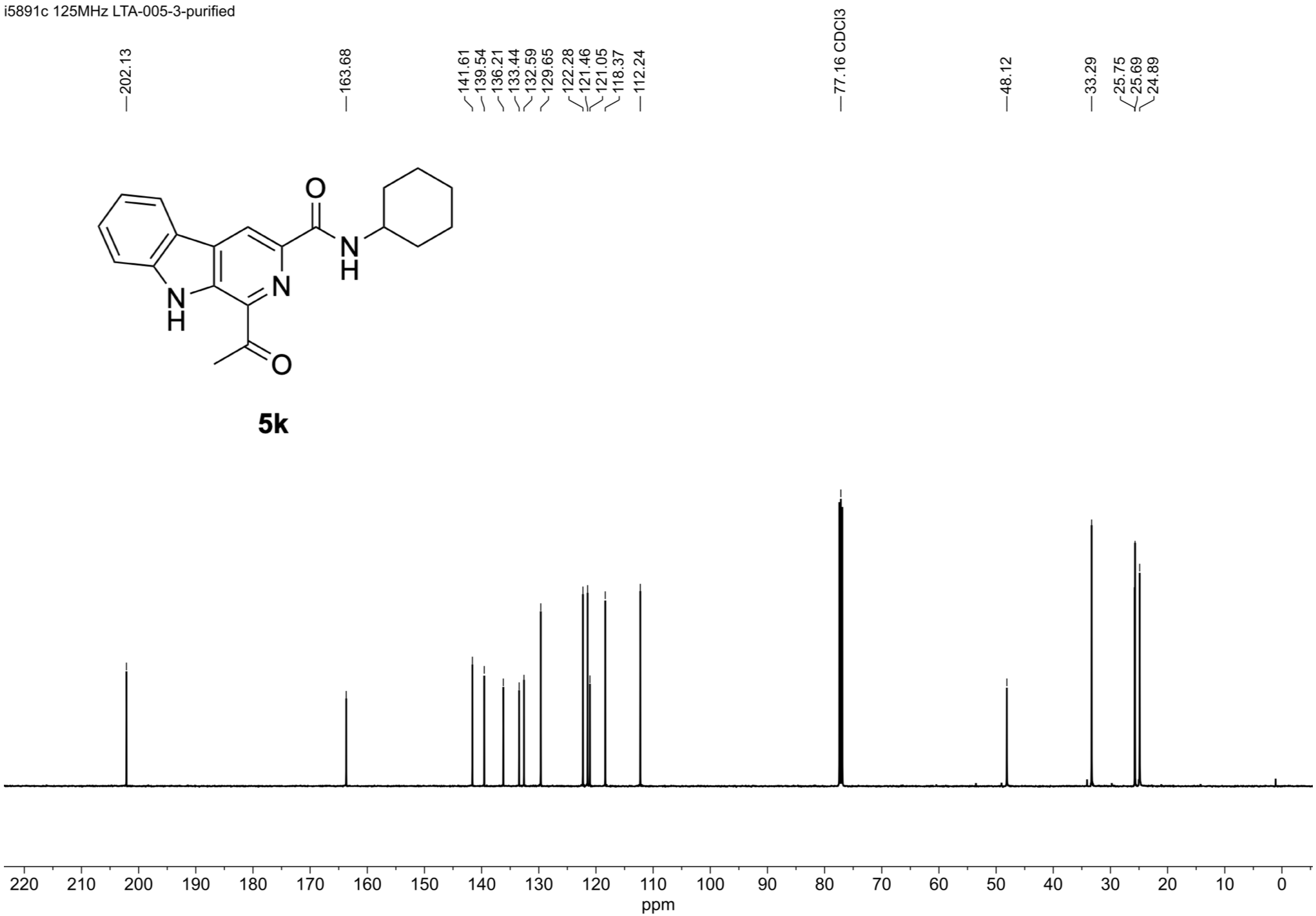

^{13}C NMR spectrum of compound 5k ($CDCl_3$, 126 MHz).

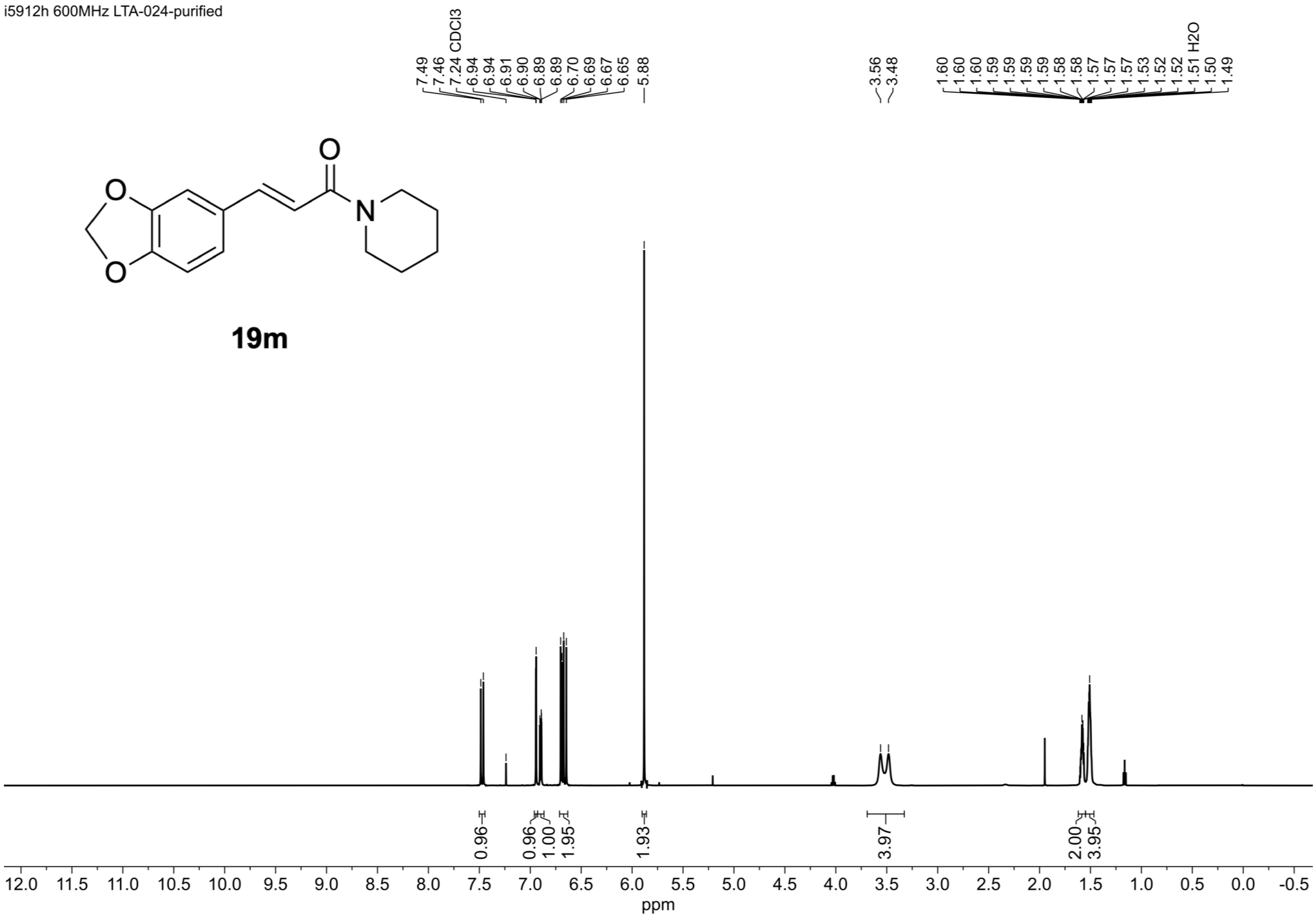

¹H NMR spectrum of compound 19m ($CDCl_3$, 600 MHz).

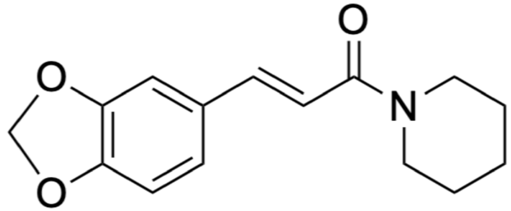

19m

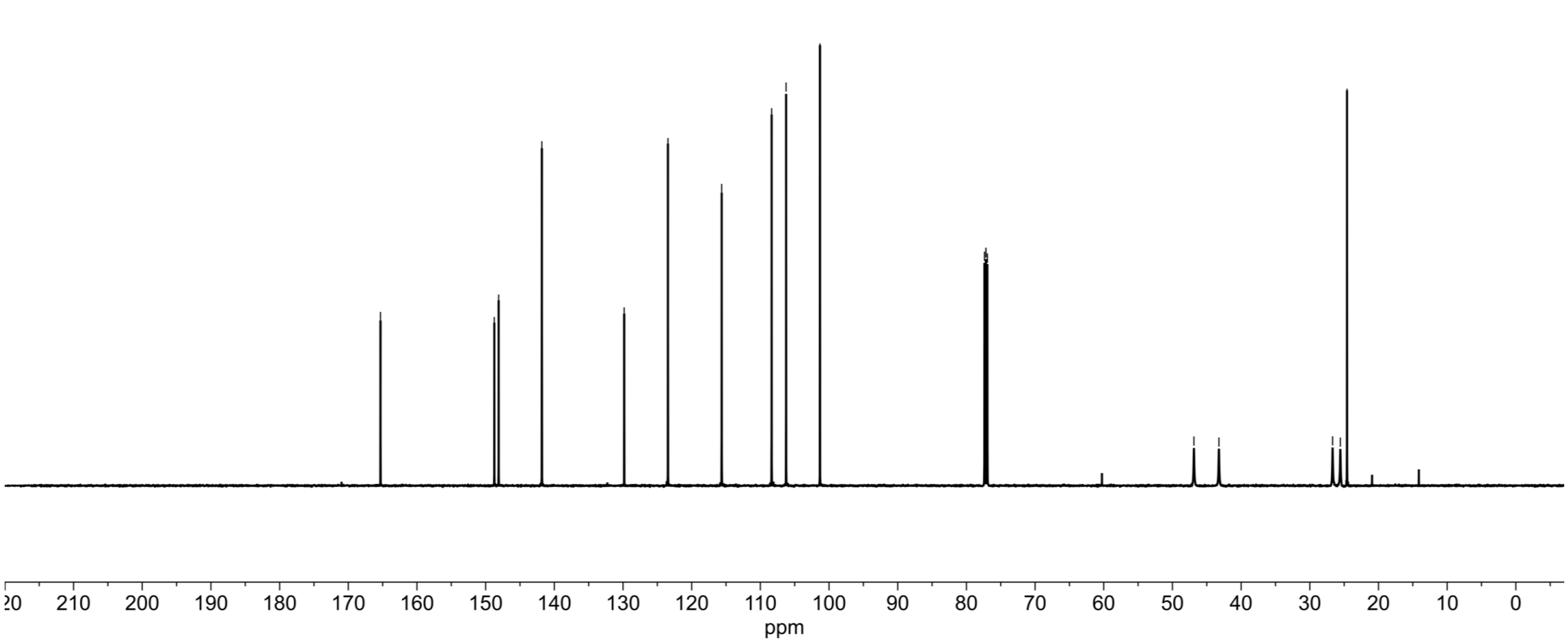

^{13}C NMR spectrum of compound 19m ($CDCl_3$, 151 MHz).

Section 10. References

[1] Thompson, J. D.; Gibson, T. J.; Higgins, D. G. Multiple Sequence Alignment Using ClustalW and ClustalX. *Curr. Protoc. Bioinformatics* **2003**, *00*, 2.3.1-2.3.22.

[2] Robert, X.; Gouet, P. Deciphering key features in protein structures with the new ENDscript server. *Nucleic Acids Res.* **2014**, *42*, W320-W324.

[3] Price, M. N.; Dehal, P. S.; Arkin, A. P. FastTree 2-Approximately Maximum-Likelihood Trees for Large Alignments. *PloS ONE* 2010, **5**, e9490.

[4] Atkin, K. E.; Reiss, R.; Turner, N. J.; Brzozowski, A. M.; Grogan, G. Cloning, Expression, Purification, Crystallization and Preliminary X-ray Diffraction Analysis of Variants of Monoamine Oxidase from Aspergillus niger. *Acta Crystallogr. Sect. F* 2008, **64**, 182-185.

[5] Petchey, M.; Cuetos, A.; Rowlinson, B.; Dannevald, S.; Frese, A.; Sutton, P. W.; Lovelock, S.; Lloyd, R. C.; Fairlamb, I. J. S.; Grogan, G. The Broad Aryl Acid Specificity of the Amide Bond Synthetase McbA Suggests Potential for the Biocatalytic Synthesis of Amides. *Angew. Chem. Int. Ed.* **2018**, *57*, 11584-11588.

[6] Petchey, M. R.; Rowlinson, B.; Lloyd, R. C.; Fairlamb, I. J. S.; Grogan, G. Biocatalytic Synthesis of Moclobemide Using the Amide Bond Synthetase McbA Coupled with an ATP Recycling System. *ACS Catal.* **2020**, *10*, 4659-4663.

[7] Still, W. C.; Kahn, M.; Mitra, A. Rapid Chromatographic Technique for Preparative Separations with Moderate Resolution. *J. Org. Chem.* **1978**, *43*, 2923-2925.

[8] Kabsch, W. XDS. *Acta Crystallogr. Sect. D* **2010**, *66*, 125-132.

[9] Evans, P. Scaling and Assessment of Data Quality. *Acta Crystallogr., Sect. D:* **2006***, 62*, 72-82.

[10] Winter, G. Xia2: An Expert System for Macromolecular Crystallography Data Reduction. *J. Appl. Crystallogr.* **2010**, *43*, 186-190.

[11] Vagin, A.; Teplyakov, A. MOLREP: An Automated Program for Molecular Replacement. *J. Appl. Crystallogr.* **1997**, *30*, 1022-1025.

[12] Emsley, P. & Cowtan, K. Coot: Model-building Tools for Molecular Graphics. *Acta Crystallogr., Sect. D.* **2004***, 60*, 2126-2132.

[13] Murshudov, G. N.; Vagin, A. A.; Dodson, E. J. Refinement of Macromolecular Structures by the Maximum-likelihood Method. *Acta Crystallogr., Sect. D.* 1997, **53**, 240-255.

[14] Waterhouse, A.; Bertoni, M.; Bienert, S.; Studer, G.; Tauriello, G.; Gumienny, R.; Heer, F. T.; de Beer, T. A P.; Rempfer, C.; Bordoli, L.; et al. SWISS-MODEL: Homology Modelling of Protein Structures and Complexes. *Nucleic Acids Res.* **2018**, *46*, W296-W303.

[15] Trott, O.; Olson, A. J. Software News and Update AutoDock Vina: Improving the Speed and Accuracy of Docking with a New Scoring Function, Efficient Optimization, and Multithreading. *J. Comp. Chem.* **2010**, *31*, 455-461.

[16] The PyMOL Molecular Graphics System, Version 2.5. Schrödinger, LLC.

[17] Nagaiah, B.; Narsaiah, A. V. Concise and Efficient Synthesis of Cinepazide. *Synth. Commun.* **2014**, *44*, 1227-1231.
